# Construction of Commercial Sweet Cherry Linkage Maps and QTL Analysis for Trunk Diameter

**DOI:** 10.1371/journal.pone.0141261

**Published:** 2015-10-30

**Authors:** Jing Wang, Kaichun Zhang, Xiaoming Zhang, Guohua Yan, Yu Zhou, Laibao Feng, Yang Ni, Xuwei Duan

**Affiliations:** 1 Institute of Forestry and Pomology, Beijing Academy of Agricultural and Forestry Sciences, Beijing, 100093, China; 2 Beijing Engineering Research Center for Deciduous Fruit Trees, Beijing, 100093, China; 3 Key Laboratory of Biology and Genetic Improvement of Horticultural Crops (North China), Ministry of Agriculture, P.R. China, Beijing, 100093, China; 4 Laboratory of Photosynthesis and Environmental Molecular Physiology, Institute of Botany, Chinese Academy of Sciences, Xiangshan Nanxincun 20, Beijing, 100093, China; USDA-ARS-SRRC, UNITED STATES

## Abstract

A cross between the sweet cherry (*Prunus avium*) cultivars ‘Wanhongzhu’ and ‘Lapins’ was performed to create a mapping population suitable for the construction of a linkage map. The specific-locus amplified fragment (SLAF) sequencing technique used as a single nucleotide polymorphism (SNP) discovery platform and generated 701 informative genotypic assays; these, along with 16 microsatellites (SSRs) and the incompatibility (*S*) gene, were used to build a map which comprised 8 linkage groups (LGs) and covered a genetic distance of 849.0 cM. The mean inter-marker distance was 1.18 cM and there were few gaps > 5 cM in length. Marker collinearity was maintained with the established peach genomic sequence. The map was used to show that trunk diameter (TD) is under the control of 4 loci, mapping to 3 different LGs. Different locus influenced TD at a varying stage of the tree’s development. The high density ‘W×L’ genetic linkage map has the potential to enable high-resolution identification of QTLs of agronomically relevant traits, and accelerate sweet cherry breeding.

## Introduction

The economically significant fruit tree species sweet cherry (*Prunus avium*) is self-incompatible, and therefore has a highly heterozygous genetic background. However, sweet cherry has a small genome size (338 M), just two times that of *Arabidopsis* [1]. It shares the same chromosome number (2n = 2x = 16) with its relatives peach (*P*. *persica*), almond (*P*. *dulcis*) and apricot (*P*. *armeniaca*). As with other woody perennials, sweet cherry has a long juvenile period (average of 6 years) and, thus requiring long-term breeding strategies. Genetic linkage maps provide opportunities for unlocking the complex genetics of quantitatively inherited traits through the localization of quantitative trait loci (QTL), and serve as a repository of markers useful in marker-assisted breeding (MAB) [2–4]. Several intact genetic linkage maps for sweet cherry have been assembled [5–8,9] (S1 Table). While, some of these have been based on gel-based DNA assasys, more recently a SNP approach has been exploited for mapping [10–14].

Basing marker discovery on a reduced representation libraries (RRL) has proven to be an efficient and cost-saving strategy across a wide range of animal, plant and microorganism species [15–20]. One such application, denoted “specific-locus amplified fragment sequencing” (SLAF-seq) [21], has been successfully developed for markers developing in at least three plant species [22–24]. Meanwhile, the acquisition of full genome sequence of peach [25, 26 and www.rosaceae.org/peach/genome] has allowed advantage to be taken of the extensive synteny which has been shown to exist in the genus *Prunus* [27].

Here, the SLAF-seq method was exploited to rapidly provide the large number of markers needed to generate a high density linkage map of sweet cherry. The map was based on a segregating population bred from a cross between two elite sweet cherry cultivars ‘Wanhongzhu’ (‘W’) and ‘Lapins’ (‘L’). ‘W’ fruit matures about 10 days later than those of ‘L’, and forms fruits which are large and of excellent quality. In three continuous years, a broad variety in tree habit and fruit characters were observed and scored in the progeny. The resulting linkage map was used to explore the genetic determination of the trunk diameter (TD), a trait which has been correlated with tree vigor, resistance and fruit yield [28]. Trees which have a large TD, along with a wide brunch angle and lower tree height are seen as advantageous in the context of crop management and so are favored by breeders. However, unlike tree height and brunch angle, TD cannot be manipulated by prune. Thus a genetic means of controlling this trait would aid the genetic advancement of the crop.

## Materials and Methods

### Plant materials

The cross between ‘W’ as female and ‘L’ as male was made in 2007. ‘W’ (*S*_*6*_ *S*_*9*_) was a seedling of ‘19–11’, which was a progeny of ‘Bing’ and ‘Sunburst’. ‘L’ (*S*_*1*_ *S*_*4*_’) was a self-compatible cultivar, widely used in cherry breeding. A population of 860 F_1_ progeny was planted in rows in which the intra-row spacing was 1.0 m and the inter-row spacing was 3.5 m at the Shangzhuang experiment station of institute of forestry and pomology’s in Beijing in the spring of 2008. Because these field experiments were done in the test bases of Institute of Forestry and Pomology. And, the field studies did not involve endangered or protected species. No specific permissions were required for field experiment locations/activities. A subset of 100 of the population was selected as the linkage mapping population, ensuring a balance of *S* allele representation: 16 progeny were of genotype *S*_*4*_ *’S*_*6*_, 18 were *S*_*4*_ *’S*_*9*_, 23 were *S*_*1*_ *S*_*6*_ and 22 were *S*_*1*_ *S*_*9*_.

### Marker development

The methods used to generate SLAF markers (pre-design, library construction, Illumina sequencing, SNP discovery and genotyping) largely followed those described in [21]. Since the full genome sequence of sweet cherry has not yet been acquired, that of peach were used for *in-silico* analysis of restriction enzyme recognition sites. Following the construction of the library, pair-end sequencing targeting fragments in the size range 400~500 bp was performed by Beijing Biomarker Technologies Co. Ltd (www.biomarker.com.cn/english/) using a genome analyzer II instrument (Illumina, Inc; San Diego, CA, U.S.). The resulting raw sequence reads were processed by custom Perl scripts (E.A.J.) to optimize read number and to reduce artifacts (five bases with Q score < 20). After the SLAF reads were assigned into individual plants according to their given index, sequences sharing > 96% identity were considered as a single SLAF locus. Only sequences represented at least 107 times and harboring at most 4 genotypes were accepted as representing a high quality SLAFs; those harboring 2–4 genotypes were then carried forward for mapping. In addition, a set of 53 published SSR assays, along with 150 SSR assays designed from sequences represented in the peach genome (www.rosaceae.org/node/355) were used to fingerprint the two parental cultivals and the members of the mapping population. The PCR conditions were identical to those published [5].

### Linkage map construction

Based on the parental genotypes, the informative set of SLAF loci formed 5 allelic classes (ab×cd, ef×eg, hk×hk, lm×ll and nn×np). Sixty-three aa×bb type markers were convertible into lm×ll, nn×np or ef×eg types based on the separation pattern produced by the F_1_ progeny. Homozygosity was confirmed only where a sequence depth of at least three was recorded; heterozygosity was confirmed when either the sequence depth of the least frequent allele was > 3 or the depth ratio of the two alleles was > 1:6 or 2:18, otherwise it would be considered homozygous or missing. For the informative SSR loci, the allelic status of the mapping population progeny fell into the 3 classes hk×hk, lm×ll and nn×np.

The full set of genotypic data (SLAF, SSR and *S* gene) were combined and then subjected to analysis using JoinMap^®^4.0 software [29], employing the “CP” model. A two-step strategy was implemented, in which a framework map was first generated based on a set of the highest quality marker (missing value rate < 30% and fitting the expected segregation ratio with P-value < 0.05), applying a stringent LOD thresholds (5.0) for grouping. Recombination frequencies were converted to cM using the Kosambi function regression mapping method [30]. The goodness-of-fit jump threshold for removal a loci was set to 5.0, the ‘suspect linkage’ and ‘genotype probabilities’ tools were applied to improve the reliability of the map. Linkages with a LOD = 3 were used for mapping and the second round maps were selected. Then, a strongest crosslink values (SCLs) of 1 were applied to assign ungrouped loci to their most likely LG [29]. By using similar parameters and procedures, a ‘W×L’ map was assembled. Synteny analysis was performed between the frame map and the ‘W×L’ map repeatedly to test how marker rearrangement was affected by addition of newly-added markers. In addition, Sequence alignment between SLAF sequences and the peach genome sequence was based on a similarity threshold of 80%. Each of the sweet cherry LGs was related by name to the eight peach LGs, according to their content of anchored SLAF markers. A Pearson correlation analysis and the synteny analysis of anchored marker positions between ‘W×L’ genetic linkage map and peach physical map were performed using SPSS software.

### QTL analysis for TD

TDs were measured at a fixed point of 20 cm above ground in four continuous years. Anural trunk net growth (ATNG) was calculated from the difference between TD year_(n+1)_ and TD year_(n)_. The mapping population’s statistical parameters were obtained using the SPSS 16.0 software. QTL detection was carried out using the MapQTL 4.0 software, using the interval mapping (IM) procedure [29]. The genome-wide LOD score threshold for QTL significance was determined using the permutation test (PT, [31]), from which a LOD of 3.0 was set. QTL positions were drawn using MapChart [32].

## Results

### Informative markers

The intention was to construct a sweet cherry linkage map in which the mean interval between adjacent markers was about 1 cM. Since the estimated genetic length of sweet cherry genome was 600–800 cM (S1 Table), a target of 600–800 markers was chosen. Based on the known level of marker polymorphism between sweet cherry varieties (10–15%) [5–8, 9], about 6000–8000 SLAF fragments should be produced by digestion in SLAF-seq method. In peach, the restriction enzyme combination *Bfa I+Mse I* was predicted to produce 12,856 well distributed DNA fragments of size range 380–430 bp (S1 Fig) with the expected repetitive sequences might be controlled within 1.13%, so this was selected an appropriate combination for sweet cherry. A total of ~ 4.7 G of raw sequence data (NIH Short Read Archive accession number: SRP063722), derived from 31,048,840 pair-end (PE) reads was obtained, resulting in the identification of 14,634 high quality SLAFs. The coverage of these loci was 27×in ‘L’, 20× in ‘W’ and average 4.9× among the mapping population progeny. Of these, 1,838 (12.6%) were classified as polymorphic (S2 Table), in agreement with the predicted range of marker informativeness. Repeated sequences accounted for 4.97% of the high quality SLAFs, a slightly higher frequency than in peach. The final set of high quality SLAFs with full integrity in parents and 82% integrity in progenies was 953. With respect to SSRs, 28 (13.79%) were informative. The *S* gene segregated consistently with its inheritance from an ab×cd cross. Thus in all, segregation data at 982 loci (953 SLAFs, 28 SSRs and *S*) were available for the construction of the linkage map (S3 Table).

### The sweet cherry linkage map

**Fig 1:**
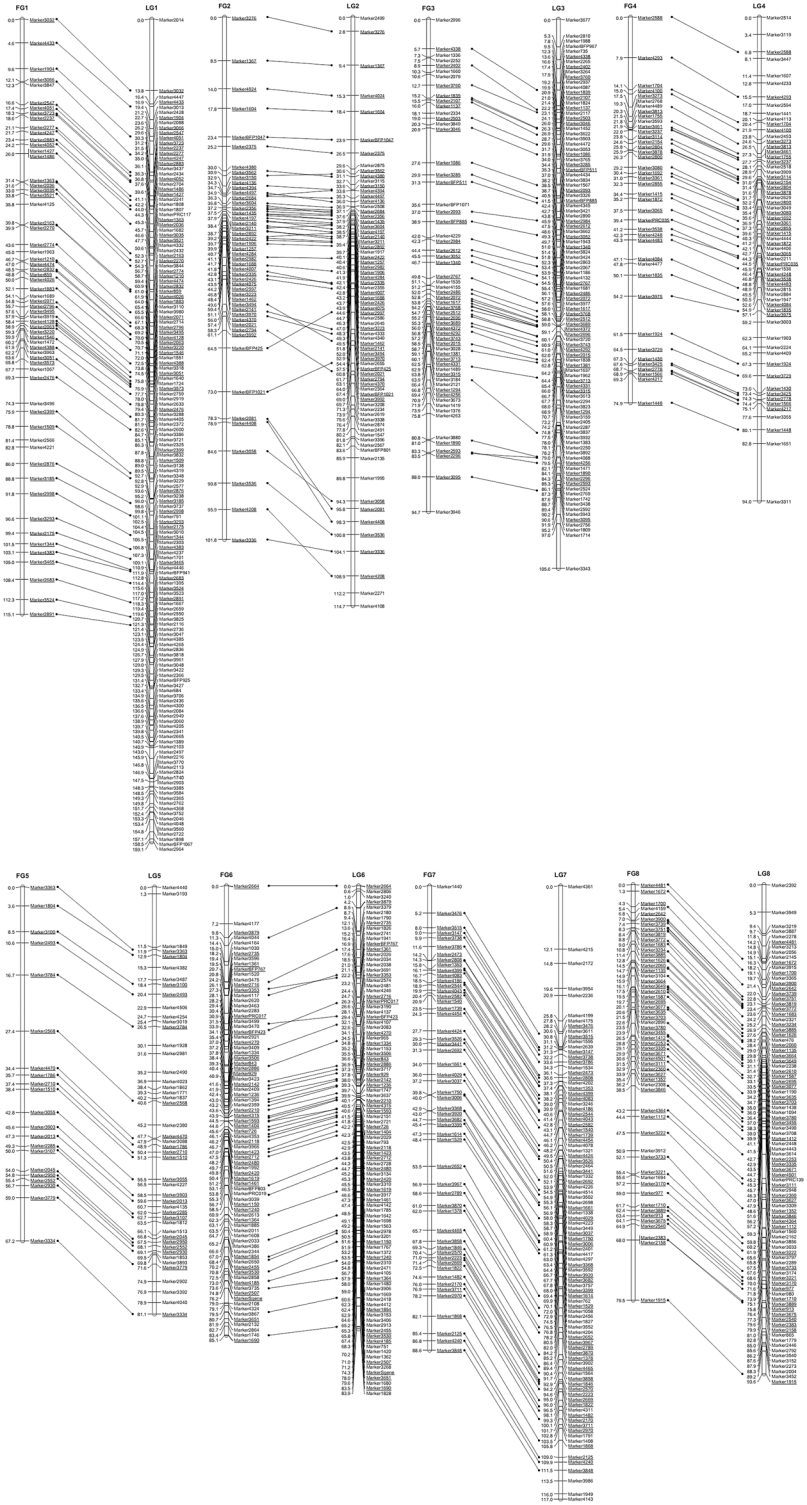
Collinearity between sweet cherry LGs derived the framework map (FG1-8) and the high density map (LG1-8). Sequence-anchored markers are indicated by connecting lines and are shown underlined. Inter-marker distance given in cM.

The frame map comprised 8 LGs (FG1-FG8) and was based on 409 markers was built (Fig 1). Its genetic length was 703.5 cM; the shortest LG was FG5 (67.2 cM) and the longest was FG1 (115.1 cM). The mean inter-marker interval was 1.86 cM (range from 1.18 cM on FG6 to 3.20 cM on FG5) (S4 Table). In all, 24 interval (5.87% of the total) were longer than 5.0 cM. The high density map included 309 additional markers, which increased the overall map length to 849.0 cM, while reducing the mean inter-marker distance to 1.18 cM and leaving just 10 (1.39%) marker intervals longer than 5.0 cM. Within each LG, the mean inter-marker interval ranged from 0.86 cM (LG6) to 1.73 cM (LG5) (Table 1). The incorporation of only a small number of the added markers perturbed the marker order predicted by the framework map, so that the extent of collinearity between the two maps was high (Fig 1). Once again, LG5 was the shortest LG and harbored the fewest loci, while LG1 was the longest and harbored the most loci. Segregation distortion affected 130 (18.57%) of the markers, and was noted on all LGs except for LG2; there were clusters of distorted markers on LG1 (123.5~159.1 cM), LG6 (1.1~24.4 cM and 54.0~58.0 cM) and LG7 (40.1~44.1 cM) (Table 1, S2 Fig). In total, 718 markers including 701 SLAFs, 16 SSRs and *S* gene, were mapped in the integrated ‘W×L’ map (Fig 1, S2 Fig).

**Table 1:** LG by LG breakdown of the markers contributing to the high density ‘W×L’ linkage map.

Linkage group	Marker number	Map length (cM)	Map density	> 5 cM marker distance	Sig < 0.05 marker	Ratio^a^	Added markers	Added map length
**LG1**	150	159.10	1.06	1	43	28.67%	88	44
**LG2**	74	114.70	1.55	3	0	0.00%	26	12.9
**LG3**	100	105.60	1.06	2	19	19.00%	40	10.9
**LG4**	63	94.00	1.49	1	10	15.87%	23	19.1
**LG5**	47	81.10	1.73	1	10	21.28%	26	13.9
**LG6**	98	83.90	0.86	0	25	25.51%	29	2.2
**LG7**	95	117.00	1.23	1	13	13.68%	42	28.4
**LG8**	91	93.60	1.03	1	10	10.99%	35	14.1
**Total**	718	849.00	1.18	10	130	18.11%	309	145.5

^a^ The ratio is No. of sig < 0.05 markers to total markers in each LG.

Of 203 SSR assays attempted, 28 (13.8%) were informative between the parental cultivars, 16 were assigned a map location and 14 were also represented on both the *Prunus* reference map [33], and sweet cherry ‘EF×NY’ map [5]. Matching LG assignment applied to 11 of the 14 common SSR loci (Table 2). The 3 exceptions were Prc117 (LG4 in peach, LG1 in sweet cherry) and Prc139 (LG1 in peach, LG8 in sweet cherry) and BPPCT009 (LG6 in peach, but mapping to LG2, LG4 and LG6 in sweet cherry). LG5 and LG7 were devoid of SSR loci.

**Table 2:** Conservation of SSRs between the sweet cherry ‘W×L’ map, other sweet cherry maps and the peach genome sequence.

‘W×L’ linkage goup	Marker name	SSR name	Peach genome	*Prunus* reference map	Sweet cherry ‘EF×NY’ map
**WL1**	BFP1067	BPPCT028	scaffold_1	G1	
**WL1**	BFP925	MA073a	scaffold_1	G1	-
**WL1**	Prc117	Prc117	scaffold_4	-	-
**WL1**	BFP941	UDAp-485	scaffold_1	G1	-
**WL2**	BFP1021	BPPCT009	scaffold_6	G4	NY6
**WL2**	BFP1047	UCD-CH36	-	-	-
**WL2**	BFP425	SC2	-	-	-
**WL2**	BFP801	UDAp-461	scaffold_2	G2	EF2
**WL3**	BFP967	BPPCT007	scaffold_3	G3	-
**WL3**	BFP885	EPDCU3083	scaffold_3	-	EF3
**WL3**	BFP511	BPPCT039	scaffold_3	G3	EF3
**WL4**	Prc035	Prc035	scaffold_4	-	-
**WL6**	BFP767	BPPCT008	scaffold_6	G6	EF6
**WL6**	Prc017	Prc017	scaffold_6	-	-
**WL6**	BFP423	SC1	scaffold_6	-	-
**WL8**	Prc139	Prc139	scaffold_1	-	-

The *S* locus mapped at 74.3 cM on LG6, flanked by the SLAF loci 3651 and 3268. The 3651 sequence shares homology with the region 27367312–27366944 bp of scaffold 6 in the peach genome, and the peach *S* locus in located between 26446961 and 26448303 bp on the same scaffold 6; thus the separation between the *S* locus and the sequence matching SLAF locus 3651 in peach was less 1 Mb.

### Synteny between the sweet cherry and peach genomes

**Fig 2:**
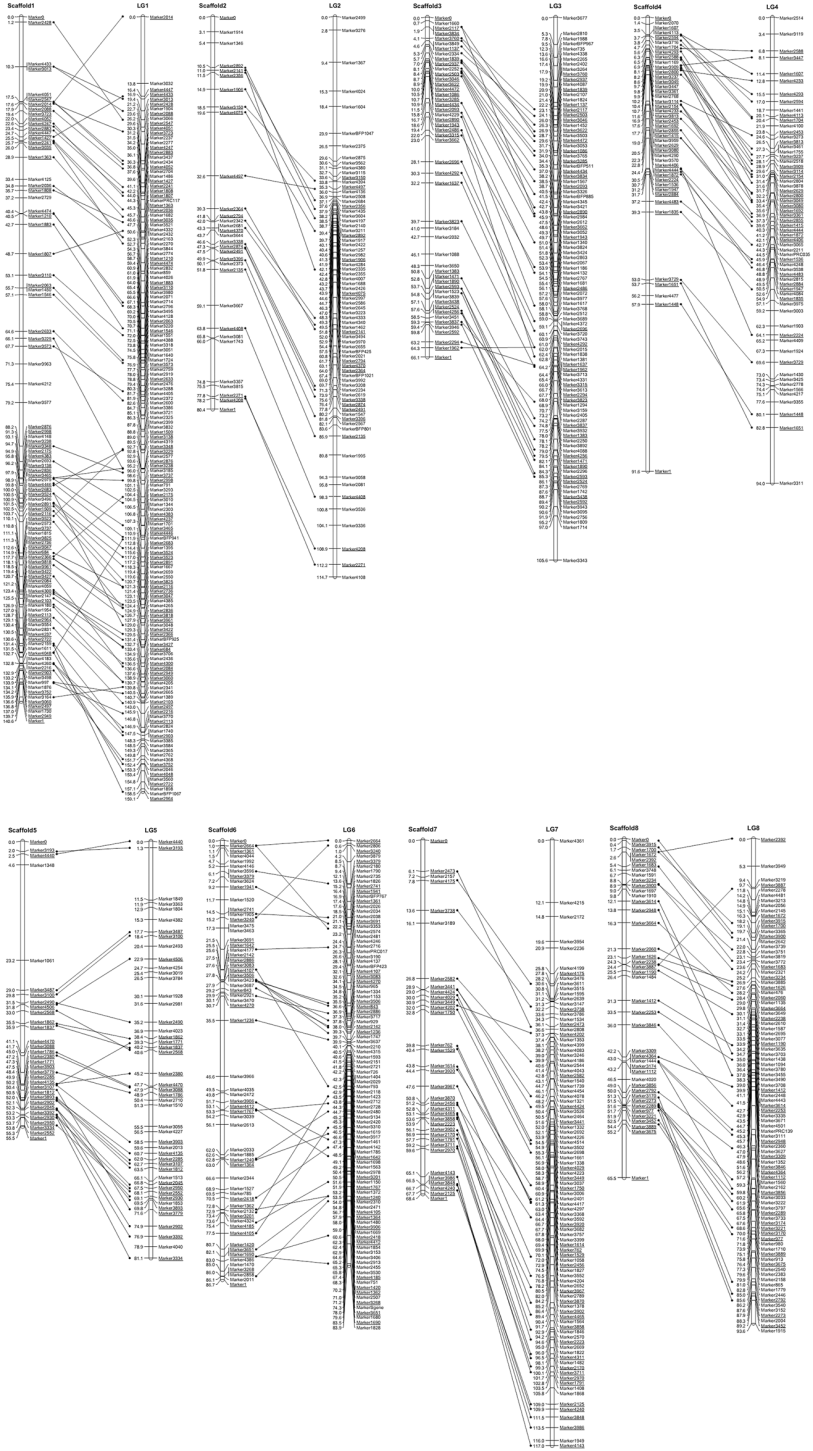
Collinearity between the sweet cherry high density map (LG1-8) and peach genome sequence (Scaffold1-8). In the latter, markers ‘0’ and ‘1’ refer to the two ends of each scaffold. Positions of anchored markers in each peach scaffold = physical position×3×10–6. Sequence-anchored markers are indicated by connecting lines and are shown underlined. Inter-marker distance given in cM.

Among the 953 SLAF sequences, 326 (34.2%) SLAFs shared at least 80% homology with a sequence represented in the peach genome; these were designated as similar sequences (SSs). In which, 272 SLAFs were mapped in the sweet cherry linkage map. Pearson correlation coefficient between the mapped location of these 272 SLAF loci and the location of the matching sequence in the peach genome sequence ranged from 0.904 to 0.979, indicative of a high level of synteny obtaining between the two genomes (Table 3, Fig 2). Regions of imperfect collinearity were observed on each LG: 0.4~8.2 Mb and 31.6~46.5 Mb of LG1, 3.5~10.8 Mb of LG2, 0.6 and 21.4 Mb of LG3, 2.1~5.6 Mb of LG4, 15.7~17.9 Mb of LG5, 0.3~5.0 Mb and 17.2~25.8 Mb of LG6, 2.03~10.7 Mb of LG7, 4.0~8.1 Mb of LG8 (Fig 2).

**Table 3:** LG by LG breakdown of SLAF sequences matching sequences present in the peach genome.

Linkage group	No. of similar sequence	No. of markers	Ratio of similar sequence to total marker	Pearson correlation (two-tails)
**LG1**	69	150	46.00%	0.979
**LG2**	18	74	24.32%	0.944
**LG3**	34	100	34.00%	0.923
**LG4**	34	63	53.97%	0.923
**LG5**	28	47	59.57%	0.930
**LG6**	25	98	25.51%	0.931
**LG7**	31	95	32.63%	0.964
**LG8**	32	91	35.16%	0.904
**Total**	271	718	37.74%	

### The detection of TD QTL

TD was continuous distributions, (S3 Fig) and increased year on year (S3 Fig). ATNG was highest between 2013 and 2014 (S3 Fig). Highly significant correlations were found among TDs from year to year over the period (r ranging from +0.60 to +0.93 across the full set of pairwise combinations). However, ATNG was not correlated in this way, presumably because this trait is so heavily determined by the environment. Correlations between TD and ATNG differed from year to year (Table 4). ATNG2012 was more correlated with TD2012~2014 (+0.62~0.65) than TD2011 (-0.16). ATNG2013 was more correlated with TD2013 and TD2014 (+0.58 and +0.52) than TD2012 (+0.36). ATNG2014 was most correlated with TD2014 (+0.50) and none correlated with TD2013.

**Table 4:** Year on year correlation for TD and ATNG.

Traits	TD2012	TD2013	TD2014	ATNG2012	ATNG2013	ATNG2014
**TD2011**	0.77**	0.75**	0.61**	0.16*	0.21	-0.16 *
**TD2012**		0.93**	0.78**	0.62**	0.36**	0.00
**TD2013**			0.83**	0.62**	0.58**	0.01
**TD2014**				0.65**	0.52**	0.50**
**ATNG2012**					0.34**	0.26*
**ATNG2013**						0.07

** Correlation is significant at the 0.01 level (2-tailed).

* Correlation is significant at the 0.05 level (2-tailed).

**Fig 3:**
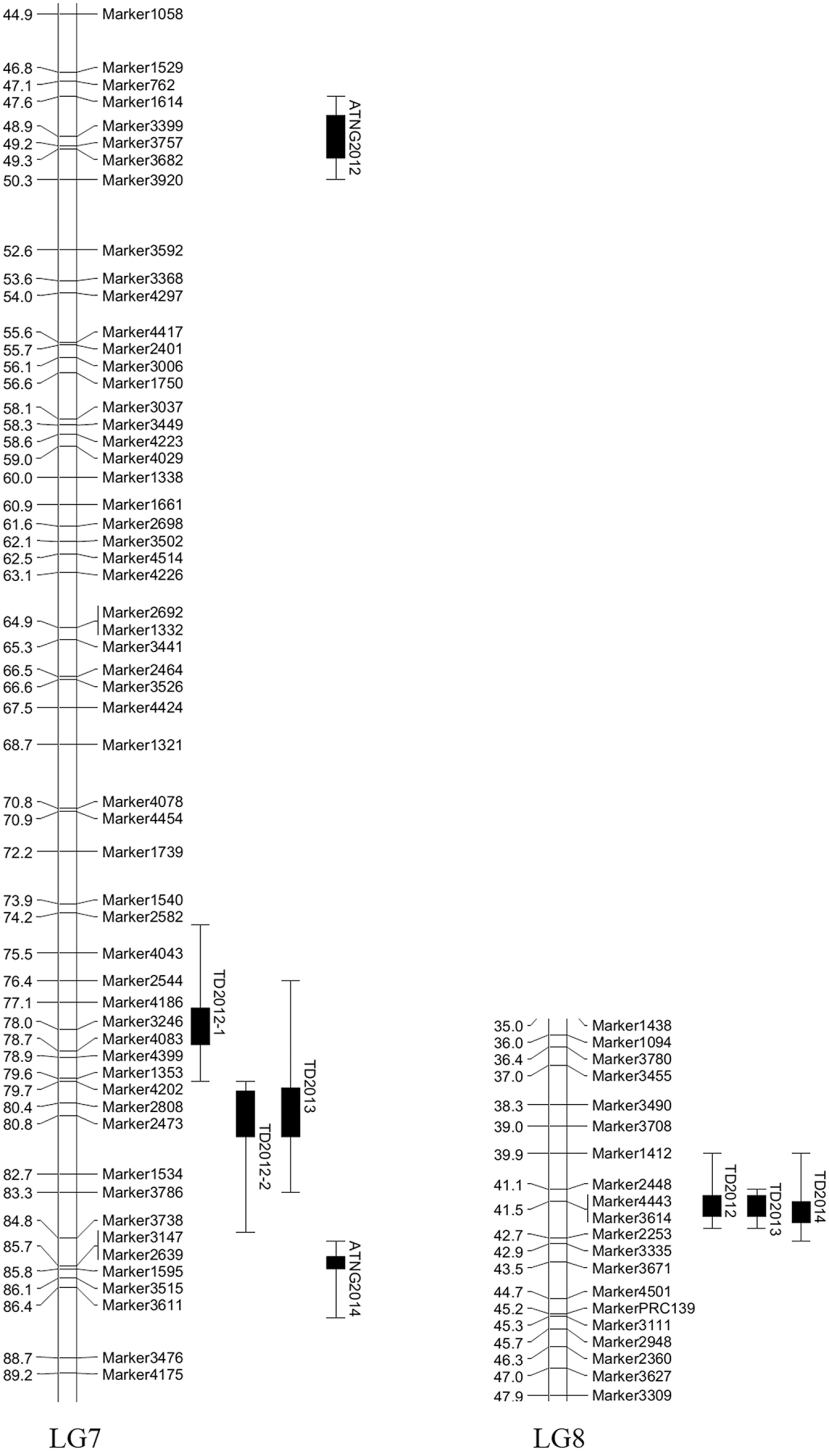
QTL for trunk diameter (TD) detected over the period 2012–2014 and annual trunk net growth (ATNG) in 2012 and 2014. 1-LOD and 2-LOD support intervals of each QTL are marked by thick and thin bars, respectively. The partial genetic map only includes the LGs harboring relevant QTL.

TD QTL was detected on 3 LGs. One mapped to LG6 but was only expressed in 2011. Two closely linked loci on LG7, one mapping in the region around 78.0 cM and the other around 80.4 cM (Table 5, Fig 3). The former explained 15.7% of the phenotypic variance for TD in 2012 and 15.1% in 2013; the latter explained 21.1% and 20.1%, respectively. The final locus mapped to LG8 in the region of 41.5 cM; this locus was expressed in 2012, 2013 and 2014, explaining, respectively, 21.5%, 21.5%, and 16.8% of the variance for TD. For the trait ATNG2012 and ATNG2014, QTL were mapped to different regions of LG7 (respectively, 49.2 cM and 85.7 cM) while for ATNG2013, no QTL was detected. The site of the ATNG2014 QTL was closed to a TD QTL. About 30% of the mapping population trees first bore fruit in 2012, while above 90% of them bore fruit in 2013. These results revealed that different QTLs control TD in different tree development stage, those on LG7 and LG8 mainly controlled TD after fruit.

**Table 5:** TD and ATNG QTL identified in the ‘W×L’ mapping population.

Trait	Year	Linkage group	LOD	Position	Marker	Expl%
**TD**	2011	LG6	3.04	28.1	Marker4137	18.10%
	2012	LG7	3.24	78.0	Marker3246	15.70%
			3.61	80.4	Marker2808	21.10%
			3.29	80.8	Marker2473	17.30%
		LG8	4.28	41.5	Marker3614	21.50%
	2013	LG7	3.61	80.4	Marker2808	20.10%
			3.29	80.8	Marker2473	17.50%
			2.97	78.0	Marker3246	15.10%
		LG8	4.11	41.5	Marker3614	21.50%
	2014	LG8	3.01	41.5	Marker3614	16.80%
**ATNG**	2012	LG7	3.67	48.9	Marker3399	23.50%
		LG7	3.24	49.2	Marker3757	21.50%
	2014	LG7	3.02	85.7	Marker2639	16.20%

Determining the favorable QTL alleles, identified by their flanking markers, is critical for further QTL validation and eventual utilization in MAS. A haplotype analysis was conducted featuring the alleles present at the two LG7 SLAF marker2808 and marker2473 and the LG8 SLAF marker3614 (Table 5). Mapping population trees of genotype marker2808 ‘*lm*’, 2473 ‘*nn*’ and 3614 ‘*lm*’ produced the largest TD over the period 2012–2014 (Table 6). All three favorable alleles were inherited from ‘Lapins’. The only sequence hit obtained from a Blast-based search using the three marker sequences as query was for 3614, which almost completely matched a sequence present in an exon of GIGANTEA in almond (KJ502316.1).

**Table 6:** Mean TD values of mapping population progeny classified according to marker haplotype in loci linked to 3 TD QTL.

Linkage group	Marker	Allele	SNP	Average trait value		
				2012	2013	2014
**LG7**	Marker2808	ll	G	49.40	56.44	70.23
		lm	G/C	52.34	60.44	77.12
**LG7**	Marker2473	nn	T	53.31	60.46	73.98
		np	T/C	48.65	55.99	73.78
**LG8**	Marker3614	ll	C	46.87	54.10	69.40
		lm	T/C	59.62	68.51	82.41

## Discussion

Linkage between a conveniently assayable marker and a gene of breeding value is the foundation of marker assisted breeding [34]. Establishing such linkages for one or more QTL requires the elaboration of a genome-wide linkage map, which in turn requires that a substantial number of informative markers be generated and that a suitable mapping population be constructed. Here, the aim was to rapidly generate a high density sweet cherry genetic linkage map, and the SLAF-seq strategy proved to be most effective in both producing the necessary markers and in performing the genotyping. Sequencing depth is an important consideration with respect to the quality of a SLAF-based linkage map. It has been suggested that sequencing depth over 4× had relatively little influence on sequencing error rates [21]. In this paper, the sequence depth was 5.38× for progenies and above 20× for parents. The 20× sequence depth for parents could ensure the sequence correction of SLAFs mapped in linkage map. The 5.38× sequence depth for progenies could ensure the correction in genotyping.

The current ‘W×L’ sweet cherry linkage map compares well with already established ones. Its length (849 cM) lies in the same ball park as that of both the ‘BT×K’ map (752.9 cM) [8] and a *Prunus* consensus map built from four populations (779.4 cM) [7]. With respect to the individual LGs, LG1 is consistently the longest and LG5 the shortest, while the S gene in each case has been mapped to one end of LG6 [5–8]. About 18.57% of the markers represented in the ‘W×L’ sweet cherry linkage map suffered from significant segregation distortion, and there was evidence of certain hot spots of distortion on 123.5~159.1 cM of LG1 and 1.1~24.4 cM of LG6, regions which also have been noted to be liable to this phenomenon in ‘PA×PN’ [6] and ‘Lapins’ [8] genetic background.

Creating a linkage map in a highly heterozygous species such as sweet cherry is less straight-forward than in a species where the mapping parents are highly homozygous; the heterozygosity of the parents means that a larger number of gametic types will be generated. A possible strategy used in some cases was first to construct two separate parental maps, and then to combine them using common markers to produce an integrated map [8,9]. However, a problem would appear if homozygous hk×hk pattern marker existed in one group. It would be difficult to combine the parental map and reduce the information into an integrated map. Here, an alternative strategy was pursued, in which a set of high-quality markers was initially used to build a framework map first, and subsequently the remaining markers were added; this latter step caused very little perturbation to mark order, so that the level of collinearity between the framework map and high-density map was very high.

DNA sequence information of the SLAF markers facilitated a cross-species comparison of the linkage map with the peach genome. About 34.2% of the SLAF markers were homologous with peach sequences, which defined a substantial number of sequence-anchored marker position and confirmed the suggestion made elsewhere [8, 27] that these two *Prunus* species are highly syntenous. Synteny extended to the *S* locus, since in sweet cherry, the SLAF marker (3165) most closely linked to the gene harbored a sequence which is highly homologous to a peach sequence lying within 1 Mb of the peach *S* locus.

TD is the two-way channel for nutrients transportation between root and blade, which reflects the overall condition of root biomass, foliage mass, root efficiency and leaf quantity [35]. TD has been documented to exert a direct influence on yield and fruit quality [35, 28]; unlike tree shape, it is not readily manipulable by pruning. Until now, the genetic determinism of TD has hardly been addressed in sweet cherry [36]; although, some effort in this direction has been invested both in apricot (*Prunus armeniaca*) [37] and mei (*Prunus mume*) [38]. In the former case, TD QTL have been mapped to both LG1 and LG2 [37], while in the latter, the major site was on LG8 [38]. Here, the indications were that in sweet cherry, the important TD QTL are sited on LG6, LG7 and LG8, which is a partly similar result with that of mei.

The trait proved to be highly correlated across years, which was not unexpected given that the TD measured in any particular year represents the tree’s accumulated growth over the preceding years. In contrast, the ATNG trait was poorly amenable to genetic analysis because it is strongly mediated by the growing environment. So, TD is a more stable tree character than ATNG. The results, QTLs of TD were stabled in 78~80 cM of LG7 and 41.5 cM of LG8, and QTLs of ATNG in 2012 and 2014 were located in different position of LG7, were consistent with traits relationship. ATNGs show more correlations with this year TD than last TD. In corresponding, QTLs for ATNG2012 and TD2012 were located in LG7 while QTL of TD2011 was in LG6. The various TD QTL was expressed at a different stage of the tree’s development. Thus, once the trees had started to produce fruit, TD was more strongly affected by the LG7 and LG8 QTL and not at all by the LG6 locus. A similar transition in genetic control has been noted for *Populus* sp. [39, 40].

The LG8 TD QTL was responsible for some 20% of the phenotypic variation once the trees were old enough to set fruit. Its most closely linked marker (SLAF 3614) harbored sequence present in almond GIGANTEA, a gene which in *A*. *thaliana* regulates a number of developmental processes [41, 42, 43]; its expression has also been correlated with fruit set [44] and the regulation of wall in-growth deposition in phloem parenchyma transfer cells [45]. Both these two latter activities are in keeping with the major function of the tree trunk, which provides the physical connection between the plant's root system and it photosynthetic apparatus.

The ‘W×L’ progeny varied not just for TD, but also for a number of significant fruit characters. The high density linkage map offers a straight-forward means of determining the genetic basis of this phenotypic variation, thereby opening the way to accelerating sweet cherry improvement by exploiting marker assisted selection.

## Supporting Information

S1 Fig The prediction chromosomal distribution of SLAFs across the 8 peach chromosomes. Eight scaffolds correspond to the eight peach chromosomes. The color (yellow to dark) indicated the number (0 to 10) of predicted SLAFs attributed in 8 peach chromosomes.
(TIF)

S2 Fig The high density genetic linkage map of sweet cherry derived from the ‘W×L’ population. Inter-marker distance given in cM. Loci exhibiting skewed distribution are marked by asterisks to indicate distortion level (* for p<0.1; **p<0.05; ***p<0.01; **** p<0.005; ***** p<0.001; ****** p<0.0005; ******* p<0.0001).
(TIF)

S3 Fig Distribution of trunk diameter (TD) and anural trunk net growth (ATNG) measured for 4 years. (TIF)

S1 Table Sweet cherry genetic linkage map recently reported. (DOCX)

S2 Table Ratios of polymorphism SLAF, non-polymorphism SLAF and repeat sequence in the total high quality SLAF. (DOCX)

S3 Table Segregation pattern of the 982 polymorphism markers. (DOCX)

S4 Table The number of marker, map length and density of the framework linkage map derived from the ‘W×L’ population. (DOCX)

S5 Table Sequences and segregation patterns of the 701 SLAF loci included in the high density sweet cherry map. (DOCX)
