# Supplementary figures and images for: Construction of Commercial Sweet Cherry Linkage Maps and QTL Analysis for Trunk Diameter
Source: PLoS One. 2015 Oct 30;10(10):e0141261. doi: 10.1371/journal.pone.0141261 (PMC4627659; doi:10.1371/journal.pone.0141261)

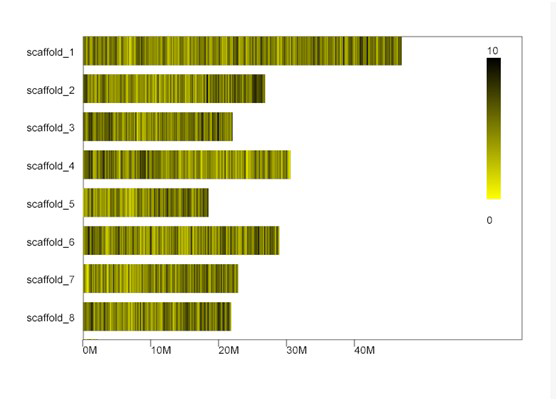

Supplement: S1 Fig — Eight scaffolds correspond to the eight peach chromosomes. The color (yellow to dark) indicated the number (0 to 10) of predicted SLAFs attributed in 8 peach chromosomes. (TIF) [file pone.0141261.s001.tif]

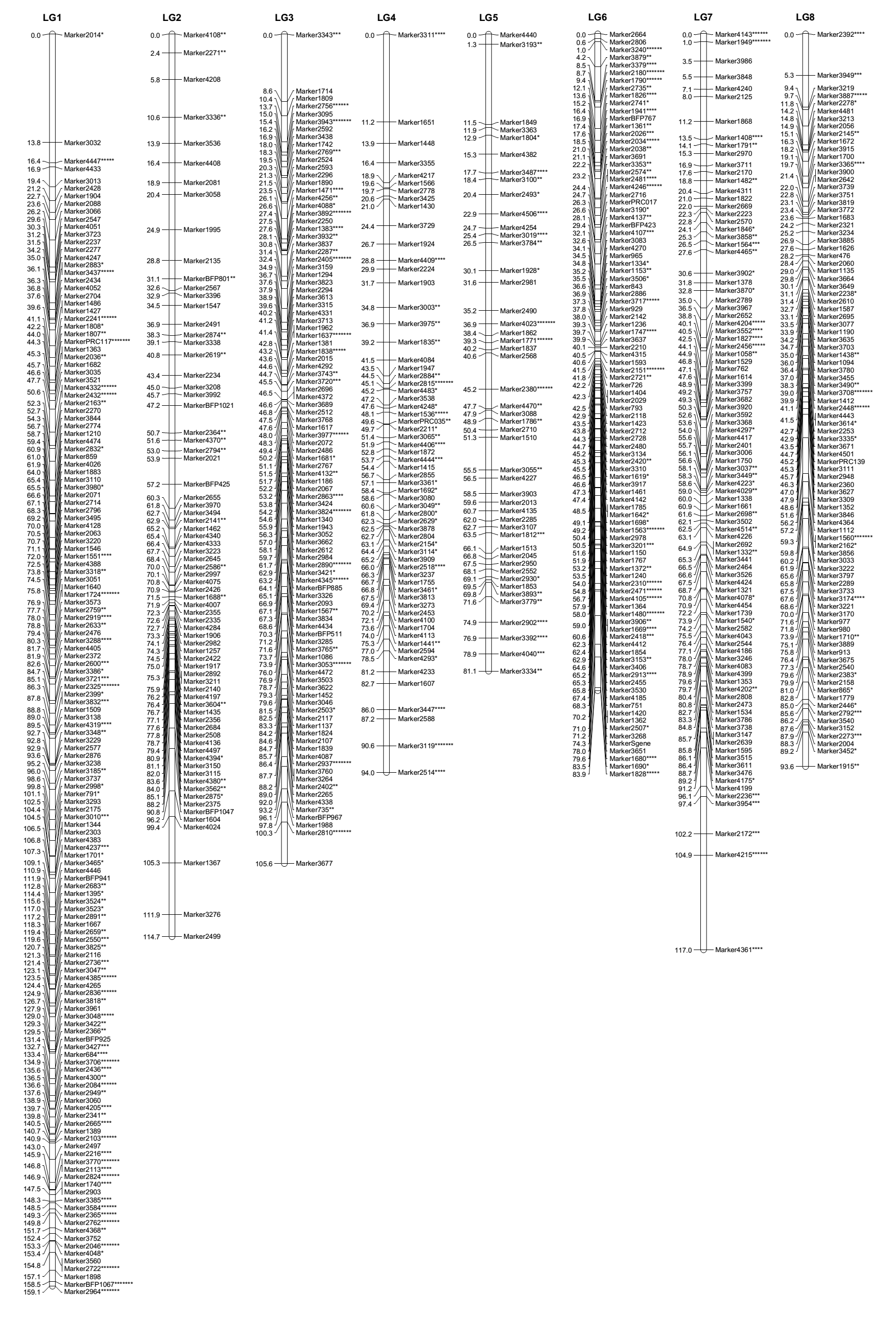

Supplement: S2 Fig — Inter-marker distance given in cM. Loci exhibiting skewed distribution are marked by asterisks to indicate distortion level (* for p<0.1; **p<0.05; ***p<0.01; **** p<0.005; ***** p<0.001; ****** p<0.0005; ******* p<0.0001). (TIF) [file pone.0141261.s002.tif]

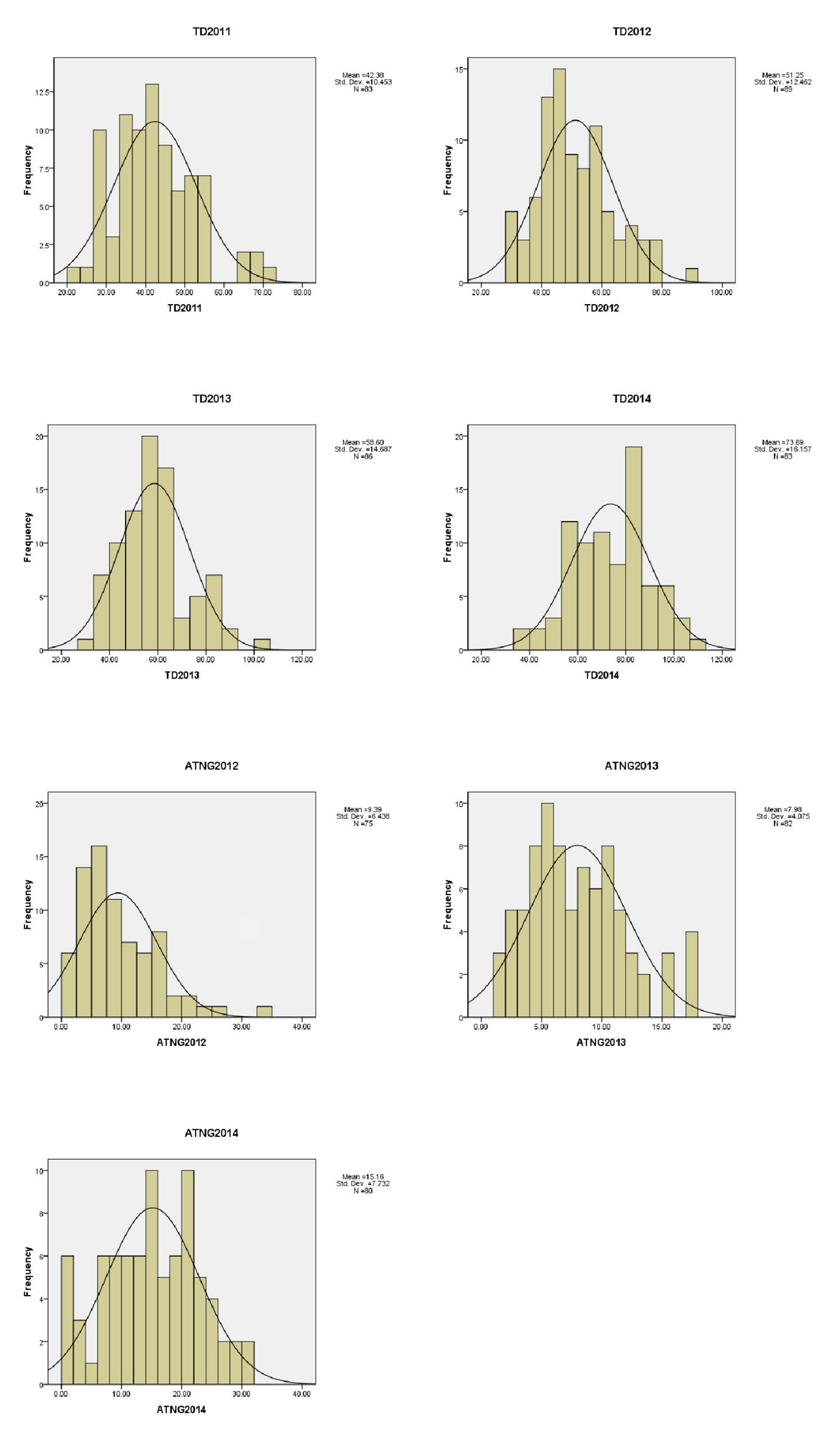

Supplement: S3 Fig — (TIF) [file pone.0141261.s003.tif]
