# Supplementary material for: Construction of Commercial Sweet Cherry Linkage Maps and QTL Analysis for Trunk Diameter
Source: PLoS One. 2015 Oct 30;10(10):e0141261. doi: 10.1371/journal.pone.0141261 (PMC4627659; doi:10.1371/journal.pone.0141261)
Supplement: S1 Table — (DOCX) [file pone.0141261.s004.docx]

**S1 Table. Sweet cherry genetic linkage map recently reported**

| Constructed  year | Species used for map | Total map length (cM) | Average marker density (cM) | Main marker types used in mapping | Reference |
| --- | --- | --- | --- | --- | --- |
| 2008 | *Prunus* *avium*×wild forest cherry | 711.2/565.8 | 4.94/6.22 | SSR | [5] |
| 2009 | *Prunus avium*×*Prunus nipponica* | 736 | 3.16 | SSR/Isozyme | [6] |
| 2012 | *Prunus avium*  (Intraspecific/interspecific) | 779.4 | 5.4 | SNP/SSR | [7] |
| 2013 | *Prunus avium* | 752.9/639.9 | 1.1/0.9 | SNP | [8] |
| 2015 | *Prunus avium* | 731.3 | 0.7 | SNP/SSR | [32] |
