# Supplementary material for: Construction of Commercial Sweet Cherry Linkage Maps and QTL Analysis for Trunk Diameter
Source: PLoS One. 2015 Oct 30;10(10):e0141261. doi: 10.1371/journal.pone.0141261 (PMC4627659; doi:10.1371/journal.pone.0141261)
Supplement: S2 Table — (DOCX) [file pone.0141261.s005.docx]

**S2 Table.** **Ratios of polymorphic SLAF, non-polymorphic SLAF and repeat sequence in the total high quality SLAF**

| Type | Number | Percentage |
| --- | --- | --- |
| Total high quality SLAFs | 14,634 | 100% |
| Polymorphic SLAFs | 1,838 | 12.56% |
| Repeat sequence | 727 | 4.97% |
| Non-polymorphic SLAFs | 12,069 | 82.47% |
