# Supplementary material for: Construction of Commercial Sweet Cherry Linkage Maps and QTL Analysis for Trunk Diameter
Source: PLoS One. 2015 Oct 30;10(10):e0141261. doi: 10.1371/journal.pone.0141261 (PMC4627659; doi:10.1371/journal.pone.0141261)
Supplement: S3 Table — (DOCX) [file pone.0141261.s006.docx]

**S3 Table. Allelic classes** **of the 982 informative loci used in the sweet cherry linkage map construction**

| Segregation pattern^a^ | SLAF | SSR | S gene | Total | Ratio of each pattern |
| --- | --- | --- | --- | --- | --- |
| ab×cd | 1 |  | 1 | 2 | 0.20% |
| ef×eg | 66 |  |  | 66 | 6.72% |
| hk×hk | 95 | 5 |  | 100 | 10.18% |
| lm×ll | 514 | 10 |  | 524 | 53.36% |
| nn×np | 277 | 13 |  | 290 | 29.53% |
| Total | 953 | 28 | 1 | 982 |  |

a: The first parent is ‘Lapins’ and the second is ‘Wanhongzhu’ in each segregation pattern.
