# Supplementary material for: Construction of Commercial Sweet Cherry Linkage Maps and QTL Analysis for Trunk Diameter
Source: PLoS One. 2015 Oct 30;10(10):e0141261. doi: 10.1371/journal.pone.0141261 (PMC4627659; doi:10.1371/journal.pone.0141261)
Supplement: S4 Table — (DOCX) [file pone.0141261.s007.docx]

**S4 Table.** **The number of marker, map length and density of the framework linkage map derived from the ‘W×L’ population**

| Group | No. of marker | Map length(cM) | Marker density | >5 cM interval |
| --- | --- | --- | --- | --- |
| FG1 | 62 | 115.1 | 1.86 | 1 |
| FG2 | 48 | 101.8 | 2.12 | 9 |
| FG3 | 60 | 94.7 | 1.58 | 3 |
| FG4 | 40 | 74.9 | 1.87 | 4 |
| FG5 | 21 | 67.2 | 3.20 | 4 |
| FG6 | 69 | 81.7 | 1.18 | 1 |
| FG7 | 53 | 88.6 | 1.67 | 1 |
| FG8 | 56 | 79.5 | 1.42 | 1 |
| Total | 409 | 703.5 | 1.86 | 24 |
