# Supplementary material for: Construction of Commercial Sweet Cherry Linkage Maps and QTL Analysis for Trunk Diameter
Source: PLoS One. 2015 Oct 30;10(10):e0141261. doi: 10.1371/journal.pone.0141261 (PMC4627659; doi:10.1371/journal.pone.0141261)
Supplement: S5 Table — (DOCX) [file pone.0141261.s008.docx]

**S5 Table. Sequences and segregation of the 701 SLAF loci in the high density sweet cherry map**

| SLAF | Sequence | Segregation |
| --- | --- | --- |
| Marker1058 | AATTCATACTCTTAGGTTTGGAGATAAAATGCTACACTAAXXXXXXXXXXAAAAGTGTACCTTATTCATCGGCTGCCATCCTTCTTGCCA | efxeg |
| Marker1058 | ATTCATACTCTTAGGTTTGGAGATAAAATGCTACACTAAAXXXXXXXXXXAAAAGTGTACCTTATTCATCGGCTGCCATCCTTCTTGCCA | efxeg |
| Marker1058 | ATTCATACTCGTAGGTTTGGAGATAAAATGCTACACTAAAXXXXXXXXXXAAAAGTGTACCTTATTCATCGGCTGCCATCCTTCTTGCCA | efxeg |
| Marker1086 | TACAGAAATAAATTGAAAATGATATTGGTTTTGTTGTTGGXXXXXXXXXXCCAATTTTCTTTGACCAATTTGCCATCATTCCATCATCCA | efxeg |
| Marker1086 | TACAGAAATAAATTGAAAATGATATTGGTTTTGTTGTTGGXXXXXXXXXXCCAATTTTCTTTGACCAATTTCCCATCATTCCATCATCCA | efxeg |
| Marker1086 | TACAGAAATAAATTGAAAATGATATTGGTTTTGTTGTTGGXXXXXXXXXXCCAATTTTCTTTGACCAATTTACCATCATTCCATCATCCA | efxeg |
| Marker1094 | TTTTCATTTTACTCGAAATTACAAATTTACCATTAGTATTXXXXXXXXXXTTGTCTTATAACGAAGAAGAGGCATCACCCATAGTATTTG | efxeg |
| Marker1094 | TTTTCATTTTACTCGAAATTACAAATTTACCATTAGTATTXXXXXXXXXXTTGTCTTATAATGAAGAAGAGGCATCACCCATAGTATTTG | efxeg |
| Marker1094 | TTTTCATTTTACTCGAAATTACAAATTTACCATTAGTATTXXXXXXXXXXTTGTCTTATAACGAAGAAGAGGCATCACCCATAGTAGTTG | efxeg |
| Marker1112 | CCCATTATTTCTAAACAACCCATATCCCTTCACCTTCACTXXXXXXXXXXAGGTCCAACTAATTGTCAAGTAAATCCAGCCCAACTAATT | efxeg |
| Marker1112 | CCCATTATTTCTAAACAACCCATATCCCTTCACCTTCACGXXXXXXXXXXAGGTCCAACTAATTGTCAAGTAAATCCAGCCCAACTAATT | efxeg |
| Marker1112 | CCCATTATTTCTAAACAACCCATATATCCCTTCACCTTCAXXXXXXXXXXAGGTCCAACTAATTGTCAAGTAAATCCAGCCCAACTAATT | efxeg |
| Marker1135 | AAATAACCCAAACAAACTCAGCAATCTCTTCATATTCTTCXXXXXXXXXXAGGGTGAGTGATAGGATTTTTAGTACCTGCGCAAACATGG | efxeg |
| Marker1135 | AAAATAACCCAAACAAACTCAGCAATCTCTTCCTATTCTTXXXXXXXXXXAGGGTGAGTGATAGGATTTTTAGTACCTGCGCAAACATGG | efxeg |
| Marker1135 | AAAATAACCCAAACAAACTCAGCAATCTCTTCATATTCTTXXXXXXXXXXAGGGTGAGTGATAGGATTTTTAGTACCTGCGCAAACATGG | efxeg |
| Marker1137 | GCGTGTGTCCAGTATTTCAAGCCTTTCAAGTTATCTAACTXXXXXXXXXXAAAGAGCTTACAATGAAAAATGAAGTTTACACCGCTGGAG | efxeg |
| Marker1137 | GCGTGTGTCCGGTATTTCAAGCCTTTCAAGTTATCTAACTXXXXXXXXXXAAAGAGCTTACAATGAAAAATGAAGTTTACACCGCTGGAG | efxeg |
| Marker1137 | GCGTGTGTCCGGTATTTCAAGCCTTTCAAGTTATCTAACTXXXXXXXXXXAAAGAGCTTACAATGAAAATTGAAGTTTACACCGCTGGAG | efxeg |
| Marker1150 | TTGTGGGCTCGAAACCCAACATGTGATACGTATGCAAAAGXXXXXXXXXXCTCCCGAATACCTCATGAGTAGAGTTGTTTGGATAGTTTT | efxeg |
| Marker1150 | TTGTGGGCTCGAAACCCAACATGTGATACATATCCAAAAGXXXXXXXXXXCTCCCGAATACCTCATGAGTAGAGTTGTTTGGATAGTTTT | efxeg |
| Marker1150 | TTGTGGGCTCGAAACCCAACATGTGATACGTATGCAAAAGXXXXXXXXXXCTCCCGAATACCTCATGAGTAGAGTTGTTTGGATAGGTTT | efxeg |
| Marker1153 | AAATACAAAATCCAAAGTTAGGGGTAAGCTTCTCTATGTGXXXXXXXXXXGCGGAAAACCTTTATTTTGGCACCAAGCCACGCTACAAGC | efxeg |
| Marker1153 | AAATACAAAATCCAAAGTTAGGGGTAAGCTTCTCTATGTGXXXXXXXXXXGCAGAAAACCTTTATTTTGGTACCAAGCCACGCTACAAGC | efxeg |
| Marker1153 | AAATACAAAATCCAAAGTTAGGGGTAAGCTTCTCTATGTGXXXXXXXXXXGCGGAAAACCTTTATTTTGGTACCAAGCCACGCTACAAGC | efxeg |
| Marker1186 | CCGAATTCACAGGGTTTCTGTGTTCTTAGCCGTGTTCACAXXXXXXXXXXCTTATCCCGAAACTCTAAACACATTTTTTTTCTCTTATTC | nnxnp |
| Marker1186 | CCGAATTCACAGGGTTTCTGTGTTCTTAGCCGTGTTTACAXXXXXXXXXXTTATCCCGAAACTCTAAACACATTTTTTTTTCTCTTATTC | nnxnp |
| Marker1186 | CCGAATTCACAGGGTTTCTGTGTTCTTAGCCGTGTTCACAXXXXXXXXXXTTATCCCGAAACTCTAAACACATTTTTTTTTCTCTTATTC | nnxnp |
| Marker1190 | TGCATAAATCTTTGCTAAATAAGAAATAGTCATATTTGTGXXXXXXXXXXAAAGCAATGTAAGGACAAACTAAATGGAACCATTGCCAAC | lmxll |
| Marker1190 | TGCATAAATCTTTGCTAAATAAGAAATAGTCATATTTGTGXXXXXXXXXXAAAGCAATGGAAGGACAAACTAAATGGAACCATTGCCAAC | lmxll |
| Marker1190 | TGCGTAAATCTTTGCTAAATAAGAAATAGTCATATTTGTGXXXXXXXXXXAAAGCAATGGAAGGACAAACTAAATGGAACCATTGCCAAC | lmxll |
| Marker1210 | TTTCGTCGCTTTGAATGACTCAGATTGTTTATTGTTGACCXXXXXXXXXXTTCGTGCTGTCCAGGTAAGAGTTGGGGTTACGTCCACATT | efxeg |
| Marker1210 | TTTCGTCGCTTTGAATGACTCGGATTGTTTATTGTTGACCXXXXXXXXXXTTCGTGCTGTCCAGGTAAGAGTTGGGGTTACGTCCACATT | efxeg |
| Marker1210 | TTTCGTCGCTTTGAATGACTCGGATTGTTTATTGTTGACCXXXXXXXXXXTTCATGCTGTCCAGGTAAGAGTTGGGGTTACGTCCACATT | efxeg |
| Marker1236 | ACCCAATCCCTTCCACATGCCTGAAAGGAACCTCATCAATXXXXXXXXXXTATAGGACTAAGACTTTCATTTAGGTTGGTGAGAGATGTG | efxeg |
| Marker1236 | ACCCAATCCCTTCCACATGCCTGAAAGGAAGCTCATCAATXXXXXXXXXXTATAGGACTAAGACTTTCATTTAGGTTGGTGAGAGATGTG | efxeg |
| Marker1236 | ACCCAATCCCTTCCACATGCCTGAAAGGAAGTTCATCAATXXXXXXXXXXTATAGGACTAAGACTTTCATTTAGGTTGGTGAGAGATGTG | efxeg |
| Marker1240 | TATCAATTGCAAATTGCGAGAGATGCTCCAAAATGAGATCXXXXXXXXXXATACTTTTGAACACAAGTAGCATTAGCATGAGTTATTAGA | efxeg |
| Marker1240 | TATCAATTGCAAATTGCGAGAGATGTTCCAAAATGAGATCXXXXXXXXXXATACTTTTGAACACAAGTAGCATTAGCATGAGTTATTAGA | efxeg |
| Marker1240 | TATCAATTGCAAATTGTGAGAGATGCTCCAAAATGAGATCXXXXXXXXXXATACTTTTGAACACAAGTAGCATTAGCATGAGTTATTAGA | efxeg |
| Marker1257 | GTAAGAACATTCAATCAAACGACACAAGAGATTACGTGGTXXXXXXXXXXATGAGGGAAAACAACTCGAAATTTAGCTAAAAGTTGGGAA | efxeg |
| Marker1257 | GTAAGAACATTCAATCAAGCGACACAAGAGATTACGTGGTXXXXXXXXXXATGAGGGAAAACAACTCGAAATTTAGCTAAAAGTTGGGAA | efxeg |
| Marker1257 | GTAAGAACATTCAATCAAGCGACACAAGAGATTACGTAGTXXXXXXXXXXATGAGGGAAAACAACTCGAAATTTAGCTAAAAGTTGGGAA | efxeg |
| Marker1294 | AAAATGGGGAAAGAAAACAGACTTCGAAATAAATTTCTTCXXXXXXXXXXAAGAAATATGTTGAGAGAAAATAATATAAACAAACTTCAA | efxeg |
| Marker1294 | AAAAGGGGGAAAGAAAACAGACTTCGAAATAAATTTCTTCXXXXXXXXXXAAGAAATATGTTGAGAGAAAATAATATAAACAAACTTCAA | efxeg |
| Marker1294 | AAAATGGGGAAAGAAAATAGACTTCGAAATAAATTTCTTCXXXXXXXXXXAAGAAATATGTTGAGAGAAAATAATATAAACAAACTTCAA | efxeg |
| Marker1321 | AAAAAAAACTAACTATTTAGTTTATACTCTCGCTATATTCXXXXXXXXXXAAAAAAAAATCAAATAAATTGTAAGATTACAAAGTCGAGC | efxeg |
| Marker1321 | AAAAAAAACTAACTATTTAGTTTATACTCTTGCTATATTCXXXXXXXXXXAAAAAAAAATCAAATAAATTGTAAGATTACAAAGTCGAGC | efxeg |
| Marker1321 | AAAAAAAAACTAACTATTTAGTTTATACTCTCGCTATATTXXXXXXXXXXAAAAAAAAATCAAATAAATTGTAAGATTACAAAGTCGAGC | efxeg |
| Marker1332 | CATTGTCATGTCACACAAATTCCTTTGCGATTGATGAAGTXXXXXXXXXXATTGGTAGAGGTATGAAGTTAGTGAAGTGTGTTCCGTAGG | nnxnp |
| Marker1332 | CATTGTCATGTCACACAAATTCCTTTGCGATTGATGAAGTXXXXXXXXXXATTGGTAGAGGTATGAAGTTAGTGAAGTGTGTTCCGTAGA | nnxnp |
| Marker1334 | AATGACAACAAACAAAGTTTTTCCGCTGTTGATGACATCAXXXXXXXXXXTTATAACAAAAGTCAGAAGCATTTTCTGTTTTCAAGCAAT | nnxnp |
| Marker1334 | AATGACAACAAACAAAGTTTTTCCGCTGTTGATGACATCAXXXXXXXXXXTTAGAACAAAAGTCAGAAGCATTTTCTGTTTTCAAGCAAT | nnxnp |
| Marker1338 | TATTTAGGCATGCAATTACCAAATTGTAAGGGGATTTTTAXXXXXXXXXXATATCTTCACAAAAAGAACTGATAAGCCTTATATCCTGGA | lmxll |
| Marker1338 | TATTTAGGCATGCAATTACCAAATCGTAAGGGGATTTTTAXXXXXXXXXXATATCTTCACAAAAAGAACTGATAAGCCTTATATCCTGGA | lmxll |
| Marker1340 | GCGTTTATTTGTTATTGGTGTAGATCATATAGACCGCATCXXXXXXXXXXTGCTATCTATATGTTATCTAAGAATGTTACTTGGTTTTGG | nnxnp |
| Marker1340 | GCGTTTATTTGTTATTGGTGTAGAACCTATAGACCGCATCXXXXXXXXXXTGCTATCTATATGTTATCTAAGAATGTTACTTGGTTTTGG | nnxnp |
| Marker1344 | GTAAGTTCGGGAATAGAAAGTTCAACTGACAGATCAATAGXXXXXXXXXXAAGTAAAGTCAAATTTACAACTTTACACAGTGTATGTTCT | lmxll |
| Marker1344 | GTAAGTTCGGGAATAGAAAGTTCAACTGACAGATCAATAGXXXXXXXXXXAAGTAAAGTCAAATTTACAACTTTACACAATGTATGTTCT | lmxll |
| Marker1352 | CCAAGCACAAATTACACAAGTGTTTATAGAGGTTCAGCAAXXXXXXXXXXAGCTTGTCCTCCACGTTCTTCCCTTTAGTCACCACTTAGA | lmxll |
| Marker1352 | CCAAGCACAAATGACACAAGTGTTTATAGAGGTTCAGCAAXXXXXXXXXXAGCTTGTCCTCCACGTTCTTCCCTTTAGTCACCACTTAGA | lmxll |
| Marker1353 | CATTGAGGACAATGTTCAATTCATGTCTGGGGGGAAGGTTXXXXXXXXXXGCAGATATCATGTGCCTGCTCTCACTCATGCATGAACTCA | hkxhk |
| Marker1353 | CATTGAGGACAATGTTCAATTCATGTCTGGGGGGAAGGTTXXXXXXXXXXGCAGATATCATGTGCCTGCTCTCACTCATGAATGAACTCA | hkxhk |
| Marker1361 | TGTTATACTTACATTGAACTTGTAATGTCGAACCATGCATXXXXXXXXXXTTGTTTTTACGTACGGCAGCAGGCCAGAAACTTCTTTATC | lmxll |
| Marker1361 | TGTTATACTTACATTGAACTTGTAATGTCGAACCATGCATXXXXXXXXXXTTGTTTTTACGTACAGCAGCAGGCCAGAAACTTCTTTATC | lmxll |
| Marker1362 | TGTGTTTTTGTTCTTGAACTTGCAGACTGAAGCAGCTAAGXXXXXXXXXXGGAGAGGAAAAAAAATCTCTTCAGACTGTATAACCCCATT | nnxnp |
| Marker1362 | TGTGTTTTTGTTTTTGAACTTGCAGACTGAAGCAGCTAAGXXXXXXXXXXGGAGAGGAAAAAAAATCTCTTCAGACTGTATAACCCCATT | nnxnp |
| Marker1363 | TCAGTTGGATCGGATGAAGATTTGAGCAAAAATAGATCACXXXXXXXXXXGTGCATCAAAAGAATCAAGTTTTAGCTGCAATATGAGACT | lmxll |
| Marker1363 | TCAGTTGGATCGGATGAAGATTTGAGCAAAAATAGATCACXXXXXXXXXXGTGCATCAAAAGAATCAAGTTTTAGCTGCAATATGTGACT | lmxll |
| Marker1364 | AAGCACTTCCAACAAAACTTCTATCAAAAGCACGTCCAACXXXXXXXXXXGATCGATATAGACAGGATTCAGGAAGGTTGTATTTAGCAG | nnxnp |
| Marker1364 | AAGCACTTCCAACAAAACTTCTATCAAAAGCACGTCCAACXXXXXXXXXXGATCGATATAGACGGGATTCAGGAAGGTTGTATTTAGCAG | nnxnp |
| Marker1367 | TATTCTTTGCACACCAACCATGTTCAATTTTCTCAGCTTTXXXXXXXXXXGGGGGAGGGGAAAATCGAACCTAGGATCTCGAATACAGTG | nnxnp |
| Marker1367 | TATTCTTTGCACACCAACCATGTTCAATTTTCTCAGCTTTXXXXXXXXXXTTGGGGAAGGGGAAAATCGAACCGGATCTCGAATACAGTG | nnxnp |
| Marker1372 | GGTCCATCACACATTATATTTCAATGATCTAAACCGTTTAXXXXXXXXXXTACATATCCATGCAGATATCGCCTCTGCCCTACCAATTGC | lmxll |
| Marker1372 | GGTCCATCACATATTATATTTCAATGATCTAAACCGTTTAXXXXXXXXXXTACATATCCATGCAGATATCGCCTCTGCCCTACCAATTGC | lmxll |
| Marker1378 | ATATACTAAAGAAACTTTGTTCTGATCACTTTCGCCATACXXXXXXXXXXTACTTGATAAGATGTAGACAAACAGCTTCTAAATCCTTGA | lmxll |
| Marker1378 | ATATACTAAAGAAACTTTGTTCTGATCACTTTCGCCATACXXXXXXXXXXTACTTGATAAGATGTAGACAAACAGCTTCTAAATCCTTGG | lmxll |
| Marker1381 | TTATGACATCATAAGATTCATACAAGACTATGACAAAAAAXXXXXXXXXXTCTTCTGCACTGGCCTTACAATTATACTCGTCGATGATTC | nnxnp |
| Marker1381 | TTATGACATCGTAAGATTCATACAAGACTATGACAAAAAAXXXXXXXXXXTCTTCTGCACTGGCCTTACAATTATACTCGTCGATGATTC | nnxnp |
| Marker1383 | ATTACTTGAGGAGTAAGGAGAGATTAGCACTAAAATTGTCXXXXXXXXXXCCAATCCAACCCCAAAAATGATCCTGATCATCAAATGAAA | lmxll |
| Marker1383 | ATTACTTGAGGAGTAAGGAGAGATTAGCACTAAAATTGTCXXXXXXXXXXCCAATCCAACCCCAAAAATGATCCTGATCATCAAATGAAT | lmxll |
| Marker1389 | ATCCTTCCGCTGCCATGGATATCGTATCCAAATCCGAATCXXXXXXXXXXAAGAATTGTGGTAAACCTCCAGTTCACTACGAAGAAGATC | nnxnp |
| Marker1389 | ATCCTTCCGCTGCCATGGATATCGTATCCAAATCCAAATCXXXXXXXXXXAAGAATCGTGGTAAACCTCCAGTTCACTACGAAGAAGATC | nnxnp |
| Marker1395 | CCATGTGGTTGGGGGTTCGATCCCCGCCCATGGGAATGGAXXXXXXXXXXTCAGATTTTGTAACAATTCATGAGTTCAGATTTTGAAAAT | nnxnp |
| Marker1395 | CCATGTGGTTGGGGGTTCGATCCCCGCCCATGGGAATGGAXXXXXXXXXXTCAGATTTTGAAACAATTCATGAGTTCAGATTTTGAAAAT | nnxnp |
| Marker1404 | TAGTTGAACTATTATCACAATGAATCACAGTAGGCCCATGXXXXXXXXXXATTAGACACATTGCATACATAATATCAGGCCGAGTGGTTG | lmxll |
| Marker1404 | TAGTTGAACTATTATCACAATGAATCACTGTAGGCCCATGXXXXXXXXXXATTAGACACATTGCATACATAATATCAGGCCGAGTGGTTG | lmxll |
| Marker1408 | TCTCTGTGGTCCATTATACACTATTGAACTTTTGACCTCCXXXXXXXXXXTGTTAGTGAAAGCATAGTCTCCGATTGTCAAATCGCATCT | lmxll |
| Marker1408 | TCTCTGTGGTCCATTATACACTATTGAACTTTTGATCTCCXXXXXXXXXXTGTTAGTGAAAGCATAGTCTCCGATTGTCAAATCGCATCT | lmxll |
| Marker1412 | ACCGCATCATTCCAAACTGCATAGCACACCTCTTATTTTGXXXXXXXXXXCTTCTAATGGCTCAAAAAGAAGCAAAGACTTCTGAAGAAG | lmxll |
| Marker1412 | ACCGCATCATTCCAAACTGCATAGCACACCTCTTTTTTTGXXXXXXXXXXCTTCTAATGGCTCAAAAAGAAGCAAAGACTTCTGAAGAAG | lmxll |
| Marker1415 | AAGGTAATTGCAAAAGAATGAGACTGTTTTGCTTCTTGTTXXXXXXXXXXAACGCATCGCTCAGAGTTTGTCATCAACAGAACTAAGGAC | nnxnp |
| Marker1415 | AAGGTAATTGCAAAAGAATGAGACTGTTTTGCTTCTTGTTXXXXXXXXXXAACGCATCGCTCAAAGTTTGTCATCAACAGAACTAAGGAC | nnxnp |
| Marker1420 | TGGGTCTGCTTGAATTTCATGCGGCAATCAAACAATGGCTXXXXXXXXXXGGACAAATACCTTGCTAAAACAGTGAGAGCCACATGTATA | hkxhk |
| Marker1420 | TGGGTCTGCTTGAATTTCATGCGGCAATCAAACAATGGCTXXXXXXXXXXGGGCAAATACCTTGCTAAAACAGTGAGAGCCACATGTATA | hkxhk |
| Marker1423 | GAAACTGAGAAGTGACAGAGGAGGAGAGTACACATCACAGXXXXXXXXXXCACAAGCTGGAGGAAACTGGAATGAAAGGGATATTTGTGG | nnxnp |
| Marker1423 | GAAACTGAGAAGTGACAGAGGAGGAGAGTACACATCACAGXXXXXXXXXXCACAAGCTTGAGGAAACTGGAATGAAAGGGATATTTGTGG | nnxnp |
| Marker1427 | CTTGTCAACATTATATCTTTATGAAAAGGTTAGGTAAGGAXXXXXXXXXXATGAGAGCCAGAAATAAGCATGACTTGTTTTGAAATATAC | nnxnp |
| Marker1427 | CTTGTCAACATTATATCTTTATGAAAAGGTTAGGTAAGGAXXXXXXXXXXATGAGAGCCAGAAATAAGGATGACTTGTTTTGAAATATAC | nnxnp |
| Marker1430 | ACATCTAATGATGTCTTCATCAGAAGAAAAATTGGGATAAXXXXXXXXXXACACGGGATGAGGGAGAATAGGGGTTTTTTTTCTAAGTCC | lmxll |
| Marker1430 | ACATCTAATGATGTCTTCATCAGAAGAAAAATTGGGATAAXXXXXXXXXXGACACGGGATGAGGGAGAATAGGGGTTTTTTTCTAAGTCC | lmxll |
| Marker1435 | TTTCTGAGTAAGAGTACCTTATTGGCAAGAAGTTGCTATCXXXXXXXXXXGGGGACAACATCAAAGACTTGACTACAGGTTTTCGAAAAG | nnxnp |
| Marker1435 | TTTCTGAGTAAGAGTACCTTATTGGCAAGAAGTTGCTATCXXXXXXXXXXGGGGACAACATCAATGAATTGACTACAGGTTTTCGAAAAG | nnxnp |
| Marker1438 | TATCATTGGCCGTGATTTTGTTCGGCCTCGGCCTCAGCCTXXXXXXXXXXTGGTATGGGACGTGCAGGTCTGCTTGTCCAGTTATATGAA | nnxnp |
| Marker1438 | TATCATTGGCCGTGATTTTGTTCGGCCTCGGCCTCGGCCTXXXXXXXXXXTGGTATGGGACGTGCAGGTCTGCTTGTCCAGTTATATGAA | nnxnp |
| Marker1441 | TTACGGCAACTCCACCGCAAAGTTTGGCAATTCTTTCAGAXXXXXXXXXXTCTGATACACAGGACCTACAATGACGTCGTTTTATAGACC | nnxnp |
| Marker1441 | TTACGGCAACTCCACCGCAAAGCTTGGCAATTCTTTCAGAXXXXXXXXXXTCTGATACACAGGACCTACAATGACGTCGTTTTATAGACC | nnxnp |
| Marker1448 | ACTGGAGGAACATCAAAATGGGTAGAAGCAATCTAATATTXXXXXXXXXXTAGAAAATAAGATAAATAAATTCAACGTCAGCTAATTTCG | lmxll |
| Marker1448 | ACTGGAGGAACATCAAAATGGGTAGACGCAATCTAATATTXXXXXXXXXXTAGAAAATAAGAAAAATAAATTCAACGTCAGCTAATTTCG | lmxll |
| Marker1452 | CAAGGCATCAAATACTTTGTTACCTATGAGCTGGGATCAAXXXXXXXXXXGAAGGTCTCACAAGCCCCTTTTTATTTTATTTTTTTATAT | hkxhk |
| Marker1452 | CAAGGCATCAAATACTTTGTTACCTATGAGCTGGGATCAAXXXXXXXXXXGAAGGTCTCACAAGCCCCTTTTTATTTTTATTTTTTATAT | hkxhk |
| Marker1461 | GTCATGGGCTGGGTAATTGAAAATCTCATGTACCTGGTACXXXXXXXXXXTCGCAATGATTGGACTTCAATAGAAATCCCTTGCTTCTTA | nnxnp |
| Marker1461 | GTCATGGGTTGGGTAACTGAAAATCTCATGTACCTGGTACXXXXXXXXXXTCGCAATGATTGGACTTCAATAGAAATCCCTTGCTTCTTA | nnxnp |
| Marker1462 | TGGAATAAGTCAATCTCTTGGTCCTCGATAAAGATATAGAXXXXXXXXXXTATTTATACCTCCCACTAAAAAGTCACACCAATTCTATAT | nnxnp |
| Marker1462 | TGGAATAAGTCAATCTCTTGGTCCTCGATAAAGATATAGAXXXXXXXXXXTATTTATACCTCCCACTAAAAAGTCACACCAATTCCATAT | nnxnp |
| Marker1471 | TAAGAACAAAAATATACGTACCAGATAAGACAAGAAAAACXXXXXXXXXXAAAGTTCAGACTAAATTATGAAAGAGTTCATTTATAATGG | nnxnp |
| Marker1471 | TAAGAACAAAAATATACGTACCAGATAAGACAAGAAAAACXXXXXXXXXXAAAGTTCAGACTAAATTATGAAAGAATTTATTTATAATGG | nnxnp |
| Marker1480 | AAGGAAAGCATTTCTACTTTCACTTTACTTTCTTTTCCTCXXXXXXXXXXCATCACTCGAGCTCCCAAAGATTGCATTCTTAGCTTGAGC | nnxnp |
| Marker1480 | AAGGGAAGCATTTCTACTTTCACTTTACTTTCTTTTCCTCXXXXXXXXXXCATCACTCGAGCTCCCAAAGATTGCATTCTTAGCTTGAGC | nnxnp |
| Marker1482 | ATGGGATTGTAGAGTCCAATGGTAAAAAAAAACCCTCACTXXXXXXXXXXAGAAACCATATCTACTGTAGCGACATATATATACTCGATG | lmxll |
| Marker1482 | ATGGGATTGTAGAGTCCAATGGTAAAAAAAACCCCTCACTXXXXXXXXXXAGAAACCATATCTACTGTAGCGACATATATATACTCGATG | lmxll |
| Marker1486 | AGATCCCGAATGATGGGCTACGTCCACGGAGAAGTATGTTXXXXXXXXXXGGCCAGATATTGTACAAACAGGAGTCTCCCAAGTTCCCGA | lmxll |
| Marker1486 | AGATCCCGAATGATGGGCTACGTCCACGGAGAAGTATGTTXXXXXXXXXXGGCCAGATATTGTACAAACAGGAGTCCCCCAAGTTCCCGA | lmxll |
| Marker1509 | ACTTGCACTGGTTTTTCCATGCAGAGAATTTTCACCGAGTXXXXXXXXXXAAGTGATCAGCTTAGTCCCTGGGAAATTGCAAAATGTGGC | nnxnp |
| Marker1509 | ACTTCCACTGGTTTTTCCATGCAGAGAATTTTCACCGAGTXXXXXXXXXXAAGTGATCAGCTTAGTCCCTGGGAAATTGCAAAATGTGGC | nnxnp |
| Marker1510 | TAACGTGCGGATATCCAGATGAGATAATAATTGTATGAAAXXXXXXXXXXAGTGCATCAATCTTTCATGCCACACTTTTTATGTATTTTC | lmxll |
| Marker1510 | TAACGTGCGGATATCCAGATGAGATAATAATTGTATAAAAXXXXXXXXXXAGTGCATCAATCTTTCATGCCACACTTTTTATGTATTTTC | lmxll |
| Marker1513 | TCTACTAATATATATAACGTATTAGACAAGGAAAGAGACGXXXXXXXXXXACCGGCATTTTGGTTTGTTGGGTGGGTTGGTGGGGACTGG | lmxll |
| Marker1513 | TCTACTAATCTATATAACGTATTAGACAAGGAAAGAGACGXXXXXXXXXXACCGGCATTTTGGTTTGTTGGGTGGGTTGGTGGGGACTGG | lmxll |
| Marker1529 | ACGGATATTCAATCTAAGTAGATTTCACTAAATACATTCCXXXXXXXXXXACTGCAGCTGCAATCTCATACTTGGATGGCCATGATGATG | hkxhk |
| Marker1529 | ACGGATATTCAATCTAAGTAGGTTTCACTAAATACATTCCXXXXXXXXXXACTGCAGCTGCAATCTCATACTTGGATGGCCATGATGATG | hkxhk |
| Marker1534 | AAAAATGTACATAAAATACTATTATTTATTGTCAAAAATAXXXXXXXXXXAGATAGAGATGAAGAACAAGAAAGAAAAATGTGCATATGG | nnxnp |
| Marker1534 | AAAAATGTACATAAAATACTATTATTGATTGTCAAAAATAXXXXXXXXXXAGATAGAGATGAAGAACAAGAAAGAAAAATGTGCATATGG | nnxnp |
| Marker1536 | TTACCCTTACTTCTGTCATATTTACTTTTCTGGAGGTAAAXXXXXXXXXXAATCAAGTAAGTAATATGATTTTAGATCCAATTGCAACAG | lmxll |
| Marker1536 | TTACCCTTACTTCTGTCATATTTACATTTCTGGAGGTAAAXXXXXXXXXXAATCAAGTAAGTAATATGATTTTAGATCCAATTGCAACAG | lmxll |
| Marker1540 | ACGTTTACTATTCCATAATTCTATCTGCAACATAGAAACCXXXXXXXXXXGCTTGGATCCCAATCTTTGCAAGGTACGATTAGTTCTAAT | lmxll |
| Marker1540 | ACGTTTACTATCCCATAATTCTGTCTGCAACATAGAAACCXXXXXXXXXXGCTTGGATCCCAATCTTTGCAAGGTACGATTAGTTCTAAT | lmxll |
| Marker1546 | GAACATCTACAGCAGCTTACAGCAAAAATTACCTCTGTAAXXXXXXXXXXGTTCAAAATTCACATAAAACATAAAAGCTTTACAAACTTT | lmxll |
| Marker1546 | GAACACCTACAGTAGCTTACAGCAAAAATTACCTCTGTAAXXXXXXXXXXGTTCAAAATTCACATAAAACATAAAAGCTTTACAAACTTT | lmxll |
| Marker1547 | GGGCTCTTGCTGGGTTTGTGGAAAACCAGGCCACAAAGCAXXXXXXXXXXAAAAACCTTGTGTCAGGCCCATTGTTGAGTAACAAGGGCT | lmxll |
| Marker1547 | GGGCTCTTGCTTAGTTTGTGGAAAACCAGGCCACAAAGCAXXXXXXXXXXAAAAACCTTGTGTCAGGCCCATTGTTGAGTAACAAGGGCT | lmxll |
| Marker1551 | CCCAAACCAAGCACATAAACAAGTAGAGCACGAAGAGTAGXXXXXXXXXXAGAGAGAGAGAGAGAGAGAGTGAGAGAGGCTGACTGGTTT | lmxll |
| Marker1551 | CCAAAACCAAGCACATAAACAAGTAGAGCACGAAGAGTAGXXXXXXXXXXAGAGAGAGAGAGAGAGAGAGTGAGAGAGGCTGACTGGTTT | lmxll |
| Marker1560 | GGCAGGGGACAGTGTAACAGCTGTTCATGTTCAAGAAGCAXXXXXXXXXXTCTCTCTCTCTCTCTCTCTCTCTCTCTCTCTCATGTATGG | efxeg |
| Marker1560 | GGCAGGGGACAGTGTAACAGCTGTTCATGTTCAAGAAGCAXXXXXXXXXXAATCTCTCTCTCTCTCTCTCTCTCTCTCTCTCATGTATGG | efxeg |
| Marker1563 | TACAATTGTGGGTTAGTAACTTTTTTATTTATTCAAGCGAXXXXXXXXXXCTCTCTACAGGAAAAAAAATATTTGGGTTTAGAAATTTTT | efxeg |
| Marker1563 | TACAATTGTGGGTTAGTAACTTTTTTATTTATTCAAGCGAXXXXXXXXXXCTCTCTACAGGAAAAAAAATATTTGGGTTTAGAAAATTTT | efxeg |
| Marker1564 | TCTAATTGAACTTGGGAACTTATCTGCTTGAGTTAGTTATXXXXXXXXXXCTTGACTTTTATACTTCTATATGGTCAACAGCAAGAGCTC | lmxll |
| Marker1564 | TCTAATTGAACTTGGGAACTTATCTACTTGAGTTAGTTATXXXXXXXXXXCTTGACTTTTATACTTCTATATGGTCAACAGCAAGAGCTC | lmxll |
| Marker1566 | TATACGTGCCCCTGCATCTCCTCTATTCATTGGGTAGAAAXXXXXXXXXXAGAATCACTATTACAAAATAGCTAAACGACAATGATAGAG | lmxll |
| Marker1566 | TATACGTGCCCCTGCATCTCCTCTATTCATTGGGTAGAAAXXXXXXXXXXAGAATCACTATTACAAAATAGCTAAACGACAACGATAGAG | lmxll |
| Marker1567 | GCTTGGCAAGTTTGCCTTCTAAACAAGTAGTATAGACAGAXXXXXXXXXXAGCAAATGCTAAGAAAGCTTTGGCACATGTAAAACTTGCT | nnxnp |
| Marker1567 | GCTTGGCAAGTTTTCCTTCTAAACAAGTAGTATAGACAGAXXXXXXXXXXAGCAAATGCTAAGAAAGCTTTGGCACATGTAAAACTTGCT | nnxnp |
| Marker1587 | ATGGGTTAGAAAGCAAAATGGGTTTGATAGTCCTACCGTTXXXXXXXXXXTGAGAATGTTGTGACGTGGAAGGGTGAAGAATGTAGCAAA | nnxnp |
| Marker1587 | ATGGGTTAGAAAGCAAAGTGGGTTTGATAGTCCTACCGTTXXXXXXXXXXTGAGAATGTTGTGATGTGGAATGGTGAAGAATGTAGCAAA | nnxnp |
| Marker1593 | AAGAAAAGAAAGAGACAAAGGAAGAAAAGGAAATGGCCATXXXXXXXXXXTTGTCCTCATAAGACATTTTACAAAGGGGTAAATTTGTCT | nnxnp |
| Marker1593 | AAGAAAAGAAAGAGACAAAGGAAGAAAAGGAAATGGCCATXXXXXXXXXXTTGTCCTCATAAGACATTTTACAATGGGGTAAATTTGTCT | nnxnp |
| Marker1595 | GTGGTTTATTTCCACATCTTCTCCTCAAAAATGTCTTCCTXXXXXXXXXXAACACAAGGATTCTCCTATAGATGCCCAAATATTAGGCAT | nnxnp |
| Marker1595 | GTGGTTTATTTCCACATCTTCTCCTAAAAAATGTCTTCCTXXXXXXXXXXAACACAAGGATTCTCCTATAGATGCCCAAATATTAGGCAT | nnxnp |
| Marker1604 | TACTTTACTGTGAGTAACTGCCATGCCAATAAGGAATATAXXXXXXXXXXCCTCTCTGCCATGCGGTATAAGAAACAGAGTGTGTGTTTG | nnxnp |
| Marker1604 | TACTTTACTGTGAGTAACTGCCATGCCAATAAGGAATATAXXXXXXXXXXCCTCTCTGCCACGCAGTATGAGAAACAGAGTGTGTGTTTG | nnxnp |
| Marker1607 | TCTAACCAGGCTTATGAGTAAAAACATCGCAATTGTTGAGXXXXXXXXXXAAAATGCAACTTATGAAGTTTCACAGAGAAAGAGGTCTAT | lmxll |
| Marker1607 | TCTAACCAGGCTTATGAGTAAAAACATCCCAGTTGTTGAGXXXXXXXXXXAAAATGCAACTTATGAAGTTTCACAGAGAAAGAGGTCTAT | lmxll |
| Marker1614 | AAAAGTATCAATTATCATCAATTATTACCAAAAAAGGGAAXXXXXXXXXXGTGGTTTGGTACTTATCACATCACCAACCCTCTAATTAGC | lmxll |
| Marker1614 | AAAAGTTATCAATTATCATCAATTATTACCAAAAAAGGGAXXXXXXXXXXGTGGTTTGGTACTTATCACATCACCAACCCTCTAATTAGC | lmxll |
| Marker1617 | GTGCTCAAACTAACTTACTGTTCTCTGGCTTGAACTTTAGXXXXXXXXXXGATAGTGGTCTGTATGCCCTATATACCATACATAATTTTT | nnxnp |
| Marker1617 | GTGCTCAAACTAACTTACTGTTCTCTGGCTTGAACTTTAGXXXXXXXXXXGATAGTGGTCTGTATGCCCTATATACCATACAGAATTTTT | nnxnp |
| Marker1619 | ATGATGATGATAATGATGTTGTTGACGAAGCTCGGCCCAGXXXXXXXXXXAGGCCTTGCCCCCTCTTATTTTTTGCTATTGTTGAAAAGT | nnxnp |
| Marker1619 | ATGATGATGATAATGATGTTGTTGACGAAGCTCAGCCCAGXXXXXXXXXXAGGCCTTGCCCCCTCTTATTTTTTGCTATTGTTGAAAAGT | nnxnp |
| Marker1626 | AACATTGCATACATCAAAACAAAGCATAATTCCCACTACCXXXXXXXXXXCTGATATGTAAATTTTATAATAAATATGAACACGTGGCAA | hkxhk |
| Marker1626 | AACATTGCATACATCAAAACAAAGCATAATTCCCACTACCXXXXXXXXXXCTGATATGTAAATTTTATAATAAATATGAACACGTGACAA | hkxhk |
| Marker1637 | TCACACTGATGATGACTCATACTTTGTAATTCAGTACTTGXXXXXXXXXXCTAATTCTCTAAACTGTTTTTGAGAATCGAATTTGCAATG | lmxll |
| Marker1637 | TCACACTGATGATGACTCATACTTTGTAATTCAGTACTTGXXXXXXXXXXCTAATTCTCTAAACCGTTTTTGAGAATCGAATTTGCAATG | lmxll |
| Marker1640 | ACTGTTGTATACTTTGCAACAACTCCAACCTCACATGAGTXXXXXXXXXXCTAATTCTCCAAAGAAACCTCCAAGTTCATCTCTTGCATA | lmxll |
| Marker1640 | ACTATTGTATACTTTGCAACAACTCCAACCTCACATGAGTXXXXXXXXXXCTAATTCTCCAAAGAAACCTCCAAGTTCATCTCTTGCATA | lmxll |
| Marker1642 | CAGGATCTTCTGCACAAAGATTCTGCCACAAATTTCACATXXXXXXXXXXTTCTTTGTATAGCATAGACAACCTATCTGAGAGAATAAAT | lmxll |
| Marker1642 | CAGGTTCTTCTGCACAAAGATTCTGCCACAAATTTCACATXXXXXXXXXXTTCTTTGTATAGCATAGACAACCTATCTGAGAGAATAAAT | lmxll |
| Marker1651 | AAAGAAGAAGATAAAGCTTGAAAAAACCAAGTCATAATAAXXXXXXXXXXACATTTTCTTTATTTTTGGACTCCTGACAAATTTGTAGAT | lmxll |
| Marker1651 | AAAAGAAGAAGATAAAGCTTGAAAAAACCAAGTCATAATAXXXXXXXXXXACATTTTCTTTATTTTTGCACTCCTGACAAATTTGTAGAT | lmxll |
| Marker1661 | TATATTGGTACGATAAAGTTCGTCATATCGTTTGTTTTCTXXXXXXXXXXAATTGTTACCTTTTTCAACTGCTTTTAGGTCTGCCCTTGT | nnxnp |
| Marker1661 | TATATTGGTACGATAAAGTTCGTCATATCGTTTGTTTTCTXXXXXXXXXXAATTGTTACCTCTTTCAACTGCTTTTAGGTCTGCCCTTGT | nnxnp |
| Marker1667 | TTGATGTAGTAAGTACCCATATGTCGTCACGTCATACGCGXXXXXXXXXXTGCATCATGCCAAACACACACCAAAGTTCATAAGTTTTGC | nnxnp |
| Marker1667 | TTGATGTAGTAAGTACCCATATGTCGTCACGTCATACGCGXXXXXXXXXXTGCATCATGCCAAACACACACCCAAGTTCATAAGTTTTGC | nnxnp |
| Marker1669 | AAAAGAAGAAAAAACACACGGCCAAGATCAATCACTTTCAXXXXXXXXXXACACTTAGTGATTAGAGACAAGTCAAATTCCGAGCGAGGA | hkxhk |
| Marker1669 | AAAAGAAGAAAAAACACACGGCCAAGATCAATCACTTTCAXXXXXXXXXXACACTTAGTGATTAGAGACAAGTCAAATTCCGAACGAGGA | hkxhk |
| Marker1672 | AATCATTAGTAAAGTCAAAGGATAAGTGTGTTCAGAAATCXXXXXXXXXXCAACAAAAATAAAACTGATCTACACCACACACTGTATTAC | lmxll |
| Marker1672 | AATCATTAGTAAAGTAAAAGGATAAGTGTGTTTGGAAATCXXXXXXXXXXCAACAAAAATAAAACTGATCTACACCACACACTGTATTAC | lmxll |
| Marker1680 | AAGCAAATAAAGAATACAAATTTCAATTGAAATCGAAATAXXXXXXXXXXCTATCTCTGTGATGTTGTAGCAATCTATATGTTCTTTTCT | lmxll |
| Marker1680 | AAGCAAATAAAGAATACAAATTTCAATTGAAATCGAAATAXXXXXXXXXXCTATCTCCGTGATGTTGTAGCAATCTATATGTTCTTTTCT | lmxll |
| Marker1681 | AAGAAATGTCCAATGCCATTTTTGCCAACCAAGCCTCCGTXXXXXXXXXXGACAATTTTGGGGGAGAATTTCAATTCTGTGTTGCTTTTG | nnxnp |
| Marker1681 | AAGAAATGTCCAATGCCATTTTTGCCAACCAAGCCTCCGTXXXXXXXXXXGAGAATTTTGGGGGAGAATTTCAATTCTGTGTTGCTTTTG | nnxnp |
| Marker1682 | GTTGGAAGTAGCAGTATTAGACTCAGCGATTCTTTTGTTGXXXXXXXXXXAGTCGGCCATCTTTGTATAGCGGCAATGAAGCCTCAGTAT | lmxll |
| Marker1682 | GTTGGAAGTAGCAGTATTAGACTCAGCGATTCTTTTGTTGXXXXXXXXXXAGTCGGCCATCTTTGTATAGCGGCAATGAAGCCTTAGTAT | lmxll |
| Marker1683 | CCATCTCACAAGCTTCAACCATCCTACCTTCCATAAACAAXXXXXXXXXXAAACCCGTGAATTACCGTATTGTAAGTTACAACATTAGGC | lmxll |
| Marker1683 | CCATCTCATAAGCTTCAACCATCCTACCTTCCATAAACAAXXXXXXXXXXAAACCCGTGAATTACCGTATTGTAAGTTACAACATTAGGC | lmxll |
| Marker1688 | TGGGGTGTTTCCAAAACAAGTCAATTACAATATCACAAGAXXXXXXXXXXCTCCAACTTCCAATCATCCCATCACCAATATTGACACATT | lmxll |
| Marker1688 | TGGGGTGTTTCCAAAACAAGTCAATTACAATATCACAAGAXXXXXXXXXXCTCCAACTTCCAATCATCCCATCACCAATATTGACACCTT | lmxll |
| Marker1690 | AATGTCTTTCTCTTTTACTTATTTTTTACAAGAAGATCTTXXXXXXXXXXATTTGACGTTCAGTGTACCAATATTTGGTTCGAATTATTC | hkxhk |
| Marker1690 | AATGTCTTTCTCTTTTACTTATTTTTTACAAGAAGATCTTXXXXXXXXXXATTTGACGTTCAGTGTACCAATATTGGGTTCGAATTATTC | hkxhk |
| Marker1692 | AATAGCTGTTGAATGGATGTAAATTTATTTTTTTTTATTTXXXXXXXXXXAAGGAAATGGAATGGGACAGTCTTTAGCACAAGCAAGGAA | hkxhk |
| Marker1692 | AATAGCTGTTGAATGGATGTAAATTTCTTATTTTTTATTTXXXXXXXXXXAAGGAAATGGAATGGGACAGTCTTTAGCACAAGCAAGGAA | hkxhk |
| Marker1698 | TGGGATGGTTTACATTCTTGAAGTAAGAAATGAGGAAACAXXXXXXXXXXTCGGATTCTACAAAAGTACTTACAAAACAGTTGGGTTCGG | nnxnp |
| Marker1698 | TGGGATGGTTTACATTCCTGAAGTAAGAAATGAGGAAACAXXXXXXXXXXTCGGATTCTACAAAAGTACTTACAAAACAGTTGGGTTCGG | nnxnp |
| Marker1700 | CGGGTGGGAACAAATGTTGTTCATTGGAATATATGTGGCAXXXXXXXXXXTGTTTGGACATCCTCTTGGATATCTTCTTGGTTATACTGC | lmxll |
| Marker1700 | CGGGTGGGAACAAATGTTGTTCATTGGAATATATGCGGCAXXXXXXXXXXTTTTTGGACATCCTCTTGGATATCTTCTTGGTTATACTGC | lmxll |
| Marker1701 | AGGTCCTGGAAAATGCTTCTGCCTCTATGAGTCTGTATACXXXXXXXXXXTTTTGGGCTTCTGATGTCGTTTGGCCTTCCAAAAGTGCAT | nnxnp |
| Marker1701 | AGGTCCTGGAAAATGCTTCCGCCTCTATGAGTCTGTATACXXXXXXXXXXTTTTGGGCTTCTGAGGTCGTTTGGCCTTCCAAAAGTGCAT | nnxnp |
| Marker1704 | AAATTACTGGCACACCCACTTGAAAAAACGCTTCAAGGCGXXXXXXXXXXGCTGGTTCATCATACAATGACGCCTCCAATGATTATTGGA | lmxll |
| Marker1704 | AAATTACTGGCACACCCACTTGAAAAAACGCTTCAAGGCGXXXXXXXXXXGCTGGTTCATCATACAATGACGCCTCCTATGATTATTGGA | lmxll |
| Marker1710 | AAGAAAAGGAGGTAAGTATGACAAATCGGCCAGAAAATAAXXXXXXXXXXTTCCATAAAGCAAGGTGAAAGTACACTGACTAAAAAGCAA | lmxll |
| Marker1710 | AAGAAAAGGAGGTAAGTATGACAAATCGGCCAGAAAATAAXXXXXXXXXXTTCCATAGAGCAAGGTGAAAGTACACTGACTAAAAAGCAA | lmxll |
| Marker1714 | TAATTTATGCATTAGAGCATGTGAGTTGGGCTGATGCTACXXXXXXXXXXTCGGAGTGGTGCTAATTACTAAAATATCTTCATTGTTAGG | nnxnp |
| Marker1714 | TAATTTATGCATTAGAGCATGTGAGTTGGGCTGATGCTACXXXXXXXXXXTTGGAGTGGTGCTAATTACTAAAATATCTTCATTGTTAGG | nnxnp |
| Marker1724 | AAATAATTATCAAAATTACCTGATAAAAGTATAATTTATCXXXXXXXXXXCTTATGCCCAAATTTGTAGCGGAGACTGCTTGGAGAACAG | nnxnp |
| Marker1724 | AATAATTATCAAAATTACCTGATAAAAGTATAATTTATCTXXXXXXXXXXCTTATGCCCAAATTTGTAGCGGAGACTGCTTGGAGAACAG | nnxnp |
| Marker1739 | TCTTGGCCGGAGAGGATTTAGATGTACTTGCATTGAAGCAXXXXXXXXXXTCACACAATGCTTATAAGATAATTTTTTTTCTTCTCTTGT | lmxll |
| Marker1739 | TCTTGGCCAGAGAGGATTTAGATGTACTTGCATTGAAGCAXXXXXXXXXXTCACACAATGCTTATAAGAGAATTTTTTTTCTTCTCTTGT | lmxll |
| Marker1740 | CCCTCACCTATCTGGGCCCATAAGATCTTATTGGACCAATXXXXXXXXXXTTTGGAACTGTCCGTTTTTCGTGCAGTATGATTTCTGCTT | lmxll |
| Marker1740 | CCCTCACCTATCTGGGCCCGTAAGATCTTATTGGACCAATXXXXXXXXXXTTTGGAACTGTCCGTTTTTCGTGCAGTATGATTTCTGCTT | lmxll |
| Marker1742 | TCAAGAATGTATGACAATAAGAGTCCAAATTGGCTTCCATXXXXXXXXXXTGAAATGGTTTATGAAGACTGAAAAAGGCACTGGTTATAT | nnxnp |
| Marker1742 | TCAAGAATGTATGACAATAAGAGTCCAAATTGGCTTCCATXXXXXXXXXXTGAAATGGTTTATGAAGACTGAAAGAGGCACTGGTTATTT | nnxnp |
| Marker1747 | TCTCACTATTCCAGGTTCCAATTGTACATGCCTAAAAATTXXXXXXXXXXTCTACGGATGTCAAATATTGTGGTCAGTTTCCGTTTCACT | nnxnp |
| Marker1747 | TCTCACTATTCCAGGTTCCAATTGTGCATGCCTAAAAATTXXXXXXXXXXTCTACGGATGTCAAATATTGTGGTCAGTTTCCGTTTCACT | nnxnp |
| Marker1750 | AGCACATCTATCTAATCTAAGATTTAGAAAATCAATCATTXXXXXXXXXXAGCCAATCCAACGAGTGCGAGGCCACTCTTTATTGACGTC | nnxnp |
| Marker1750 | AGCACATCTATCTAATCTAAGATTTACAAAATCAATCATTXXXXXXXXXXAGCCAATCCAACGAGTGCGAGGCCACTCTTTATTGACGTC | nnxnp |
| Marker1755 | TGTACAAAGCAACAAGATTGAGAGAGAGAGAGAGAGAGAGXXXXXXXXXXTGGGTCAAGAACTTTACCTTGATTGAAGGGTTTTGAATTG | lmxll |
| Marker1755 | TGTACAAAGCAACAAGAGAGAGAGAGAGAGAGAGAGAGAGXXXXXXXXXXTGGGTCAAGAACTTTACCTTGATTGAAGGGTTTTGAATTG | lmxll |
| Marker1767 | TAATGAAAAAAAATATTGCCAACGGGGTCTAGCACAATAGXXXXXXXXXXTGGCTGAGTGGAGTGGTAACGAGTCAAATAATTACTGTCC | lmxll |
| Marker1767 | TAATGAAAAAAAAATATTGCCAACGGGGTCTAGCACAATAXXXXXXXXXXTGGCTGAGTGGAGTGGTAACGAGTCAAATAATTACTGTCC | lmxll |
| Marker1771 | TATTTTTCCACAGCAGAAAATCTCAGTAAAAGACAGTGGTXXXXXXXXXXTTGGATTGTGGCTTCTATGCTTCTACGTATGTACGAATCA | lmxll |
| Marker1771 | TATTTTTCCACAGCAGAAAATCTCAGTAAAAGCCAGTGGTXXXXXXXXXXTTGGATTGTGGCTTCTATGCTTCTACGTATGTACGAATCA | lmxll |
| Marker1779 | CAAATAAGAAATGAAACATATATAAGACCTAAACAAAAATXXXXXXXXXXAAAATATATGCCTCAACCTGTGCATGCCATTCTAATTTTT | lmxll |
| Marker1779 | CAAATAAGAAATGAAACATATATAAGACCTAAACAAAAATXXXXXXXXXXAAATATATGCCTCAACCTGTGCATGCCATTCTAATTTTTT | lmxll |
| Marker1785 | TCACATCTCTCTTGAACTTGAGAGCTGCAGTGACCAGGCCXXXXXXXXXXCCAGTGGCGGATTTAGGAATTTTTCAGGGAAGGGGCAATG | lmxll |
| Marker1785 | TCACATCTCTCTTGAGCTTGAGAGCTGCAGTGACCAGGCCXXXXXXXXXXCCAGTGGCGGATTTAGGAATTTTTCAGGGAAGGGGCAATG | lmxll |
| Marker1786 | GATTAGAGTAATCTCGGGGATAACGAAAGGTCGGTGCTATXXXXXXXXXXAAGAAAGAAGGGAAATGATAACATATAAGGCATAAATAGT | lmxll |
| Marker1786 | GATTAGAGTAATCTCGGGGATAACGAAAGGTCGGTGCTATXXXXXXXXXXAAGAATGAAGGGAAATGATAACATATAAGGCATAAATAGT | lmxll |
| Marker1790 | GGACTTATAAAAGTATATGCACGAAGTGTGTACATACGTTXXXXXXXXXXAATAAACATAGTAAGTGTAGTTTTCATAAGTCTGTTTTTC | nnxnp |
| Marker1790 | GGACTTATAAAAGTATATGCACGAAGTGTGTACATACGTTXXXXXXXXXXAATAAACGTAGTAAGTGTAGTTTTCATAAGTCTGTTTTTC | nnxnp |
| Marker1791 | GGATATTGAGGATGTGAACAAGATGAAAATGGCACTTTACXXXXXXXXXXCAACACCTAAAATAAGAAAGCTTTCATAAATGCAAACTGC | lmxll |
| Marker1791 | GGATATTGAGGATGTGAACAAGATGAAAATGGCACTTTACXXXXXXXXXXAAACACCTAAAATAAGAAAGCTTTCATAAATGCAAACTGC | lmxll |
| Marker1804 | ATAGTGTTTGAGTTTTTTTATAAATACATGTCTAGGATTGXXXXXXXXXXCAAGAAAAGTATCTACAGTTGACATGATGGACATGGACGG | lmxll |
| Marker1804 | ATAGTGTTTGAGTTTTTTTATAAATACATGTCTAAGATTGXXXXXXXXXXCAAGAAAAGTATCTACAGTTGACATGATGGACATGGACGG | lmxll |
| Marker1807 | ATATCAAACATGTTAGAAAGCATATAAAGATTGGAATCAAXXXXXXXXXXTATGCACGACAATATTCATGCCTTTTGATTTCCACTTTTA | lmxll |
| Marker1807 | ATATCAAACATGTTAGACAGCATATAAAGATTGGAATCAAXXXXXXXXXXTATGCACGACAATATTCATGCCTTTTGATTTCCACTTTTA | lmxll |
| Marker1808 | ATTGCAACGAAATTCTCGCCTTTGTCAAAGACATATCCTCXXXXXXXXXXAGTATCAACAAGTGACTGCATCACAAACCGCATGCTAATG | lmxll |
| Marker1808 | ATTGCAACGAAATTCTCGCCTTTGTCAAAGACATATCCTCXXXXXXXXXXAGTACCAACAAGTGACTGCATCACAAACCGCATGCTAATG | lmxll |
| Marker1809 | AGGGAATAAAGCAAGGAAATACAAGAACAAACACGAAAAAXXXXXXXXXXGACTTTCGTTTCTCAAAAGCGCTGAAAGGCGGAAATGCAA | lmxll |
| Marker1809 | AGGGAATAAAGCAAGGAAATACAAGAACAAACACGAAAAAXXXXXXXXXXGACTTTCATTTCTCAAAAGCGCTGAAAGGCGGAAATGCAA | lmxll |
| Marker1812 | TCTTTGTTTTCTTCCTCTGTGTGAAGCTGGCCATCAAGATXXXXXXXXXXTGGGTAGGGTCTAATCAAGACCATGAGAACTTGCCATCAA | lmxll |
| Marker1812 | TCTTTGTTTTCTTCCTCTGTGTGAAGCTGGCCATCAAGATXXXXXXXXXXTGGGTAGGGTATAATCAAGACCATGAGAACTTGCCATCAA | lmxll |
| Marker1822 | CCAACTCAACATATGTAATGAAAAATTGAAGGGTACTTTTXXXXXXXXXXTAGTTGTTGTCCTGTCTGCACTCTATTTCGATCCAATCGT | lmxll |
| Marker1822 | CCAACTCAACATATGTAATGAAAAATTGAAGGGTACTTTTXXXXXXXXXXTAGTTGTTGTCCTGTCTGCACTCTATTTCGAACCAATCGT | lmxll |
| Marker1824 | TGTAAGAAAATAAGAAAGCCCAACCACAAAATAAGCCGAAXXXXXXXXXXGAAATCCAGGAACCAAACTCTACGCTTCCGCCTAACCTTG | hkxhk |
| Marker1824 | TGTAAGAAAATAAGAAAGTCCAACCACAAAATAAGCCGAAXXXXXXXXXXGAAATCCAGGAACCAAACTCTACGCTTCCGCCTAACCTTG | hkxhk |
| Marker1826 | AGAAAAAGTTAGGTTTCCTGCCCCACGTCCTATTCTTCTTXXXXXXXXXXTCGAACCGTGTGCAGCCCTACAAAAAACCTAAGATATTAC | nnxnp |
| Marker1826 | AGAAAAAGTTAGGTTTCCTGCCCCACATCCTATTCTTCTTXXXXXXXXXXTCGAACCGTGTGCAGCCCTACAAAAAACCTAAGATATTAC | nnxnp |
| Marker1827 | CTCACATCCTATGCAGACTCGTTCCAAGTCTGGAATTATCXXXXXXXXXXCGATTGATCCTTGCTTTAGCTGCTCAGTATCATTGGTCTG | lmxll |
| Marker1827 | CTCACATCCTATGCAGACTCGTTCCAAGTCTGGAATTATCXXXXXXXXXXCGATTGATCCTTGCTTTAGCTGCTCAGTATCATTGGTTTG | lmxll |
| Marker1828 | AAATTTGAGAAATAGTTTGAGCCTTTTGTAGAATTTGCTAXXXXXXXXXXCAATTTGGTTGATTTTCACCATCAAAACGTCAAAACCATT | lmxll |
| Marker1828 | AAATTTGAGAAATAGTTTGAGCCTTTTGTATAATTTGCTAXXXXXXXXXXCAATTTGGTTGATTTTCACCATCAAAACGTCAAAACCATT | lmxll |
| Marker1835 | GACATGATGAGAACCTTGCCTTGCTTACATGGGATTCTGCXXXXXXXXXXTGCACATGCACTCATGGCGAAACTTCGATTGAATTCCACT | lmxll |
| Marker1835 | GACATGATGAGAACCTTGCCTTGCTTACATGGGATTCTGCXXXXXXXXXXTGCACATGCACTCATGGTGAAACTTCGATTGAATTTCACT | lmxll |
| Marker1837 | TAAAAGGTTCCAAGAACAAAGGACATTTATGTAAAAATAAXXXXXXXXXXATATACCACATTTGTCCCCCTGTAATCCGCCATAATTACA | lmxll |
| Marker1837 | TAAAAGGTTCCAAGAACAAAGGACATTTATGTAAAAATAAXXXXXXXXXXATATACCACATTTGTCTCCCTGTAATCCGCCATAATTACA | lmxll |
| Marker1838 | TTTCGTAACTGTTTTGGACTTTTGGTCCGCGTTGTGTTCTXXXXXXXXXXATTTTTAGTTTCCAATTTGCATTTGTGCATTTGTGGTGTG | hkxhk |
| Marker1838 | TTTCGTAACTGTTTTGGACTTTTGGTCCACGTTGTGTTCTXXXXXXXXXXATTTTTAGTTTCCAATTTGCATTTGTGCATTTGTGGTGTG | hkxhk |
| Marker1839 | CACGAAAACCGAACATCGAAAAGGAAATCGCAGATCTTCCXXXXXXXXXXATGTGAAAAGCTTACATGAACATGAACGTGCACATGTGCA | lmxll |
| Marker1839 | CACGAAAACCGAACATCGAAAAGGAAATCGCAGATCTTCCXXXXXXXXXXATGTGAAAAGCTTACATGAACATGAACGTGCACATATGCA | lmxll |
| Marker1846 | ACTCACGCATTTGTTTATTTATTTCAAGTCGTGGAGAGGAXXXXXXXXXXAGTGCGAAAGAAGTCCTTGTTTCAATGGGTGATTGTAACT | lmxll |
| Marker1846 | ACTCACGCATTTGTTTATTTATGTCAAGTCGTGGAGAGGAXXXXXXXXXXAGTGCGAAAGAAGTACTTGTTTCAATGGGTGATTGTAACT | lmxll |
| Marker1849 | GCTCGCCAAACGACAGACAGGATTGTAATCCATTTTCATTXXXXXXXXXXGAGTAATCCACTATAATCAAATGAGAGAATACAATAGTGT | hkxhk |
| Marker1849 | GCTCGCCAAACGACAGACAGGATTGTAATCCATTTTCATTXXXXXXXXXXGAGTAATCCACTATAATCAAAAGAGAGAATACAATAGTGT | hkxhk |
| Marker1853 | TTTGAGGAACTTGATATATCTATGAATCTTGAAAAATTTGXXXXXXXXXXCTCCTCAAGGGCATTTCCTTATTTCCACAGGTATAAATTG | lmxll |
| Marker1853 | TTTGAGGAGCTTGATATATCTATGAATCTTGAAAAATTTGXXXXXXXXXXCTCCTCAAGGGCATTTCCTTATTTCCACAGGTATAAATTG | lmxll |
| Marker1854 | AACCAGTAGCTGGATGATCTTCTAAAAATAAAAATAAAACXXXXXXXXXXAGTGACTTTTGGATTTTTACGAAAACCCAAAAGAAAGAAA | nnxnp |
| Marker1854 | AACCAGTAGCTGGATGATCTTCTAAAAATAAAAATAAAACXXXXXXXXXXAGTGACTTTCGGTTTTTTACGAAAACCCAAAAGAAAGAAA | nnxnp |
| Marker1862 | ATTTGCTGGCATCAAGAACCAGTTTCCTTACAGATGAAAAXXXXXXXXXXACCTGCATCATATTTGAGAAATATCACCTCAAATGCAACT | lmxll |
| Marker1862 | ATTTGCTGGCATCAAGAACCAGTTTCCTTACAGATGAAAAXXXXXXXXXXACCTGCACCATATTGGAGAAATATCACCTCAAATGCAACT | lmxll |
| Marker1868 | TGGTGAGGCCGTTTAGCTGAATTGAACAAATCAAGTAGTTXXXXXXXXXXCAATGTAGAATAAAAAATGCATGTATTTTGGGCCATGTTT | lmxll |
| Marker1868 | TGGTGAGGCCGTTTAGCTGAATTGAACAAATCAAGTAGTTXXXXXXXXXXCAATGTAGTATCAAGAATGCATGTATTTTGGGCCATGTTT | lmxll |
| Marker1872 | TAAACTGCAAATAATAACATTACGTTGCAGATTATTCATAXXXXXXXXXXGCACTGCTTTTTTTGGCATTTGGAATGGCTAAAAGTGTTT | lmxll |
| Marker1872 | TAAACTGCAAATAATAACATTACGTTTCAGATTATTCATAXXXXXXXXXXGCACTGCTTTTTTTGGCATTTGGAATGGCTAAAAGTGTTT | lmxll |
| Marker1883 | ATATAATTTGGCATCAAGATACACATTATAAATACAACAAXXXXXXXXXXCTTGTTGATTCTACTAAGCCCATCAATTGGCTCCATTCTC | lmxll |
| Marker1883 | ATATAATTTGGCATCAAGATACACGTTATAAATACAACAAXXXXXXXXXXCTTGTTGATTCTACTAAGCCCATCAATTGGCTCCATTCTC | lmxll |
| Marker1890 | GAAGAAACTGCCATGGAATGCTCGTATTAGAATGGCACTTXXXXXXXXXXTCCTTACAACAGCGTGGGTCATGAGACCAACATTGACAGT | nnxnp |
| Marker1890 | GAAGAAACTGCCATGGAATGCTCGTATTAGAATGGCACTTXXXXXXXXXXTCCTTACAACAGTGTGGGTCATGAGACCAACATTGACAGT | nnxnp |
| Marker1898 | GAACAATGCTACCCTGGCTGAAAAGGAATTCCAAAATCAAXXXXXXXXXXAGCAACTATTGGCCCTCATCACCATGCTGGTTACTAAGGA | nnxnp |
| Marker1898 | GAACAATGCTACCCTGGCTGAAAAGGAATTCCAAAATCAAXXXXXXXXXXAGCAACTATGGGCCCTCATCACCATGCTGGTTACTAAGGA | nnxnp |
| Marker1903 | ATCAGTGAGAAATAGTAGTATAGTGTTCCCAGCAAAAGAAXXXXXXXXXXGTACTCTTTCTTACCCTGACGTTTATTCCTCAAGAAGGTT | lmxll |
| Marker1903 | ATCAGTGAGAAATAGTAGTATAGTGTTCCCAGCAAAAGAAXXXXXXXXXXGTACTCTTTCTTACCCTGAGGTTTATTCCTCAAGAAGGTT | lmxll |
| Marker1904 | TGAGGTTTAGCAAGTTGTGCCTACGTCCCTGGAGCGGAGAXXXXXXXXXXGACACGTAGGATAATTTTCCACGTGTCATTGTGTTTTGGC | hkxhk |
| Marker1904 | TAAGGTTTAGCAAGTTGTGCCTACGTCCCCGGAGCGGAGAXXXXXXXXXXACACGTAGGATAATTTTTCCACGTGTCATTGTGTTTTGGC | hkxhk |
| Marker1906 | CATCAGGTAGAGGGAGAACCCCGATAACATTGCTATAAACXXXXXXXXXXGAAATTGAAACTGTATCAAATGCTCTATCCTTGAGTGAAC | nnxnp |
| Marker1906 | CATCAGGTAGAGGGAGAACCCCGATAGCATTGCTATAAACXXXXXXXXXXGAAATTGAAACTGTATCAAATGATCTATCCTTGAGTGAAC | nnxnp |
| Marker1915 | ACATCGAAAGATTGAACTTATGTTTATATATACACATATAXXXXXXXXXXTACAGTTTTTCCAATGGAGTAATCTTATTCAAATAGATGC | lmxll |
| Marker1915 | ACATCGAAAGATTGAACTTATGTTTATATATACACATATAXXXXXXXXXXTACAGTTTTTCCAATGGAGTAATCTTATTCAAATACATGC | lmxll |
| Marker1917 | TGATATTGTAAAGTGTTTGAATGATTTGTATGATGAGTATXXXXXXXXXXGATGAGGTGAAGATCTTCATGAAACTTGTTCCAATGGATG | nnxnp |
| Marker1917 | TGATATTGTAAAGTGTTTGAATGATTTGTATGATGAGTATXXXXXXXXXXGATGATGTGAAGATCTTCATGAAACTTGTTCCAATGGATG | nnxnp |
| Marker1924 | CCAAAAAAGTAAAAAAATGGTTATTAGCCATAGGGTTGAAXXXXXXXXXXATTGAAGATATGCAAGAGACACAGAACCAAGAATTCCAAT | lmxll |
| Marker1924 | CCAAAAAAGTAAAAAAATGGTTATTAGCCAAAGGGTTGAAXXXXXXXXXXATTGAAGATATGCAAGAGACACAGAACCAAGAATTCCAAT | lmxll |
| Marker1928 | GGACATAGGTTTGATGAACAACAGACTTCAATTTTCCAATXXXXXXXXXXAGCAAACTGGGGTTTATCCCAGTTGAACCACCTCCGATGG | hkxhk |
| Marker1928 | GGACATAGGTTTGATGAACAACAGACTTCAATTTTCCAATXXXXXXXXXXAGCAAACTGGGGTTTATCCCAGTTGAACCACCTCCAATGG | hkxhk |
| Marker1941 | TAACACTTACCATCTGCTTTTCGTATAAAAGCAACTTGTCXXXXXXXXXXCACAATATCCATGAAGAGAAAGGCTGCTCAATGGTAAACG | nnxnp |
| Marker1941 | TAACACTTACCATCTGCTTTTCGTATAAAAGCAACTTGTCXXXXXXXXXXCACAATATCCATGAAGAGAAAGGATGCTCAATGGTAAACG | nnxnp |
| Marker1943 | AACCAATGTATCTTCATTTCAAACTTGTCTATGAAAAGTGXXXXXXXXXXGCCTATGAGGTTGTTTTCTGATTTCTCTGAGAGTTCCACA | nnxnp |
| Marker1943 | AACCAATGTATCTTCATTTCAAACTTGTCTATGAAAAGTGXXXXXXXXXXGCCTATGAGGTTGTTTTCTGATTTCTCGGAGAGTTCCACA | nnxnp |
| Marker1947 | ATTTACAGACAATGTAAGCCAAAAGAAGCAAAACCCATCAXXXXXXXXXXCTTTAGACCAGAAATGGCAAAATTTTCCACCCCATTGTGC | lmxll |
| Marker1947 | ATTTGCAGACAATGTAAGCCAAAAGAAGCAAAACCCATCAXXXXXXXXXXCTTTAGACCAGAAATGGCAAAATTTTCCACCCCATTGTGC | lmxll |
| Marker1949 | TCAGTTGGAAGAAAATCATATGATCCAAATAAATATATATXXXXXXXXXXTATGTTGAATTCGCGATAGCTAATTTTCTCTCTGCCCTTT | lmxll |
| Marker1949 | TCAGTTGGAAGAAAATCATATGATCCAAATAAATATATATXXXXXXXXXXTATGTTGAATTCGCGATAGCTAATTTTCTCTCTTCCCTTT | lmxll |
| Marker1962 | GCTGTGGCTTTTGTTTTCACAGTATTTTCTTATTTTTCTCXXXXXXXXXXAGAGCCTCTGAACTGAGAGTAACAGTAGAGGCGTATAATA | nnxnp |
| Marker1962 | GCTGTGGCTTTTGTTTTCACAGTATTTTCTTATTTTTCTCXXXXXXXXXXAAAGCCTCTGAACTGAGAGTAACAGTAGAGGCGTATAATA | nnxnp |
| Marker1988 | AGAGGCCTGTGGCCAAAATGATGTGATGTTTGGTTGCATGXXXXXXXXXXTCCTCATATGGACCTTCATATTAGCTACATGATCATTTCA | lmxll |
| Marker1988 | AGAGGCCTGTGGCAAAAATGATGTGATGTTTGGTTGCATGXXXXXXXXXXTCCTCATATGGACCTTCATATTAGCTACATGATCATTTCA | lmxll |
| Marker1995 | ACATGAATTGAGAAAGCTGCGACAAACTCACAAGGCTATCXXXXXXXXXXGAAAAATTGGTAGAAAAATGTGAGTCTGTAATCTCACTTT | lmxll |
| Marker1995 | ACATGAATTGAGAAAGCTGCGACAAACTTACAAGGCTATCXXXXXXXXXXGAAAAATTGGTAGAAAAATGTGAGTCTGTAATCTCACTTT | lmxll |
| Marker2004 | GCCAAAGCCAACTCATTAGTCACCTGCCAGTCCTTGGCCGXXXXXXXXXXTTTACCAGTGAAGCGAGTGTCACGATCACTGATAATACTC | lmxll |
| Marker2004 | GCCAAAGCCAACTCATTAGTCACCTGCCAGTCCTTGGCCGXXXXXXXXXXTTTACCAATGAAGCGAGTGTCACGATCACTGATAATACTC | lmxll |
| Marker2013 | GAGGTTGGTCTCGGCATTCTCAAGAACATCAGTGATAATGXXXXXXXXXXTGATTTCTGAATCAGCAACTAAATTGTTGTGTCTTTATGG | lmxll |
| Marker2013 | GAGGTTGGTCTCGGCATTCTCAAGAACATCAGTGATAATGXXXXXXXXXXTGATTTCTGAATAAGCAACTAAATTGTTGTGTCTTTATGG | lmxll |
| Marker2014 | GTCTTCTGCTAATATCACCTCCTCAATATCTGTGTCTCTAXXXXXXXXXXCCCCTCTCACACTTCAGGTCCCCAAAAATCTCTCTCTCTC | nnxnp |
| Marker2014 | GTCTTCTGCTAATATCACCTCCTCAATATCTGTGTCTCTAXXXXXXXXXXCCTCTCACACTTCAGGTCCCCAAAAATCTCTCTCTCTCTC | nnxnp |
| Marker2015 | ACAGTTCGGATCATCGGATCGCATCAATGGTTGGCCTAACXXXXXXXXXXACAGCACCAAAGTCCTGTATAAGAACACTTCAACATCATA | nnxnp |
| Marker2015 | ACAGTTCGGATCATCGGATCGCATCAATGGTTGGCCTAACXXXXXXXXXXACAGCACCAAAGTCCTGTATAAGAACACTTCACCATCATA | nnxnp |
| Marker2021 | GATGTAATTGTGCCTAAGCCTACACACGGGAGTTATCTGAXXXXXXXXXXATTCAATACGCAGTTCTTAGAGTTCTTCCAAATCGAGTTA | lmxll |
| Marker2021 | GATGTAACTGTGCCTAAGCCTACACACGGGAGTTATCTGAXXXXXXXXXXATTCAATATGCAGTTCTTAGAGTTCTTCCAAATCGAGTTA | lmxll |
| Marker2026 | ATTGAGAAATAATCCAAGATATTGTGGTTTATAATTCTTCXXXXXXXXXXTCAGAGAAGTCATTGAAAAGTCAAATAAAGAGCCTTTGGG | hkxhk |
| Marker2026 | ATTGAGAAATAATCCAAGATATTATGGTTTATAATTCTTCXXXXXXXXXXTCAGAGAAGTCATTGAAAAGTCAAATAAAGAGCCTTTGGG | hkxhk |
| Marker2029 | AAAACTGAAGAAACAGAGAAAAAGAGAGAGAGAGAGAGAGXXXXXXXXXXCGTGCATTTCGTGCATTCTCCAACCTAATCATCGATCTCG | nnxnp |
| Marker2029 | AAAACTGAAGAAACAGAGAAAAAGAGAGAGAGAGAGAGAGXXXXXXXXXXCGTGCGTTTCGTGCATTCTCCAACCTAATCATCGATCTCG | nnxnp |
| Marker2034 | TGGTTTCAGAATCATGTTTCTCTAATTCTTCATCGTCCATXXXXXXXXXXCTTGGTAATAAGGCAGACGTGCAAACCTTGTTTCATTTTC | nnxnp |
| Marker2034 | TGGTTTCAGAATCATGTTTCTCTAATTCTTCATCATCCATXXXXXXXXXXCTTGGTAATAAGGCAGACGTGCAAACCTTGTTTCATTTTC | nnxnp |
| Marker2036 | AATTGCTGCGTTTTGCCTCATACAGAGTCTTCCAGGATGTXXXXXXXXXXAGTCATTTCGTAGGTGATGTTACACACTGCCAAGCAGTCC | hkxhk |
| Marker2036 | ACTTGCTGCGTTTTGCCTCATACAGAGTCTTCCAGGATGTXXXXXXXXXXAGTCATTTCGTAGGTGATGTTACACACTGCCAAGCAGTCC | hkxhk |
| Marker2038 | TAATTTTTACTATAACATACAAATGAGTGTAAATGGACAAXXXXXXXXXXACCAAGGTGGTTGGTGTGAGTGGTTCATCACTCTCATTCC | lmxll |
| Marker2038 | ATAATTTTTACTATAACATACAAATGAGTGTAAATGGACAXXXXXXXXXXACCAAGGTGGTTGGTGTGAGTGGTTCATCACTCTCATTCC | lmxll |
| Marker2045 | CATACTATATATATCGCCTTGGCATGTAGCTCTCTCCTTGXXXXXXXXXXGACAGACAGCAACTAAGGTTGACTTATAAAGTTCTTACAA | lmxll |
| Marker2045 | CATACTATATATATCGCCTTGGCATGTAGCTCTCTCCTTGXXXXXXXXXXGACAGACAGGAACTAAGGTTGACTTATAAAGTTCTTACAA | lmxll |
| Marker2046 | ATTTGATTTTACATCGTCTTTGACGGAGGTTGCTGCAAATXXXXXXXXXXTAGTATTTTAGATGTCTTATTGATATCTTTGATTGTACCG | efxeg |
| Marker2046 | ATTTGATTTTACATCGTCTTTGACGGAGGTTGCTGCAAATXXXXXXXXXXTGGTATTTTAGATGTCTTGTTGATGTCTTTGATTGTACCG | efxeg |
| Marker2056 | AAAGCAACTCAAAACAACTGGTGAGTGTGACGTGATCTTTXXXXXXXXXXCAAGTTTATTTGTATTCTCTTCCAAGAACGAAACAATATG | nnxnp |
| Marker2056 | AAAGCAACTCAAAACAACTGGTGAGTGTGACGTGATTTTTXXXXXXXXXXCAAGTTTATTTGTATTCTCTTCCAAGAACGAAACAATATG | nnxnp |
| Marker2060 | AGTTCAAATTGCATCTCATGGTCCTGCTCCTCCCAAATCCXXXXXXXXXXGAAAAGGCTGCAACTAAATTCTCCTCTCTTTCCATCTTGT | hkxhk |
| Marker2060 | AGTTCAAATTGCTTCTCATGGTCCTGCTCCTCCCAAATCCXXXXXXXXXXGAAAAGGCTGCAACTAAATTCTCCTCTCTTTCCATCTTGT | hkxhk |
| Marker2063 | CCAGAAATTTTTCCTTCACTCCCCTTAGTGGGTTGAACGGXXXXXXXXXXATTGAAAACTGGTAAGGCAATTGATCCACGGACTTGATGA | lmxll |
| Marker2063 | CCAGAAACTTTTCCTTCACTCCCCTTAGTGGGTTGAACGGXXXXXXXXXXATTGAAAACTGGTAAGGCAATTGATCCACGGACTTGATGA | lmxll |
| Marker2067 | TGTCAAATGTTTATGCCCAAAATGAAAGGGCTTAGAAGGCXXXXXXXXXXGTGCTATAGAACCACGGATATTTCCTAAACCCTTTCCAAT | nnxnp |
| Marker2067 | TGTCAAATGTGTGTGCCCAAAATGAAAGGGCTTAGAAGGCXXXXXXXXXXGTGCTATAGAACCACGGATATTTCCTAAACCCTTTCCAAT | nnxnp |
| Marker2071 | ATTACTTTGATGCCCAATTGAGCAAGGTCTAAAATATCGAXXXXXXXXXXCAATTTGCCCCCTCTGACTGCCAAACCATTAGTGAATCTG | hkxhk |
| Marker2071 | ATTACTTTGATGCCCAATTGAGTAAGGTCTAAAATATCGAXXXXXXXXXXCAATTTGCCCCCTCTGATTGCCAAACCGTTAGTGAATCTG | hkxhk |
| Marker2072 | TCTCCATCTAATTTTTCCGTGATTTCTGCCTTGGAATGTTXXXXXXXXXXGACAAATGCAACCTTTTTCCATTTCTACTTATCAAACACA | nnxnp |
| Marker2072 | TCTCCCTCTAATTTTTCCGTGATTTCTCCCTTGGAATGTTXXXXXXXXXXGACAAATGCAACCTTTTTCCATTTCTACTTATCAAACACA | nnxnp |
| Marker2081 | AAGGCTGATACCATGTAAAGGAGAATTTCCCGATGAATATXXXXXXXXXXAAATCATTCTTTGCATGTCCTGGAAGCAGATGCATATAAT | lmxll |
| Marker2081 | AAGGCTGATGCCATGTAAAGGAGAATTTCCCGATGAATATXXXXXXXXXXAAATCATTCTTTGCATGTCCTGGAAGCAGATGCATATAAT | lmxll |
| Marker2084 | TCTACTCAAAGCCCATGTGTAATTGTCTTCATTCTTAGCAXXXXXXXXXXAATTGTTTTCATTGTGGTTTCATTGAGTCCATCTTTTTGT | hkxhk |
| Marker2084 | TCTACTCAAAGCCCATGTGTAATTGTCTTCATTCTTAGCAXXXXXXXXXXAATTGTTTTCATTGTTGTTTCATTGAGTCCATCTTTTTGT | hkxhk |
| Marker2088 | ATGGGACACAATGTGACTCAGCTATCACTCGTGCCACTGCXXXXXXXXXXTAGGGCTTTTATATGCATATAAGACAAGTCCAAAATATAA | nnxnp |
| Marker2088 | ATGGGACACAATGTGACTCAGCTATCACTCGTGCCACTGCXXXXXXXXXXTAGGGCTTTTATATGCATATGAGACAAGTCCAAAATATAA | nnxnp |
| Marker2093 | GACATTTAGTGCATGCATGTTTTAGATGCTAATTGACAAAXXXXXXXXXXGCACACACCTGCATTTATGTTTGTGAGGAGTGCTTTTCTT | lmxll |
| Marker2093 | GACATTTAGTGCATGCATGTTTTAGATGCTAATTGACAAAXXXXXXXXXXGCACACACCTGCATTTATGTTTGTGAGGTGTGCTTTTCTT | lmxll |
| Marker2103 | AGCTTTTGGTGAGAAATTGAGAAATCACATACCGTTTCCTXXXXXXXXXXAGGTTCAAGCTTCTCAACGGGTGCCAACGTTTCCTGAAAG | lmxll |
| Marker2103 | AGCTTTTGGTGAGAAATTGAGAAATCACATACCGTTTCCTXXXXXXXXXXAGGTTCAAGCTTCTCAACGGATGCCAACGTTTCCTGAAAG | lmxll |
| Marker2107 | GTTGCCAAACAAGGGAATTGTCAATTTCTTCTCGGAAATTXXXXXXXXXXATTGAATTGCTCTCTTTCACAACAATCACTCATACACGCG | nnxnp |
| Marker2107 | GTTGCCAAACAAGGGAATTGTCAATTTCTTCTCGGAAATTXXXXXXXXXXATTGAATTGCTCTCTTCCACAACAATCACTCATACACGCG | nnxnp |
| Marker2113 | AAAGAGTGGAATATTCAAAACTTCGTTCACAAGACCGCAAXXXXXXXXXXTTCTTCTTTTGCCCATGTGATGTATGTCTTTTTACCTTGT | lmxll |
| Marker2113 | AAAGAGTGGAATATTCAAAACTTCGTTCACAAGACCGCAAXXXXXXXXXXTTCTTCTTTTGCCCATGTGATGTATGTCTTTTTACCTTGC | lmxll |
| Marker2116 | GCATCTCCAAAAAGTAGTGGGAAAGTAGGAAGGGCTCGGAXXXXXXXXXXTGTACGTTGCCTCTTGCCATTATTGAAAGCTCTGGTGAAT | lmxll |
| Marker2116 | GCATCTCCAAAAAGTTGTGGGAAAGTAGGAAGGGCTCGGAXXXXXXXXXXTGTACGTTGCCTCTTGCCATTATTGAAAGCTCTGGTGAAT | lmxll |
| Marker2117 | CCAGCATAAAACAGCCACCTCTCAACTCTCTCTCTCCTTTXXXXXXXXXXACTGGTTAGGAAAAGTTTGGTTTCTTGGATTTGAATGATT | hkxhk |
| Marker2117 | CCAGCATAAAACAGCCACCTCTCAACTCTCTCTCTCTTTTXXXXXXXXXXACTGGTTAGGAAAAGTTTGGTTTCTTGGATTTGAATGATT | hkxhk |
| Marker2118 | AGAATGTCTTTATCTTCCAAGTTGCGTAAAGAACCTAATGXXXXXXXXXXTTTTTTAGGAAAGTGGGAAAGAGTCACTGAGCTTCTTGAA | nnxnp |
| Marker2118 | AGAATGTCTTTATCTTCCAAGTTGCGTAAAGAACCTAATGXXXXXXXXXXTTTTTTATGAAAGTGGGAAAGAGTCACTGAGCTTCTTGAA | nnxnp |
| Marker2125 | AACAGAAACAAGTTTGCGACACTATTGAGTCCTGACAGCAXXXXXXXXXXGCTTGGGTAAAACATAGACATAGACATAGACACGAAGATA | lmxll |
| Marker2125 | AACAGAAACAAGTTTGCAACACTATTGAGTCCTGACAGCAXXXXXXXXXXGCTTGGGTAAAACATAGACATAGACATAGACACGAAGATA | lmxll |
| Marker2135 | ATTTGCTCCGCGTTTCCTCATACAAAGTCTTCCAGGATGTXXXXXXXXXXGCCCATGAAATCGGTGATGTTACAAACTGCCAAGCAGTTT | lmxll |
| Marker2135 | ATTTGCTCCGCGTTTCCTCATACAGAGTCTTCCAGGATGTXXXXXXXXXXGCCCATGAAATCGGTGATGTTACAAACTGCCAAGCAGTTT | lmxll |
| Marker2140 | ACCCTAATTATTTTGAGTTGGCATCAGAGCGGGTTCCGATXXXXXXXXXXGCTACTACATAAGGCAAATTGGGTTACTTGACTGGTGTTG | nnxnp |
| Marker2140 | ACCCTAATTATTTTGAGTTGGCATCAGAGCGGGTTCCGATXXXXXXXXXXGCTACTACACAAGGCAAATTGGGTTACTTGACTGGTGTTG | nnxnp |
| Marker2141 | GATGGATGGTTCCGAGCAACGGTCTTGTGCAAAGAATATAXXXXXXXXXXGTAAATGTAAATAGAATGTAAAATTGTTATTCCCTAAGAA | nnxnp |
| Marker2141 | GATGGATGGTTCCGAGCAACGGTCTTGTGCAAAGAATATAXXXXXXXXXXGTAAATGTAAATAAAATGTAAAATTGTTATTCCCTAAGAA | nnxnp |
| Marker2142 | ATACCTGATAGTGATCCATTCAAGAATATAACTAATCAGAXXXXXXXXXXTAGATTATCTATAACATGGTGATAAAGGACAATTATTTTA | nnxnp |
| Marker2142 | ATACCTGATAGTGATCCATTCAAGAATATAACTAATCAGAXXXXXXXXXXCAGATTATCTATAACATGGTGATAAAGGACAATTATTTTA | nnxnp |
| Marker2145 | TAGGAAATAGACTTGGAACATAAGCAAACTGCAGGTTTCTXXXXXXXXXXAGCTTACACTTGTCATAAAAGAGATAAAATGGAAAACTAA | lmxll |
| Marker2145 | TAGGAAATAGACTTGGAACATAAGCAAGCTACTGCAGGTTXXXXXXXXXXAGCTTACACTTGTCATAAAAGAGATAAAATGGAAAACTAA | lmxll |
| Marker2151 | TCTAATTGAACTTGGGAACTTATTTGCTTGAGTTATTTCCXXXXXXXXXXTTCTTTTCCTTGGAACTTTTCTTCCTCTGCAATCATCAAC | efxeg |
| Marker2151 | TCTAATTGAACTTGGGAACTTATTTGCTTGAGTTATTTCCXXXXXXXXXXTGCTTTTCCTTGGAACTTTTCTTCCTCTGCAATCATCAAC | efxeg |
| Marker2154 | TACATTATGGAAGTTGTTATTCCAAACCAACTGAGGCTAAXXXXXXXXXXACCAATATTGTTCACTGGGCAGCACCCAACATTCACAACC | lmxll |
| Marker2154 | TACATTATGGAAGTTGTTATTCCAAACCAACCGAGGCTAAXXXXXXXXXXACCAATATTGTTCACTGGGCAGCACCCAACATTCACAACC | lmxll |
| Marker2158 | CCGGAAAGTTCATTGGGAGATTGTGATCATAGCAATTCAGXXXXXXXXXXGTAGAGAGCACGCAAGTATGAGAAGGACGTTGTATGCCCC | lmxll |
| Marker2158 | CCGGAAAGTTCATTGGGAGATTGTGATCATGGCAATTCAGXXXXXXXXXXGTAGAGAGCACGCAAGTATGAGAAGGACGTTGTATGCCCC | lmxll |
| Marker2162 | TGTTAGCATGCAGTAGTTGACTCCGATATCTTTTTCGAAAXXXXXXXXXXCAATTTTTACTTGTAATTTCAGTTCTTGTTACCCATAATA | lmxll |
| Marker2162 | TGTTAGCATGCAGTAGTTGACTCCGATATCTTTTTCGAAAXXXXXXXXXXCAATTTTTACTTGTAATTTCAGTTATTGTTACCCATAATA | lmxll |
| Marker2163 | ATTTTAGTTGCCCGCATCAGGGCTCATCCTTTTCTTTTGCXXXXXXXXXXGGCATTGTGCCTTCAATAAATGCAGGGCTGGCAAACATAC | nnxnp |
| Marker2163 | ATTTTAGTTGCCCGCATCAGGGCTCATCCTTTTCTTTTGTXXXXXXXXXXGGCATTGTGCCTTCAATAAATGCAGGGCTGGCAAACATAC | nnxnp |
| Marker2170 | TCTTGATGAGAATATTGCCAATATTGTGAAACATTCACATXXXXXXXXXXAAATTTCTTCTAATTAGAATATTCTTTCTTTCTAATGGGT | lmxll |
| Marker2170 | TCTTGATGAGAATATTGCCAATATTGTGAAACATTCACATXXXXXXXXXXTAGCTTCTTCTAATTAGAATATTCTTTCTTTCTAATGGGT | lmxll |
| Marker2172 | AGTTTGTGTTGGAATAAATTTATAGGGGAGTATGGAGTTTXXXXXXXXXXGAATTAGTGAAAAACGGTCAACGAAAGCTTTTCTTGTCGC | lmxll |
| Marker2172 | AGTTTGTGTTGGAATAAATTTATAGGGGAGTATGGAGTTTXXXXXXXXXXGAATTAGTGAAAAACAGTCAACGAAAGCTTTTCTTGTCGC | lmxll |
| Marker2175 | TTCCTGTTACATGTGTACTGGATCTGGCTACTTTTATCAAXXXXXXXXXXATGCTTCTGCTGTTGCTGGGGATTTTGGTATTCAATAAAT | lmxll |
| Marker2175 | TTCCTGTTACATGTGTACTGGATCTGGCTGCTTTTATCAAXXXXXXXXXXATGCTTCTGCTGTTGCTGGGGATTTTGGTATTCAATAAAT | lmxll |
| Marker2180 | CGCACATCCCCTCGGCCCGGAACTCCAGGTCGTGAGATTTXXXXXXXXXXGAGAGGGTGAGGACGAAGATTGCCAAAGAAGATCGGTTCT | nnxnp |
| Marker2180 | CGCACATCCCCTCGGCCCGGAACTCCAGGTCGTGAGATTTXXXXXXXXXXGAGAGGGTGAGGACGAAGATTGCCGAAGAAGATCGGTTCT | nnxnp |
| Marker2210 | GCAAAATGTGAATGAGATGGAAAGGAAATTAGGTACTGAAXXXXXXXXXXAAAAGTTGGCTTCTGGGAAAAGCCATCCCCTCTTTTCCTT | nnxnp |
| Marker2210 | GCAAAATGTGAATGAGATGGAAAGGAAATTAGGTAGTGAAXXXXXXXXXXAAAAGTTGGCTTCTGGGAAAAGCCATCCCCTCTTTTCCTT | nnxnp |
| Marker2211 | ACAGCTGGATGAGTAATATTACTGTGGAATGGATCACATCXXXXXXXXXXTTTTTCTCACAAACGGCCATAGCTTTCCAAGTTTCCAATG | lmxll |
| Marker2211 | AACAGCTGGATGAGTAATATTACTGTGGAATGGATCACATXXXXXXXXXXTTTTTCTCACAAACGGCCATAGCTTTCCAAGTTTCCAATG | lmxll |
| Marker2216 | AATCATTACATGCCCAACCAACTTTCCAAAATGTTAGCTTXXXXXXXXXXTGAAAATGTAAATGAAAGAAAAGAACTACCATGCATTTTC | lmxll |
| Marker2216 | AATCATTTCATGCCCAACCAACTTTCCAAAATGTTAGCTTXXXXXXXXXXTGAAAATGTAAATGAAAGAAAAGAACTACCATGCATTTTC | lmxll |
| Marker2223 | TTTGTGTTGGACAAAACAAAAACTCAAAGTTGAAGATGTTXXXXXXXXXXGCCTCCCATGGTGCCACATTACCCTTCATCTTTTGGTTTA | lmxll |
| Marker2223 | TTTGTGTTGGACAAAACAAAAACTCAAAGTTGAAGATGTTXXXXXXXXXXGCCTCCCATGGTGCCACATTACCCTTCATCTTCTGGTTTA | lmxll |
| Marker2224 | CCTACTGTAGAAGAAAGGTGGGAACACTTCACGAGAACACXXXXXXXXXXAGAAACCCATCAGCTTTCGCCTGCAAAACAATAATACGTA | lmxll |
| Marker2224 | CCTACTGTAGAAGAAAGGTGGGAACACTTCACGAGAACACXXXXXXXXXXAGAAACCCATCAGCTTTTGCCTGCAAAACAATAATACGTA | lmxll |
| Marker2234 | GAAATATATGATTAGACAAAGGTCTTGGTCAAATAAAAATXXXXXXXXXXCTTTCATTATCATACCCAATATAAGACCCTAAATTATTTA | lmxll |
| Marker2234 | GAAATATATGATTAGACAAAGGTCTTGGTCAAATAAAAATXXXXXXXXXXTTTCATTATCATACCCAATATAAGACCCTAAATTATTTAT | lmxll |
| Marker2236 | CATGTTCACCCTTTTTTTTCTAATGTTTTTTTTCCCTAAAXXXXXXXXXXGACCTGAAAAAAAAGGAGTTGATTTGGGGAGCCCAAGAAC | lmxll |
| Marker2236 | CATGTTCACCCTTTTTTTCTAATGTTTTTTTTCCCTAAATXXXXXXXXXXGACCTGAAAAAAAAGGAGTTGATTTGGGGAGCCTAAGAAC | lmxll |
| Marker2237 | TAATAGCATGTGCTCTTATCTTCTCAGCTGCATCTGAATTXXXXXXXXXXTCCGGCCTCCATGATGATTGTTTTTATCGTATATCTTGTT | hkxhk |
| Marker2237 | TAATAGCATGTGCTCTTATCCTCTCAGCTGCATCTGAATTXXXXXXXXXXTCCGGCCTCCATGATGATTGTTTTTATCGTATATCTTGTT | hkxhk |
| Marker2238 | TTTGCTAAATTGAGGAGTATCCATAGCATAATGTGTTTGTXXXXXXXXXXAGTTGGGTTGAAGGTCAGTTGTAAATCCATCTTCTGTGTT | nnxnp |
| Marker2238 | TTTGCTAAATTGAGGCGTATCCATAGCATAATGTGTTTGTXXXXXXXXXXAGTTGGGTTGAAGGTCAGTTGTAAATCCATCTTCTGTGTT | nnxnp |
| Marker2241 | CACATTCCATATGCTGCGATCTCCTGGCTGCTTACACTTAXXXXXXXXXXCTCTCTTTTGGGTCGAGAATGATCCGTGTGGTGTGCAGAC | hkxhk |
| Marker2241 | CACATTCCATATGCTGCGATCTCCCGGCTGCTTACACTTAXXXXXXXXXXCTCTCTTTTGGGTCGAGAATGATCCGTGTGGTGTGCAAAC | hkxhk |
| Marker2250 | ATGTGTCAAATATCTCCAAGAAATTAGAGATATAGCTATTXXXXXXXXXXACCAAAAACAAAAACAAAAAAAAACAAAAAAAAATTCAAA | nnxnp |
| Marker2250 | ATGTGTCAAATATCTCCAAGAAATTAGAGATATAGCTATTXXXXXXXXXXCCAAAAACAAAAACAAAAAAAAAACAAAAAAAAATTCAAA | nnxnp |
| Marker2253 | TCAAAACATTTTCTTATTGCATATAACCAAAGCTAACAAAXXXXXXXXXXGTGAGGCCTGAAAATGGCAAAGCCAAAAACACCTCTTGAC | nnxnp |
| Marker2253 | TAAAAACATTTTCTTATTGCATATAACCAAAGCTAACAAAXXXXXXXXXXGTGAGGCCTGAAAATGGCAAAGCCAAAAACACCTCTTGAC | nnxnp |
| Marker2265 | GAGCTCTTGCTGTTGACTATATAGTAGTATAAAAGTCAAGXXXXXXXXXXTGTACGAGTGGAGGGTCAGATATGGTACAAACACATTTTA | nnxnp |
| Marker2265 | GAGCTCTTGCTGTTGACTATATAGTAGTATAAAAGTCAAGXXXXXXXXXXTGTATGAGTGGAGGGTCAGATATGGTACAAACAAATTTTG | nnxnp |
| Marker2270 | CCAAGTCAGCAGCAGAAGAGGGGGCAATAGCAGATGAGATXXXXXXXXXXGTTGGATCACAATAGTTGGGCCCAGTTCGACCTCCATCCC | lmxll |
| Marker2270 | CCAAGTCAGCAGCAGAAGAGGGGGCAATAGCAGATGAGATXXXXXXXXXXGTTGGATCACGATAGTTGGGCCCAGTTCGACCTCCATCCC | lmxll |
| Marker2271 | AGTGCAATGAGATTTTGCGAGTTCACCAGTAGTGGCATTGXXXXXXXXXXACCAGCTCAAAATTTTAGTACAGATGTTGCTCCTGCTCGT | lmxll |
| Marker2271 | AGTGCAATGAGATTTTGCGAGTTCACCAGTAGTGGCAATGXXXXXXXXXXACCAGCTCAAAATTTTAGTACAGATGTTGCTCCTGCTCGT | lmxll |
| Marker2273 | TATATTTCATAATAACCCTTGCTTCAGAATCTGTTGACATXXXXXXXXXXGAAGCAATGCCCATCTGTGCAGGATAAAAACACAGAACTG | nnxnp |
| Marker2273 | TATAGTTCATAATAACCCTTGCTTCAGAATCTGTTGACATXXXXXXXXXXGAAGCAATGCCCATCTGTGCAGGATAAAAACACAGAACTG | nnxnp |
| Marker2277 | ATCCTCCTCAAGAAAATATGCTATATATGACCCCAAAAAAXXXXXXXXXXAAGCCTAAGTAACTGCAATGGGCTTGTGGTCTATATATGC | nnxnp |
| Marker2277 | ATCCTCCTCAAGAAAATATGCTATATATGACCCAAAAAAAXXXXXXXXXXAAGCCTAAGTAACTGCAATGGGCTTGTGGTCTATATATGC | nnxnp |
| Marker2278 | GTGCAACACTGCAATATCTCAAGCAAATCAAATAGTTGGTXXXXXXXXXXTGGAGTATGTGCAGTCAGTAAGTGATAAATACAATCTTTC | lmxll |
| Marker2278 | GTGCAACACAGCAATATCTCAAGCAAATCAAATAGTTGGCXXXXXXXXXXTGGAGTATGTGCAGTCAGTAAGTGATAAATACAATCTTTC | lmxll |
| Marker2285 | CAGATAAAAAAAAGTTCTACAACAGCCTGTTTATCCCAACXXXXXXXXXXTATTTATCCTCAAAGTGACATCTTTGCCTTTGCCCTGTTA | lmxll |
| Marker2285 | CAGATAAAAAAAAGTTCTACAACAGCCTGTTTATCCCAACXXXXXXXXXXTATTTATCCTCAAATTGACATCTTTGCCTTTGCCCTGTTA | lmxll |
| Marker2287 | TAATAAAGGTGTCGTTTGGGGACGAAGAAGGAAGTGCCGTXXXXXXXXXXTTCATTTTGCCAACCTTGACCACATAATATCAACGCACCA | lmxll |
| Marker2287 | TAATAAAGGTGTCGTTTGGGGACGAAGAAGGAAGTGCCGTXXXXXXXXXXTTCATGTTGCCAACCTTGACCACATAATATCAACGCACCA | lmxll |
| Marker2289 | GTTTTAGTGGAGTAAATCATAGTTCAAGCTGTGTTATGTTXXXXXXXXXXTCTGAAAGATCATCATTTCTTGTCAATCTGGTGGATGGAA | lmxll |
| Marker2289 | GTTTTAGTGGAGTAAATCATAGTTCAAGCTGTGTTATGTTXXXXXXXXXXTCTGAAAGATCATCATAGCTTGTCAATCTGGTGGATGGAA | lmxll |
| Marker2294 | ATTCAGCACCACGAAATCGAAGTTCAACCCCACAACACTAXXXXXXXXXXAGACAGGAAGGAGAAGAAAGGGGCTTGCTTGCAATGACTT | nnxnp |
| Marker2294 | ATTCAGCACCACGAAATCGAAGTTCAACCCCACAACACTAXXXXXXXXXXAGACAGGAAGGAAAAGAAAGGGGCTTGCTTGCAATGACTT | nnxnp |
| Marker2296 | CAACATAGCCACAAAACATCTATAATGCCTCAGGTCTAAGXXXXXXXXXXCTATGGTTGTTCTACATATTGAAAGATAATATAGAATAAT | hkxhk |
| Marker2296 | CAACATAGCCACAAAACATCTATGATGCCTCAGGTCTAAGXXXXXXXXXXCTATGGTTGTTCTACATATTGAAAGATAATATAGAATAAT | hkxhk |
| Marker2303 | GTACAAGCGATAATCTAAACTATAAGAGAGGGGAATTTCTXXXXXXXXXXGGTCCTTTGGCGTCGGTTGAAAAGGTATTCTGTTAGTCAA | nnxnp |
| Marker2303 | GTACAAGCGATAATCTAAACTATAAGGGAGGGGAATTTCTXXXXXXXXXXGGTCCTTTGGCGTCGGTTGAAAAGGTATTCTGTTAGTCAA | nnxnp |
| Marker2310 | TCGGACTTGGTGGGTTGGTTTGGTTCGGTTTGGGTTTTTTXXXXXXXXXXAGAGAGAAGACAAGATGCATGCGTGTGTTGAGGTTTTTTT | efxeg |
| Marker2310 | TCGGACTTGGTGGGTTGGTTTGGTTCGGTTTGGGTTTTTTXXXXXXXXXXGAGAGAAGACAAGATGCATGCGTGTGTTGAGGTTTTTTTT | efxeg |
| Marker2321 | ATCATAACGCTACAAAGCAGAAAGATCAAGGTGCAATGAGXXXXXXXXXXTACTATCAACTTTGCGCGACGAATATTTGCGCGACGAAAG | hkxhk |
| Marker2321 | ATCATAACGCTACAAAGCAGAAAGATCAAGGTGCAATGAGXXXXXXXXXXTACTATCAACTTTGCGCGACGAATATTTGAGCGACGAAAG | hkxhk |
| Marker2325 | AAAAATAATAATAAAAGCACATTTGTATTTCTCTCCTTCGXXXXXXXXXXCCAGCCTAAGACCCTCCGATGCCGAAGTAAGTTTGTTGTT | nnxnp |
| Marker2325 | AAAAAAAATAATAAAAGCACATTTGTATTTCTCTCCTTCGXXXXXXXXXXCCAGCCTAAGACCCTCCGATGTCGAAGTAAGTTTGTTGTT | nnxnp |
| Marker2335 | CCAAATGACTCCTCCCACCATATTATGATGTTGCCTAAGTXXXXXXXXXXCCAAATGACTCCTTCCACCATATTATGTTGTTGCCCAAGT | lmxll |
| Marker2335 | CCAAATGACTCCTCCCACCATAATATGATGTTGCCTAAGTXXXXXXXXXXCCAAATGACTCCTTCCACCATATTATGTTGTTGCCCAACT | lmxll |
| Marker2341 | TGCTTCATAACAGCTGGACAACGTTTGAGTGTTTGCTGCAXXXXXXXXXXTGACTTTTTTTGTTTTTTGTTTTCTTTTCAACGTTTATTG | lmxll |
| Marker2341 | TGCTTCATAACAGCTGGACAACGTTTGAGTGTTTGCTGCAXXXXXXXXXXATGACTTTTTTTGCTTTTTTTTTCTTTTCAACGTTTATTG | lmxll |
| Marker2355 | CAACGCCTCCTACGAAACGAGTTACGAGGAAAGAAGTTGCXXXXXXXXXXGTTGAAGCGGAGAAGAAAAAGGGGATTGACATGTCCAAGT | lmxll |
| Marker2355 | CAACGCCTCCTACGAAACGAGTTACGAGGAAAGAAGTTGCXXXXXXXXXXGTTGAGGCGGAGAAGAAAAAGGGGATTGACATGTCCAAGT | lmxll |
| Marker2356 | TAAGAGCACTCCTCTCTGCAGAGACAAATAACCACTATAAXXXXXXXXXXATAGGATTTCGGCAAAGATGTTTGGGAGGAGGAAGACCTT | hkxhk |
| Marker2356 | GAGCACTCCTCTCTGCAGAGACAAATAACCACTATAAGTTXXXXXXXXXXATAGGATTTCGGCAAAGATGTTTGGGAGGAGGAAGACCTT | hkxhk |
| Marker2360 | TAAAAATGGAGAGAGATGACAACCTCAATCATAAGAATGGXXXXXXXXXXACAACAATTTATGTTGTTTGTTTGAATTTGTTTAGAGTAC | lmxll |
| Marker2360 | TAAAAATGGAGAGAGATGGCAACCTCAATCATAAGAATGGXXXXXXXXXXACAACAATTTATGTTGTTTGTTTGAATTTGTTTAGAGTAC | lmxll |
| Marker2364 | GCCACGAAGAATTCTTATGCAATATAAAGTAGAGACTATGXXXXXXXXXXATGTTGCATTGCATATTTCTGAATGACATCAAAAATAAAT | lmxll |
| Marker2364 | GCCACGAAGAATTCTTATGCAATATGAAGTAGAGACTATGXXXXXXXXXXCATGTTGCATTGCATATTTCTGAATGACATCAAAATAAAT | lmxll |
| Marker2365 | CCATGCCATAACAACAACACCAGTTTAGTTGCATGTACCAXXXXXXXXXXTTCAGCCCCCCATCTCTGCTTTTCCTGGAAAAAAAAATAT | lmxll |
| Marker2365 | CCATGCCATAACAACAACACCAGTTTAGTTGCATGTACCAXXXXXXXXXXTTCAGCCCCCCATCTCTGCCTTTCCTGGAAAAAAAAATAT | lmxll |
| Marker2366 | CTCTGAATAGTTATTGTCTAATAGGCGGATAAACTTTAGGXXXXXXXXXXAAGCAATTCAAGGACAATTCTCAATTTCTGTCAAATTTTT | lmxll |
| Marker2366 | CTCTGAATAGTTATTGTCTAATAGGCGGATAAACTTTAGGXXXXXXXXXXAAGCAATTCAAGGACAATTCTCAATTTCTGTCAATTTTTT | lmxll |
| Marker2372 | TCTAGTTGAATCAATTAGGAAATCAAATACATATATTTATXXXXXXXXXXCATTGAAAAGGACATGGTAGAAAGTTTGAATATGTAAAGT | lmxll |
| Marker2372 | TCTAGTTGAATCAATTAGGCAATCAAATACATATATTTATXXXXXXXXXXCATTGAAAAGGACATGGTAGAAAGTTTGAATATGTAAAGT | lmxll |
| Marker2375 | AAAAAAGGAAGAAAAATATAAGGGGAAGAGAAGCTGTCAGXXXXXXXXXXTTTTGTATTCATCCAGTTTTGTTGGCTTGGTTGCATGATT | lmxll |
| Marker2375 | AAAAGGAAGAAAAATATAAGGGGAAGAGAAGCTGTCAGCCXXXXXXXXXXTTTTGTATTCATCCAGTTTTGTTGGCTTGGTTGCATGATT | lmxll |
| Marker2380 | GTAAATTTTGCGTTGATTGTTATTTCAAACTGACCTTTCAXXXXXXXXXXTATGGTTGTGGAATTTGTTAGAGACCATTTCAAAGAAGGA | lmxll |
| Marker2380 | GTAAACTTTGCGTTGATTGTTATTTCAAACTGACCTTTCAXXXXXXXXXXTATGGTTGTGGAATTTGTTAGAGACCATTTCAAAGAAGGA | lmxll |
| Marker2383 | AACAAATATGTTTTGTTTTGTTATTTATGATTATATTGATXXXXXXXXXXGATTTGTTTGGCTTTTAGTTGCTTGTAGTCTCTGCATCAT | nnxnp |
| Marker2383 | AACAAATATTTTTTGTTTTGTTATTTATGATTATATTGATXXXXXXXXXXGATTTGTTTGGCTTTTAGTTGCTTGTAGTCTCTGCATCAT | nnxnp |
| Marker2392 | TATCCTATCCATGCACAAATTGAAAAGGGAGGAGAAGGAAXXXXXXXXXXTGCCAGAAACTTTAGATCATAAATTTATAAATATTTTTTT | lmxll |
| Marker2392 | TATCCTATCCATGCACAAATTGAAAAGGGAGGAGAAGGAAXXXXXXXXXXCTGCCAGAAACTTTAGATCATAAATTTATAAATATTTTTT | lmxll |
| Marker2399 | ACAAATTCCCAATATGAGCTCTTTCAATTTCAAATATTTTXXXXXXXXXXCTTATACCTACCCTTATACGTGTGTCAATATGCCTTTTAT | lmxll |
| Marker2399 | ACAAATTCCCAATATGAGCTCTTTCAATTTCAAATATTTTXXXXXXXXXXCTTATACCTACCCCTATACGTGTGTCAATATGCCTTTTAT | lmxll |
| Marker2401 | TAAATAACTTGCCAAATTTATCACAAGACTATGTGTGAGCXXXXXXXXXXATCTTGTTTTTACCAAGAGTCTCAAGGGTAGAGTTTTACC | lmxll |
| Marker2401 | TAAATAACTTGGCAAATTTATCACAAGACTATGTGTGAGCXXXXXXXXXXATCTTGTTTTTACCAAGAGTCTCAAGGGTAGAGTTTTACC | lmxll |
| Marker2402 | TCCCGTTATTTGGCAGAAGAATATGTATAGTTATTGCATTXXXXXXXXXXTAGACATGTGCCGATGTAATTTCGTCGGGAGAGGTTTTTT | hkxhk |
| Marker2402 | TCCCGTTATTTGGCAGAAGAATATGTATAGTTATTGCATTXXXXXXXXXXATAGACATGTGCCGATGTAATTTCGTCGGGAGAGGTTTTT | hkxhk |
| Marker2405 | TATTCAACAGGGTCATATTGCTTTAGTGACTGAGTGTTCAXXXXXXXXXXGATTTTCTTACCACTTTCATACATGCCGTTAGTCTTTCTA | nnxnp |
| Marker2405 | TATTCAACAGGGTCATATTGCTTTAGTGACTGAGTGTTCAXXXXXXXXXXGATTTTCTTACCATTTTCATACATGTCGTTAGTCTTTCTG | nnxnp |
| Marker2418 | TGTGCTTGATAATAATCATATGAAGTTTTTTTTTATTATGXXXXXXXXXXTTTTGCATCAGTACCCCCATAATAACTTCCTGAAAATGAT | lmxll |
| Marker2418 | TGTGCTTGATAATAATCGTATGAAGTTTTTTTTTATTATGXXXXXXXXXXTTTTGCATCAGTACCCCCATAATAACTTCCTGAAAATGAT | lmxll |
| Marker2420 | CATACTGTAAGCATAAAAAAACCAAAAGGTCAAAACTTTTXXXXXXXXXXCTGAGAGGTTTTCCACTTTGTCAACTCTCTCTCTCTCTTA | lmxll |
| Marker2420 | CATACTGTAAGCATAAAAAAACCAAAAGGTCAAAACTTTTXXXXXXXXXXCTGAGAGGTTTTCCACTTTGTCAACTCTCTCTCTCTCTCA | lmxll |
| Marker2422 | TGGGGACAAAATATTCATGAAAAAAGTGCATTTAGTGATGXXXXXXXXXXAATAGGGTGGGCTAGATTTACCTTATTGAACTGTTAGATT | nnxnp |
| Marker2422 | TGGGGACAAAATGTTCATGAAAAAAGTGTATTTAGTGATGXXXXXXXXXXAATAGGGTGGGCTAGATTTACCTTATTGAACTGTTAGATT | nnxnp |
| Marker2426 | AGAGTTGGCCCAAGCATACGAGGTTCTGAGTGACCCAGAGXXXXXXXXXXGCTCGCAACATAATCTGCTCCAAGTGCAGGGGGTTAGTAT | nnxnp |
| Marker2426 | AGAGTTGGCCCAAGCATACGAGGTTCTGAGTGACCCAGAGXXXXXXXXXXTCTCGCAACATAATCTGCTCCAAGTGCAGGGGGTTAGTAT | nnxnp |
| Marker2428 | AACAAAAAAGAAAGGCATAGAAAAGAAAAGGACATGGATCXXXXXXXXXXTGACTATTTATGCTTTCCCCTTTTCTCAGTTATCATAATT | lmxll |
| Marker2428 | AACAAAAAAGAAAGGGATAGAAAAGAAGAGGACATGGATCXXXXXXXXXXTGACTATTTATGCTTTCCCCTTTTCTCAGTTATCATAATT | lmxll |
| Marker2432 | AGTCTCATAATTTTAGCTAGAGAAGTGTTAGAGTCGAGTGXXXXXXXXXXTTAGCAACAGCTAATATGCATCAATTTTAGCTGCTACGAG | lmxll |
| Marker2432 | AGTCTCATAATTTTAGCTTGAGAAGTGTTAGAGTCGAGTGXXXXXXXXXXTTAGCAACAGCTAATATGCATCAATTTTAGCTGCTACGAG | lmxll |
| Marker2434 | GCTTGTAAATGTACTTGAAATGGTATATCTTTGCCTGTTAXXXXXXXXXXTGTATTTCACATTCGAAGTAGTGAGCCACGAAAACTTGTA | lmxll |
| Marker2434 | GCTTGTAAATGTACTTGAAATGGTATATCTTTGCCTGTTAXXXXXXXXXXTGTATTTCACATTCGAAGTAGTTAGCCACGAAAACTTGTA | lmxll |
| Marker2436 | AGAGGCCACATCATTGTTGAGGAGGAATGGCTCCTGATATXXXXXXXXXXCACCTGTGGAGGATTTCTGTATTTATTTCACATCAAGGAA | lmxll |
| Marker2436 | AAAGGCCACATCATTGTTGAGGAGGAATGGCTCCTGATATXXXXXXXXXXCACCTGTGGAGGATTTCTGTATTTATTTCACATCAAGGAA | lmxll |
| Marker2446 | GAAGACATCCGAAGGGCCTGGGCAAACTCCGACGAGCCTGXXXXXXXXXXAACCGGGGAGGTACCTAAGGTAGTGCTAATAACACTCAAA | lmxll |
| Marker2446 | GAAGACATCCGAAGGGCCTGGACAAACTCCGACGGGCCTGXXXXXXXXXXAACCGGGGAGGTACCTAAGGTAGTGCTAAAAACACTCAAA | lmxll |
| Marker2448 | CACATAAAAGCTCACCATCACTTTCTGTGACTTCTTCAGCXXXXXXXXXXGGATGTCAATAACATAAGAGCCTTATCCTCCAGACTATAA | efxeg |
| Marker2448 | CACATAAAAGCTCACCATCACTTTCTGTGACTTCTTCAGCXXXXXXXXXXGGATGTCAATAACATAAGAGTCTTATCCTCCAGACTATAA | efxeg |
| Marker2453 | ATCATAATGTGTATCATTCTAACAAGTTTGAATGGGTTGGXXXXXXXXXXCTTGGTTTCTGCATCCGCTGCTTTGGAAAAGATGAAGTGA | lmxll |
| Marker2453 | ATCATAATATGTATCGTTCTAACAAGTTTGAATGGGTTGGXXXXXXXXXXCTTGGTTTCTGCATCCGCTGCTTTGGAAAAGATGAAGTGA | lmxll |
| Marker2455 | ACCACGAGTGAAACAACTAACAAACCCTAACAAATAAAGCXXXXXXXXXXGAGTTCGATGATCGAAGGCCAAATCGCATAGTTGGATCTA | nnxnp |
| Marker2455 | ACCACGAGTGAAACAACTAACAAACCCTAACAAATAAAGCXXXXXXXXXXGAGTTCGATCATCGAAGGCCAAATCGCATAGTTGGATCTA | nnxnp |
| Marker2456 | GAGAAATCCTCACATTTGAAGCATATAGTTATATATACATXXXXXXXXXXTTCCAACCAAATGCAGCTCAAATTTATAAATACATAAATT | lmxll |
| Marker2456 | GAGAAATCCTCACATTTGAAGCATATAGTTATATATACATXXXXXXXXXXTTTCCAACCAAATGCAGCTCAAATTTATAAATACATAAAT | lmxll |
| Marker2464 | GCGTGAGAAGGCACAGAATAAGACACACAATGTCTCCATCXXXXXXXXXXATAGATTTATTTTGGATGAAGAACAGCAACAATTATCCTA | lmxll |
| Marker2464 | GCGTGAGAAGGCACAAAATAAGACACACAATGTCTCCATCXXXXXXXXXXATAGATTTATTTTGGATGAAGAACAGCAACAATTATCCTA | lmxll |
| Marker2471 | AATTTTGCGAGTAACAATCATTTATGCATGTGTCGGTGACXXXXXXXXXXCCCTTCTGCATATTTCTGTGCACCAAAACCTGACACCTCC | efxeg |
| Marker2471 | AATTTTGCGAGTAACAATCATTTATGCATGTGTCGGTGACXXXXXXXXXXCCCTTCTGCATATTTCTATGCACCAAAACCTGACACTTCC | efxeg |
| Marker2473 | ATTATTCTTCGTTCTTTCTTCATAAATTGTTCTTGGTTTTXXXXXXXXXXAATTGTTGTGATCCCAAGTTGAATGATTGCAATCGGTACG | nnxnp |
| Marker2473 | ATTATTCTTCGTTCTTTCTTCATAAATTGTTCTTGGTTTTXXXXXXXXXXAATTGTTGTGATCCCAAGCTGAATGATTGCAATCGGTACG | nnxnp |
| Marker2476 | TCTCACGAACGCGTAGGCACAAACGGTTCGTGAACCCGAGXXXXXXXXXXTTGAGCCACTTACACTTCGAATTGGACCTCGAGGTAAGAA | lmxll |
| Marker2476 | TCTCACGAACGCGTAGGCGCAAACGGTTCGTGAACCCGAGXXXXXXXXXXTTGAGCCACTTACACTTCGAATTGGACCTCGAGGTAAGAA | lmxll |
| Marker2480 | ATCAACACAGTAACAGTAAGAAACACCTAACTCAAAACTCXXXXXXXXXXTTGGAACAAGATCCTTGGAAGGCGAGCCCAGCACAGACAT | nnxnp |
| Marker2480 | ATCAACACAGTAACAGTAAGAAACACCTAACTCAAAACTCXXXXXXXXXXTTGGAACAAGATTCTTGGAAGGCGAGCCCAGCACAGACAT | nnxnp |
| Marker2481 | TTTCTATTTACTAAAAAACGAATCCAAAAGTCACTGTTCAXXXXXXXXXXAGCTCAAAGGATTTCTGGGTTTCGTAAAAAAAGGTTGGTT | nnxnp |
| Marker2481 | TTTCTATTTACTAAAAAAACGAATCCAAAAGTCACTGTTCXXXXXXXXXXAGCTCAAAGGATTTCTGGGTTTCGTAAAAAAAGGTTGGTT | nnxnp |
| Marker2486 | GGTATTTTCAATAGGATTGGAGTGCACTTTCAAAATAGTAXXXXXXXXXXGGATATCCTTTTCCTTGCTCTGGATGAACTGGAGAAGAAT | nnxnp |
| Marker2486 | GGGATTTTCAATAGGATTGGAGTGCACTTTCAAAATAGTAXXXXXXXXXXGGATATCCTTTTCCTTGCTCTGGATGAACTGGAGAAGAAT | nnxnp |
| Marker2490 | ACCCTAAACCAGTAAAACCCTTCCCCTCTCTCTCTCTCTCXXXXXXXXXXTCAGAAACCGGGCACAACCCTATGAACCCCAACGAGCACC | lmxll |
| Marker2490 | ACCCTAAACCAGTAAAACCCTTCCCCTCTCCCCCTCTCTCXXXXXXXXXXTCAGAAACTGGGCACAACCCTATGAACCCCAACGAGCACC | lmxll |
| Marker2491 | GATCAAGCTTTAGATCAAGTGGATCCGAAGAAGCATCAAAXXXXXXXXXXACTATCTTCGAGGTGATCGAGCCGGGCGGCAAAGCACCCT | lmxll |
| Marker2491 | GATCAAGCTTCAGATCAAGTGGATCCGAAGAAGCATCAAAXXXXXXXXXXACTATCTTCGAGGTGATCGAGCCGGGCGGCAAAGCACCCT | lmxll |
| Marker2493 | CAATATAAAAGCACATGGTGGTTCTACCATCTGTGCAGACXXXXXXXXXXGAAATCTTTGGATGCGCTATTTGTTTTCGTTCCCAGTAAA | lmxll |
| Marker2493 | CAATATAAAAGCACATGGTGGTTCTACCATCTGTGCAGACXXXXXXXXXXGAAATCTTTGGATGCGCTATTTGTTTTCATTCCCAGTAAA | lmxll |
| Marker2497 | AAAGTGAAGATTATCCTCTAAATGCAATCAAACAAGTGTCXXXXXXXXXXTGGTTTATATTTATTCACTCATTCAAAAAGTGAAAGTAAC | nnxnp |
| Marker2497 | AAAGTAAAGATTATCCTCTAAATGCAATCAAACAAGTGTCXXXXXXXXXXTGGTTTATATTTATTCACTCATTCAAAAAGTGAAAGTAAC | nnxnp |
| Marker2499 | ACTCACCATGCAATGAATTTCCTTCTAATTTTTTTTTGTGXXXXXXXXXXTACTAACTCATAATGAATCAAAATTTATGCGAATTTTATG | nnxnp |
| Marker2499 | ACTCACCATGCAATGAATTTCCTTCTAATTTTTTTTGTGAXXXXXXXXXXTACTAACTCATAATGAATCAAAATTTATGCGAATTTTATG | nnxnp |
| Marker2503 | ACAAAAGGGCCTGTTTCTGTAAATGACCATGAGGCTGCTAXXXXXXXXXXAGGGTAAAAAGGGTTTCTTCTGAATTTGCTGGCCATGACA | nnxnp |
| Marker2503 | TCAAAAGGGCCTGTTTCTGTAAATGACCATGAGGCTGCTAXXXXXXXXXXAGGGTAAAAAGGGTTTCTTCTGAATTTGCTGGCCATGACA | nnxnp |
| Marker2507 | ACCGGTATCAAAAACTTGATACCGCTACCGTACCATTCTTXXXXXXXXXXCTCAATAGCTTTCATCTCTTCCTTTATAGCTTGAACTCAC | lmxll |
| Marker2507 | ACCGGTATCAAAAACTTGATACCGCTACCGTACCATTCTTXXXXXXXXXXCTCAAGAGCTTTCATCTCTTCCTTTATAGCTTGAACCCAC | lmxll |
| Marker2508 | AGCAAATTATCCTTTTATTCAAACACCACTCTTTATCGATXXXXXXXXXXGGGCTCTATATTATAGAGTTCCAAGGAGGTTGAGCCTAAT | lmxll |
| Marker2508 | AGCAAATTATCCTTTTATTCAAACACCACTCTTTATCGATXXXXXXXXXXGGGCTCTATATTATAGAGCTCCAAGGAGGTTGAGCCTAAT | lmxll |
| Marker2512 | TTACAGTTCTGCCTTTCAGCGCTTTTGAGAAACGAAAGTCXXXXXXXXXXTTGTAGTTGTTTTTCTTTTTTGTTCTTGAATTTACTTGCT | nnxnp |
| Marker2512 | TTACAGTTCTGCCTTTCAGCGCTTTTGAGAAACGAAAGTCXXXXXXXXXXTTGTAGTTGTTTTTCTTTTTTGTTCTTGTATTTACTTGCT | nnxnp |
| Marker2514 | AAAATAAAAAAGAAACCCTACGACACGTTCTGAGAAGAAGXXXXXXXXXXTTTCTTCGTCTCTCCTGTGTGCTCTTCTTTCTTTTTCTTG | lmxll |
| Marker2514 | AATATAAAAAAGAAACCCTATGACACGTTCTGAGAAGAAGXXXXXXXXXXTTTCTCCGTCTCTCCTGTGTGCTCTTCTTTCTTTTTCTTG | lmxll |
| Marker2518 | GTAATTGAGGCTATGATTGAGGTTGACCCACAATATGTGAXXXXXXXXXXTGTTTTACCCCCAAATCTTGCAATATATAACGCAACCAAG | lmxll |
| Marker2518 | GTAATTGAGGCTATGATTGAGGTTGACCCACGATATGTGAXXXXXXXXXXTGTTTTACCCCCAAATCTTGCAATATATAACGCAACCAAG | lmxll |
| Marker2524 | GAATAATCTTGTACTTTTTTTGTGTGTAGATGGAAAATTTXXXXXXXXXXGGCTATTGTTGAAGTGAAAGGCGTGCACCAGTTTGATTGG | lmxll |
| Marker2524 | GAATAATCTTGTACTTTTTTTGTGTGTAGATGGAAAAATTXXXXXXXXXXGGCTATTGTTGAAGTGAAAGGCGTGCACCAGTTTGATTGG | lmxll |
| Marker2540 | CTTTGAAAATTTGGTATATGAAAGCATAGTTGGAAAGCGAXXXXXXXXXXCCAAGTGATGGATACATTGTGGGAATATTTGGATAGAATT | hkxhk |
| Marker2540 | CTTTGAAAATTTGGTATATGAAAGCATAGTTGGAAAGCGAXXXXXXXXXXCTAAGTGATGGATACATTGTGGGAATATTTGGATAGAATT | hkxhk |
| Marker2544 | ATCCTTTTCTTCTATCATCCTCATCATTTCCCTACCTTTGXXXXXXXXXXGATGAGGTCATGATATTCATGAGACTTGTTCCAATGGATG | lmxll |
| Marker2544 | AGCCTTTTCTTCTATCATCCTCATCATTTCCCTACCTTTGXXXXXXXXXXGATGAGGTCATGATATTCATGAGACTTGTTCCAATGGATG | lmxll |
| Marker2547 | CAATTGAAGGAAGAAGAAGAAGCAGCAGATTATGAGCCCTXXXXXXXXXXTGGCCACGGATTCTTCATCTGATGGTACCCTTTTGTTCTT | lmxll |
| Marker2547 | CAATTGGAGGAAGAAGAAGAAGCAGCAGATTATGAGCCCTXXXXXXXXXXTGGCCACGGATTCTTCATCTGATGGTACCCTTTTGTTCTT | lmxll |
| Marker2550 | AATGAAAGAGAGGACAAGGAGACAGCGGTGCGTCGTGTCAXXXXXXXXXXCTAAGTCATTATAATAATCTAACGACACTCATTTGTATAA | hkxhk |
| Marker2550 | AATGAAAGAGAGGACAAGGAGACAGCGGTGCGTCGTGTCAXXXXXXXXXXCTAAGTCATTATAATAATCTAACGACACTCATCTGTATAA | hkxhk |
| Marker2552 | CCAGTCACTTCAGTACCCTTTTGCAGGCCTAATTTCCTGAXXXXXXXXXXTTTTTTGCATTCTGCCATACGATGTCCAGTTTCCCCGCAC | lmxll |
| Marker2552 | CCCGTCACTTCAGTACCCTTTTGCAGGCCTAATTTCCTGAXXXXXXXXXXTTTTTTGCATTCTGCCATACGATGTCCAGTTTCCCCGCAC | lmxll |
| Marker2567 | AAGGTGTTTGACGAAAGATCGACATTCTGTAGGAATTTTAXXXXXXXXXXCTATACTGAGGGCCTTAGCCTTTAGGTAAGGTCTTATCTC | lmxll |
| Marker2567 | AAGGTGTTTGATGGAAGATCGACATTCTGTAGGAATTTTAXXXXXXXXXXCTATACTGAGGGCCTTAGCCTTTAGGTAAGGTCTTATCTC | lmxll |
| Marker2568 | GAAATAATAGAGGTAAATTCTTGTCTCCTCCAGGTAGCAGXXXXXXXXXXTGCTCCCATTTATGCTCAAGCAGTAGAGAGAAATCTAAGT | lmxll |
| Marker2568 | GAAACAATAGAGGTAAATTCTTGTCTCCTCCAGGTAGCAGXXXXXXXXXXTGCTCCCATTTATGCTCAAGCAGTAGAGAGAAATCTAAGT | lmxll |
| Marker2570 | GTTTTTGGGCATTGTTGTTGCTATTTTTTCTTGGGGCCAAXXXXXXXXXXGCTCAAGATCTCAGAAACTAAAGTAAATTACATGTTCAAG | lmxll |
| Marker2570 | GTTTTTGGGCATTGTTGTTGCTCTTTTTTCTTGGGGCCAAXXXXXXXXXXGCTCAAGATCTCAGAAACTAAAGTAAATTACATGTTCAAG | lmxll |
| Marker2574 | AAACATAGCAGCCCTAATGTCACAAATTCCACCATTACATXXXXXXXXXXCAGAGTTGTGGATGAAATATGATAAAATCTAACGACAGAG | lmxll |
| Marker2574 | AAACATAGCAGCCCTAATGTCACAAATTCCACCATTACATXXXXXXXXXXCAGAATTGTGGATGAAATATGATAAAATCTAACGACAGAG | lmxll |
| Marker2577 | ATGCATCTGGTATTTGATTTGCAATACTTTGGAGATGAACXXXXXXXXXXGGTGGTGCAATGAGCACATATACCGCACAACCAAAAACTC | lmxll |
| Marker2577 | ATGCATATGGTATTTGATTTGCAATACTTTGGAGGTGAACXXXXXXXXXXGGTGGTGCAATGAGCACATATACCGCACAACCAAAAACTC | lmxll |
| Marker2582 | GGGTGAAAATTTTCCAATGAAATTATTTAGTAACCTGAATXXXXXXXXXXATTCTTTTCCTGTTCTTGGGAGAGAATAACAGATTCTCCA | nnxnp |
| Marker2582 | GGTGAAAATTTTCCAATGAAATTATTTAGTAACCTGAATCXXXXXXXXXXATTCTTTTCCTGTTCTTGGGAGAGAATAACAGATTCTCCA | nnxnp |
| Marker2586 | ACTTCATAGACATGGCTCAACAAGGAACAAAACAAGCACTXXXXXXXXXXAGTAAGTAAATAGGGCATGTATATGTATGAAATTATGAGC | lmxll |
| Marker2586 | ACTTCATAGACATGGCTCAACAAGGAACAAAACAAGCACTXXXXXXXXXXAGTAAGTAAATAGGGCATGTATATGTACGAAATTATGAGC | lmxll |
| Marker2588 | ATAATTATAGACTTGTAGTCTTTATAGACATTAGAGTAACXXXXXXXXXXACCAATTTGTTCTTCTCTTATGGGGTTAGTGTACTGTATT | lmxll |
| Marker2588 | AGAATTATAGACTTGTAGTCTTTATAGACATTAGAGTAACXXXXXXXXXXACCAATTTGTTCTTCTCTTATGGGGTTAGTGTACTGTATT | lmxll |
| Marker2592 | AAAGAGGACAGGGTTGAAGGCAGATGTGTTTATCGGGACTXXXXXXXXXXTTATAGCTGTGTTATAGACTTGCTCTGTAAGGCAGGTCGG | lmxll |
| Marker2592 | AAAGAGGAAAGGGTTGAAGGCAGATGTGTTTATCGGGACTXXXXXXXXXXTTATAGCTGTGTTATAGACTTGCTCTGTAAGGCAGGTCGG | lmxll |
| Marker2593 | ACAACGTTTCATCTAAGAACAAGGGCATGCCTGTATTTCCXXXXXXXXXXTAATTCTCTCTGCATTTGCAACGCGGCACCTTGGATATGA | nnxnp |
| Marker2593 | ACAACTTTTCATCTAAGAAGAAGGGCATGCCTGTATTTCCXXXXXXXXXXTAATTCTCTCTGCATTTGCAATGCGGCACCTTGGATATGA | nnxnp |
| Marker2594 | TTTACTTCGATTATTTATTTTTTGTTGTAGGGGGATATTGXXXXXXXXXXAATCTCTGTCTATGTTATGCTTTCTTCCGGAACCTGGACC | lmxll |
| Marker2594 | TTTACTTCGATTATTTATTTTTTTGTTGTAGGGGGATATTXXXXXXXXXXAATCTCTGTCTATGTTATGCTTTCTTCCGGAACCTGGACC | lmxll |
| Marker2600 | CACAACACAAAAGCACAATACAAATTGGAAACTAAAAATGXXXXXXXXXXTTCAAGTGACCTAATTCTAATGCATGAAAGTAAATGTACT | lmxll |
| Marker2600 | CACAACATAAAAGCACAATACAAATTGGAAACTAAAAATGXXXXXXXXXXTTCAAGTGACCTAATTCTAATGCATCAAAGTAAATGTACT | lmxll |
| Marker2610 | GGATTCAGTGAAGGCACTTGCCTCCCCTCAGTCCTGTTCTXXXXXXXXXXCCAAAGGGGCATGGGAGGAAGGAGCAAGGATATCAATTAT | hkxhk |
| Marker2610 | GGATTCAGTGAAGGCACTTGCCTCCCCTCAGTCCTGTTCTXXXXXXXXXXCCAAAGGGGCATGGGAGGAAGGAGCAAGGACATCAATTAT | hkxhk |
| Marker2612 | CATCCATTGGAACAAGTCTCATGAAGATCATGACCTCATCXXXXXXXXXXCTCTACAAAAAATCACTTCAATCTAATATCATTTGACCAC | nnxnp |
| Marker2612 | CATCCATTGGAACAAGTCTCATGAAGATCATTACCTCATCXXXXXXXXXXCTCTACAAAAAATCACTTCAATCTAATATCATTTGACCAC | nnxnp |
| Marker2619 | TTTTGTGGAATAAATATCTGCATTCAATTTACTGTTTGTGXXXXXXXXXXCATATTCCAACAAATTATCATTTTTTGGTAGCACACAGGC | lmxll |
| Marker2619 | TTTTGTGGAATAAATATCTGCATTCAATTTACTGTTTGTGXXXXXXXXXXCATATTCCAACAAATTATCAGTTTTTGGTGGCACACAGGC | lmxll |
| Marker2629 | GCCAGAAAAGAATGGACTGAGTAAGAGCATACCCAACTGAXXXXXXXXXXTTTTTTTTTTCTTTTCGAGTCAACTTTTGCTTTTTCTCTT | nnxnp |
| Marker2629 | GCCAGAAAAGAATGGACTGAGTAAGAGCATACCCAACTGAXXXXXXXXXXTTTTTTTTTTCTTTTTGAGTCAACTTTTGCTTTTTCTCTT | nnxnp |
| Marker2633 | ATCTTTATATATTGAAAAGCATTGAGTAATGGCATTCTTGXXXXXXXXXXAAGCTTTATAGTTGTCTTTGACAAAAGCAAACACCTTACT | lmxll |
| Marker2633 | ATCTTTCTATATTGAAAAGCATTGAGTAATGGCATTCTTGXXXXXXXXXXAAGCTTTATAGTTGTCTTTGACAAAAGCAAACACCTTACT | lmxll |
| Marker2639 | AATTATTTGTCTAATGCCTGCTAAAGATTGTGGATATTAGXXXXXXXXXXGCATATTTTTGTCTGAATTATTGGATTGAACATGACATCA | nnxnp |
| Marker2639 | AATTATTTGTCTAATGCCTGCTAAAGATTGTGGATATTAGXXXXXXXXXXGCATATTTTTGTCTGAATTATGGGATTGAACATGACATCA | nnxnp |
| Marker2642 | AGGACTAAAATATCGACAAAAAATAAGGAATAAAATCTTGXXXXXXXXXXTGATATGAACAGCGGGCATATAAATGCTTGAAGTTTTGGG | lmxll |
| Marker2642 | AGGACTAAAATATCGACAAAAAATAAGGAATAAAATCTTGXXXXXXXXXXTGATATGAACAGCGGGCATATAAATGCTTCAAGTTTTGGG | lmxll |
| Marker2645 | GGATTGTGTTTCAGTGTGGCTGAAAATTCACAGCAATTGAXXXXXXXXXXAACCAACTCAAAGGCCATCAATTGGCCTATTGTTATTTGT | nnxnp |
| Marker2645 | GGATTGTGTTTGAGTGTGGTTGAAAATTCACAGCAATTGAXXXXXXXXXXAACCAACTCAAAGGCCATCAATTGGCCTATTGTTATTTGT | nnxnp |
| Marker2652 | ATGTGTGACATGAAATTTGATATTGATCATACCAATTTTTXXXXXXXXXXGAAAACGATAGCCCCCACAAAGGTAGCCAAAAGATGCAGA | lmxll |
| Marker2652 | ATGTGTGACATGAAATTTGATATTGATCATACCAATTTTTXXXXXXXXXXGAAAACGATAGCCCCCACAAAGGTCGCCAAAAGATGCAGA | lmxll |
| Marker2655 | TTTGATGATGCCAACATGTTTAGAATGTATAATATGATGTXXXXXXXXXXTCACGAATCATCTTTTCTACAAAAACTCACACAAGACACA | lmxll |
| Marker2655 | TTTGATGATGCCAACATGTTTAGAATGTATAATATGATGTXXXXXXXXXXTCACGAACAATCTTTTCTACAAAAACTCACACAAGACACA | lmxll |
| Marker2659 | GTGACATGTGAACCCCAAATATTTGGATGTCGTCCGCGTGXXXXXXXXXXAAAAGAAAGAAAGGTTTCTTTCCCCAAATGGACAAACCTA | lmxll |
| Marker2659 | GTGACATGTGAACCCCAAATATTTGAATGTCGTCCGCGTGXXXXXXXXXXAAAAGAAAGAAAGGTTTCTTTCCCCAAATGGACAAACCTA | lmxll |
| Marker2664 | CTCTCCTTTCATCATTCTGAAACCTCCACCGCTCTCTCTCXXXXXXXXXXGCCTTGGCGGTGCACGACAAGGACACGCCAGACCGGTGGA | lmxll |
| Marker2664 | CTCTCCTTTCATTATTCTGAAACCTCCACCGCTCTCTCTCXXXXXXXXXXGCCTTGGCGGTGCACGACAAGGACACGCCAGACCGGTGGA | lmxll |
| Marker2665 | TAAGAAAGGCAACAACCATGATAGCATCACCCCAAAGCTTXXXXXXXXXXAGAAGAAGTAGGAGAAGAAAACGGACCCCAAACATCAGTG | lmxll |
| Marker2665 | TAAGAAAGGCAACAGCCATGATAGCATCACCCCAAAGCTTXXXXXXXXXXAGAAGAAGTAGGAGAAGAAAACGGACCCCAAACATCAGTG | lmxll |
| Marker2669 | CCTGAACATGCAATTTACTTTTCTGAGATCTTGAGCAGAGXXXXXXXXXXTTGGCCCCAAGAAAAAAGAGTAACAACAATGCCCAAAAAC | lmxll |
| Marker2669 | CCTGAACATGTAATTTACTTTTCTGAGATCTTGAGCAGAGXXXXXXXXXXTTGGCCCCAAGAAAAAAGAGTAACAACAATGCCCAAAAAC | lmxll |
| Marker2683 | CCTACGGGCGCATGTTAGGGTGTCGTCATTTTATTGTCTCXXXXXXXXXXAAATTATTGGGACATGGTCATGCCCAACTCGAACTCATTA | lmxll |
| Marker2683 | CCTACGGGCGCATGTTAGCGTGTCGTCGTTTTATTGTCTCXXXXXXXXXXAAATTATTGGGACATGGTCATGCCCAACTCGAACTCATTA | lmxll |
| Marker2684 | TTATGGGTATGATAGTAAACATGTTTGGACCGTAGGTAAGXXXXXXXXXXGGTGAAAATATCAATAGCCATAGGCATATATCCACACTGA | nnxnp |
| Marker2684 | TTATGGGTATGATAGTAAACATGTTTGGACCTTAGGTAAGXXXXXXXXXXGGTGAAAATATCAATAGCCATAGGCATATATCCACACTGA | nnxnp |
| Marker2692 | TTTGACTCCACATACATAAGATCAAAGGGTTGAATTTCATXXXXXXXXXXTTTGTCTAATTTTTTGCAATAATGATCTATAAATGTAGAC | lmxll |
| Marker2692 | TTTGACTCCACATACATAAGATCAAAGGGTTGAATTTCATXXXXXXXXXXTTTGTCTAATTTTTTGCATGAATGATCTATAAATGTAGAC | lmxll |
| Marker2695 | GTTCACCAGAGATCAAGGATTCGGTGCTGGAATCTTGATTXXXXXXXXXXGTGGTGGTGGGAAAGATGGTCATTACAAATGTACAAAATA | lmxll |
| Marker2695 | GTTCACCAGAGATCAAGGATTCGGTGCTGGAATCTTGATTXXXXXXXXXXGTGGTGGTGGGAAAGATGCTCATTACAAATGTACAAAATA | lmxll |
| Marker2696 | TGGTTTGCACTTTGATTTTGATTAGGTGTGGACAGCGTTGXXXXXXXXXXTGATGAGAACCCTCATGCTTGTTCTATAATGTGATGATAT | nnxnp |
| Marker2696 | TGGTTTGCACTTTGATTTTGATTAGGTGTGGACAGCGTTGXXXXXXXXXXTGATGAGAACCCTCACGCTTGTTCTATAATGTGATGATAT | nnxnp |
| Marker2698 | TCAATAACAAATTCATATTCTATACACAAATACAACGTAAXXXXXXXXXXCCATTCCACTCCAGATAATCAGAAATTACCATTTCAGACT | lmxll |
| Marker2698 | TCAATAACAAATTCATATTCTATACACAAAAACAACGTAAXXXXXXXXXXCATTCCACTCCAGATAATCAGAAATTACCATTTTCAGACT | lmxll |
| Marker2704 | GCTCCCAAAATCCCTGATTTTATGCAAAGGAGCAAAGTAAXXXXXXXXXXCCCTCATTCCCAACCAAACCTCTCTTCAAAATCCCTTTTC | nnxnp |
| Marker2704 | GCTCCCAAAATCCCTGATTTTATGCAAAGGAGCAGAGTAAXXXXXXXXXXCCCTCATTCCTAACCAAACCTCTCTTCAAAATCCCTTTTC | nnxnp |
| Marker2710 | ACGACATGTCGTTTCTCTGATCCTCATTTTGACTCTGAAGXXXXXXXXXXGTTCTTATACGATATTTCTCTAAAACCGAGCAATATGTAA | lmxll |
| Marker2710 | ACGACATGTCGTTTGTATGATCCTCATTTTGACTCTGAAGXXXXXXXXXXGTTCTTATACGATATTTCTCTAAAACCGAGCAATATGTAA | lmxll |
| Marker2712 | ACCTTGGAATTGGAGGAAAATACCACACCACCTTCGCTGGXXXXXXXXXXGGTTGATCTCCATTCCCAATCACATTGGGAACAGACGCAA | nnxnp |
| Marker2712 | ACCTTGGAATTGGAGGAAAATACCACACCACCTTCGCTGGXXXXXXXXXXGGTTGATCTCCATTCCCAATCACATTGGGAACGGACGCAA | nnxnp |
| Marker2714 | GGAGAGATACGAAGTAAAGAGCAGAATTGGAGTATGTGCAXXXXXXXXXXGTCTGAACCACTCAAGGCATTTGTGCATCAGTTGTCTACT | lmxll |
| Marker2714 | GGAGAGATACAAAGTGAAGAGCAGAATTGGAGTATGTGCAXXXXXXXXXXGTCTGAACCACTCAAGTCATTTGTGCATCAGTTGTCTACT | lmxll |
| Marker2716 | ATGAGTTGGAAGTTTGGAACGGTTTCAATCAGTAAAATTAXXXXXXXXXXGGTAAGAGGAAGAATATATTAGATCACTCTTATAGAAGCT | hkxhk |
| Marker2716 | ATGAGTTGGAAGTTTGGAACGGTTTCAATCAGTAAAATTAXXXXXXXXXXAGTAAGAGGAAGAATATATTAGATCACTCTTATAGAAGCT | hkxhk |
| Marker2721 | TTAGGAAATAAGTGGAATCCTAATTACATAATATCTATACXXXXXXXXXXCGTATGATATGTATGTATAATTTATGAATGGCTTTACACT | lmxll |
| Marker2721 | TTAGGAAATAAGTGGAATCCTAATTACATAATATCTATACXXXXXXXXXXGTATGATATGTATGTATAATTTATGAATGGCTTTACACTT | lmxll |
| Marker2722 | ATATTGGACTCATTGATGTTTATTTACTTTGAGAATAGGAXXXXXXXXXXTGGAGAAGAAGTCAAAGAAGGAATTTGTCTTACTTTTCTG | lmxll |
| Marker2722 | ATATTGGACTCATTGATGTTTATTTACTTTGAGAATAGGAXXXXXXXXXXTGGAGAAGAAGTCAAAGAAGGAATTTGTCTTACTTTTCCG | lmxll |
| Marker2728 | AAGATGCATTTGTTAGTAGTCATTTGTGTGTAGACAACGCXXXXXXXXXXCCAAGCATAAACGGTTGAGTAGACCAAATCAACTGAGGGG | nnxnp |
| Marker2728 | AAGATGCATTTGTTAGTAGTCATTTGTGTGTAGACAACGCXXXXXXXXXXCCAAGCATAAACGGTTGAGTAGACCAAATAAACTGAGGGG | nnxnp |
| Marker2735 | AGAGAATATTCGGGCGCCTATGCGCTAAGCGAGTTATTTTXXXXXXXXXXAGCTTCTATGTCTACCCCACCATCACTTTTGAAGGAAGTA | hkxhk |
| Marker2735 | AGAGAATATTCGGGCGCCTATGCGCTAAGCGAGTTCTTTTXXXXXXXXXXAGCTTCTATGTCTACCCCACCATCACTTTTGAAGGAAGTA | hkxhk |
| Marker2736 | CTGGTGCACTTAGATTTACCATTATAAGTAGCAAACAGGAXXXXXXXXXXACACAGATGCACAAACAGACAATTGCTCAAGAATCTGTAA | nnxnp |
| Marker2736 | CTGGTGCACTTAGATTTACCATTATAAGTACCAAACAGGAXXXXXXXXXXACACAGATGCACAAACAGACAATTGCTCAAGAATCTGTAC | nnxnp |
| Marker2741 | ATTATAATCAAGAAGACTGAGCAGTAAATGCCAATCGCCAXXXXXXXXXXGACCAGAAAGAGAATCTTACATCATAATCGCGTCCTGAAA | nnxnp |
| Marker2741 | ATTATAATCAAGAAGACTGAGCAGTAAATGCCAATCGCCAXXXXXXXXXXGACCAGAAGGAGAATCTTACATCATAATCGCGTCCTGAAA | nnxnp |
| Marker2756 | TTTCACATTGCTTCATCACTTCACTTGTCGTCGACAAGTGXXXXXXXXXXGAGAGAGAGAGAGAGAGAGAGAGAGAGAGAGATAAGGTAG | efxeg |
| Marker2756 | TTTCACATTGCTTCATCACTTTACTTGTCGTCGACAAGTGXXXXXXXXXXGAGAGAGAGAGAGAGAGAGAGAGAGAGAGAGATAAGGTAG | efxeg |
| Marker2759 | TTTTTTATTTTCCCTGCCGTTGTGATTTTAGCATCTGGTAXXXXXXXXXXGCCATTGTCCGAATCCCCCTCCTTCTCTGTCTCCACTTCC | lmxll |
| Marker2759 | TTTTTTATTTTCCCTGCCGTTGTGATTTTAGCATCTGGAAXXXXXXXXXXGCCATTGTCCGAATCCCCCTCCTTCTCTGTCTCCACTTCC | lmxll |
| Marker2762 | TTCACGTCTGAATTGGGGCACTTCAAAATTCACTACATTGXXXXXXXXXXAGATTCGTGCAGCCTTCCATATGAATCTTTGTCAAGGAGT | efxeg |
| Marker2762 | TTCACGTCTGAATTGGGGCACTTCAAAATTCACTACATTGXXXXXXXXXXAGATTCGTGCAGCCTTCCATATGAATCCTTGTCAAGGAGT | efxeg |
| Marker2767 | AACAGATATGCAAACAGAATAACAAAACAGAATAAATAGAXXXXXXXXXXTATGTAGAGGCATATTCTATAAAGCAACTCCACACACGAG | nnxnp |
| Marker2767 | AACAGATAAGCAAACAGAATAACAAAACAGAATAAATAGAXXXXXXXXXXGACGTAGAGGCATATTCTATAAAGCAACTCCACACACGAG | nnxnp |
| Marker2769 | TCAGGTGTGGTATAACATAGAATATGAAGGGCTCCCTGACXXXXXXXXXXACAGGTAAGAACCATCTGGTTTCAGCTGGTTCTTCAGGCT | lmxll |
| Marker2769 | TCAGGTGTGGTATAACATAGAATATGAAGGGCTCCCTGACXXXXXXXXXXACAGGTAATAACCATCTGGTTTCAGCTGGTTCTTCAGGCT | lmxll |
| Marker2774 | AATGGAGACTGGAGATTTCTTTATTCAATCTTGTACAAGAXXXXXXXXXXAGGCTTTTATGAGATTTGACAGAAGTTGTCACTATTAGAC | lmxll |
| Marker2774 | AATGGAGACTGGAGATTTCTTTATTCAATCTTGTACAAGAXXXXXXXXXXAGGCTTTGATGAGATTTGACAGAAGTTGTCACTATTAGAC | lmxll |
| Marker2778 | TGTCTGGATTTTGTGGATGACTGCTATTTGACAACAACTTXXXXXXXXXXTATCCAGGTATAGCAACTCAATCTTTCTCAATTCTATCCC | lmxll |
| Marker2778 | TGTCATGGATTTTGTGGATGACTGTTATTTGACAACAACTXXXXXXXXXXTATCCAGGTATAGCAACTCAATCTTTCTCAATTCTATCCC | lmxll |
| Marker2789 | AGGCAAACCGGAGATAAATAAGACATTTGTTTACAAACCAXXXXXXXXXXCAAATCGAAAACAAATTGAAGGAACTCCAACACTTCCGTC | lmxll |
| Marker2789 | AGGCAAACCGGAGATAAATAACACATTTGTTTACAAACCAXXXXXXXXXXCAAATCGAAAACAAATTGAAGGAACTCCAACACTTCCGTC | lmxll |
| Marker2792 | ACCCAGATAAGATAAAGTAATGATAGAGTACTCACCAATTXXXXXXXXXXGAGTTCATCTATGCTTATAAGGGTAGGGCAGAATAAGTAT | nnxnp |
| Marker2792 | ACCCAGATAAGATAAAGTAATGATAGAGTACTCACCAATTXXXXXXXXXXAAGTTCAGCTATGCTTATAAGGGTAGGGCAGAATAAGTAT | nnxnp |
| Marker2794 | TGAAAGCCGTGGGATCCAAACTGTACGGTAGCAAGAACTAXXXXXXXXXXGCCTGACTGACATGTGGTAGCTTTTTTTTCTTCTTCTCTC | lmxll |
| Marker2794 | TGAAAGCCGTGGGATCCAAACTGTACGGTAGCGAGAACTAXXXXXXXXXXGCCTGACTGACATGTGGTAGCTTTTTTTTCTTCTTCTCTC | lmxll |
| Marker2796 | TGTAAGAGCTGCTATCTTCAAGTTCTGGTCCAACCTCAGAXXXXXXXXXXGAAAGAATCAAAGAAGAGTTACTATGTATTGACTTCAAAG | lmxll |
| Marker2796 | TGTAAGAGCTGCTATCTTCAAGTTCTGGTCCAACCTCAGAXXXXXXXXXXAAGAATCAAAGAAGAGTTACTATATGTATTGACTTCAAAG | lmxll |
| Marker2800 | CCTCATATTATTACATTACATGGTTCAGGCAGATGGAGATXXXXXXXXXXGGTCAGGTGCAAATAGCATCTTGTGGAAGCTTGACAAGCA | lmxll |
| Marker2800 | CCTCATATTATTACATTACATGGTTCAGGCAGATGGAGATXXXXXXXXXXGGTCAGTAGCAAATAGCATCTTGTGGAAGCTTGACAAGCA | lmxll |
| Marker2804 | TGTGGACCCCCACTTTGACCTTTTCTCATGTGGGACCAAGXXXXXXXXXXACATTCGTAAGTGTCCACCGAGGGTTGGAAGGTTGGAATT | hkxhk |
| Marker2804 | TGTGGACCCCCACTTTGACCTTTGCTCATGTGGGACCAAGXXXXXXXXXXACATTCGTAAGTGTCCACCGAGGGTTGGAAGGTTGGAATT | hkxhk |
| Marker2806 | CCCCCCCAAAACAATATATTGCGCCTCTATGTTCTCGTGGXXXXXXXXXXATGGCTTTTCGGTGCTTCAACCAAGAAATCACATTTTTTA | lmxll |
| Marker2806 | CCCCCCAAAACAATATATTGCGCCTCTATGTTCTCGTGGGXXXXXXXXXXATGGCTTTTCGGTGCTTCAACCAAGAAATCACATTTTTTA | lmxll |
| Marker2808 | AGACCTCTTCTTTCAAATATTATCTGCTACTTTGCGCAAAXXXXXXXXXXAGCCCAGTGGCATAACCGTAAATATTTCAAACTCCAGTTT | lmxll |
| Marker2808 | ACACCTCTTCTTTCAAATATTATCTGCTACTTTGCGCAAAXXXXXXXXXXAGCCCAGTGGCATAACCGTAAATATTTCAAACTCCAGTTT | lmxll |
| Marker2810 | AGCTCTCCCCATGAGGTTCTTTACACATGCGGAGCATCGAXXXXXXXXXXCATAAGCTTCCTAAGTGGCGTTTGGCAATGTCTGATGAGT | efxeg |
| Marker2810 | AGCTATCCCCATGAGGTTCTTTACACATGCGGAGCATCGAXXXXXXXXXXCATAAGCTTCCTAAGTGGCGTTTGGCAATGTCTGATGAGT | efxeg |
| Marker2815 | CAAAATATAAATATACATGAACCAAAAAAAAAGAGTAAGGXXXXXXXXXXTTGTTTTTGTTTTTGTTTTTAGTCGAAGTAGATAGTAATA | efxeg |
| Marker2815 | CAAAATATAAATATACATGAACCAAAAAAAAAGAGTAAAGXXXXXXXXXXTTGTTTTTGTTTTTGTTTTTAGTCGAAGTAGATAGTAATA | efxeg |
| Marker2824 | TTGATACTGGTTCAGATCAGACCAGCAACAAATTATGCACXXXXXXXXXXAAAAACACAAGTCTTTTTGTTTTCACAAATTCTTTTTCGT | efxeg |
| Marker2824 | TTGATACTGGTTCAGATCAGACCAGCAACAAATTATGCACXXXXXXXXXXAAAAACTCAAGTCTTTTTGTTTTCACAAATTCTTTTTCGT | efxeg |
| Marker2832 | GGAATAATCACTCATGCAGATTCTATATAAATATGAGAGGXXXXXXXXXXTATCTTAGAATCACGGGAATAGTTCCCAAGTGATCCACAA | nnxnp |
| Marker2832 | GGAATAATCACTCATGCAGATTCTCTATAAATATGAGAGGXXXXXXXXXXTATCTTAGAATCACGGGAATAGTTCCCAAGTGATCCACAA | nnxnp |
| Marker2836 | AAACACGTACATACATATATAATAATCTCTCAAAATCCATXXXXXXXXXXCATCGTCGTTTTTTTTTCGATTTCAATTTTCGTTTCGGAA | lmxll |
| Marker2836 | AAACACGTACATACATATATAATAATCTCTCAAAATCCATXXXXXXXXXXCATCGTCGTATTTTTTTCGATTTCAATTTTCGTTTCGGAA | lmxll |
| Marker2855 | AAATTTCATCCAATCAAACTATCGCTTATATTAGAAGAAAXXXXXXXXXXCAGCAGAAATCTTGGTGTTCCAGATGTGCGATTTCGTAGA | nnxnp |
| Marker2855 | AAATTTCATCCAATCAAACTATCGCTTATATTAGAAGAAAXXXXXXXXXXCAGCAGAAATCTTGATGTTCCAGATGTGCGATTTCGTAGA | nnxnp |
| Marker2863 | TGGTCCAAAAATGGTTGCAATTGGGTTATTGGTTTGTAGGXXXXXXXXXXAAGAAAACTCTGAACCCTAAACAAATTACAAAGAACAAAC | nnxnp |
| Marker2863 | TGGTCCAAAAATGGTTGCAATTGGGTTATTGGTTTGTGGGXXXXXXXXXXAAGAAAACTCTGAACCCTAAACAAATTACAAAGAACAAAC | nnxnp |
| Marker2874 | GTAGGGGGGATAGGCGAAAATTCCAAGTCGTGTTGTGTTTXXXXXXXXXXGAATTTTGAAATTTTGATAAACTTCTGGATAAGAAAATAT | lmxll |
| Marker2874 | GTAGGGGGGATAGGCGAAAATTCCAAGTCGTGTTGTGTTTXXXXXXXXXXAAATTTTGAAATTTTGATAAACTTCTGGATAAGAAAATAT | lmxll |
| Marker2875 | TAACCGACGTCGTAGACCGAATTCAATTAGTCTTGGTTTAXXXXXXXXXXTCATTTGAACTTTGAAAACTAAAATTACATTTTTCCGGCC | nnxnp |
| Marker2875 | TAACCGACGTCGTAGACCGAATTCAATTAGTCTTGGTTTAXXXXXXXXXXTCATTTGAACTTTGAAAACTAAAATTACATTTTTCAGGCC | nnxnp |
| Marker2876 | ACAAACTTGTGAAAGATAGAGAAAACAAGATCCAATAATTXXXXXXXXXXCCGGAAACAACGTCTGCAACAACAACAATCACCATGAACA | lmxll |
| Marker2876 | ACAAACTTGTGAAAGATAGAGAAAACAAGATCCAATAATTXXXXXXXXXXCCGGAAACAACGTCTGCAACAGCAACAATCACCATGAACA | lmxll |
| Marker2883 | ATGAGAAATCGATACAAATCTTGTGATAACTGTAAGATTAXXXXXXXXXXAAAATGCAAGGTTACGAACCACAATTTTCACACCGGCATC | hkxhk |
| Marker2883 | ATGAGAAATTGATACAAATCTTGTGATAACTGTAAGATTAXXXXXXXXXXAAAATGCAAGGTTACGAACCACAATTTTCACACCGGCATC | hkxhk |
| Marker2884 | GATGGAGTAGAACTTACGACTTGTTCTTTCAGCATTGATGXXXXXXXXXXCTAAGCTCTCGATAAATACAGGTGGGAAACGCTACAAAAT | lmxll |
| Marker2884 | GATGGAGTAGAACTTACGACTTGTTCTTTCAGCATTGATGXXXXXXXXXXCTAACCTCTCGATAAACACAGGTGGTAAACGCTACAAAAT | lmxll |
| Marker2886 | TGGGATGAAGCAAAAGGTGCAGGTAAGGCAACACAGCCAAXXXXXXXXXXATCAGCTCCTTGCGTGAGCAGGTGAGAACTCACTTCATCT | nnxnp |
| Marker2886 | TGGGATGAAGCAAAAGGTGCAGGTAAGGCAACACTGCCAAXXXXXXXXXXATCAGCTCCTTGCGTGAGCAGGTGAGAACTCACTTCATCT | nnxnp |
| Marker2890 | TTCCTCCAATTTCATTTTGCTGAAAAAAAAAAATTTGTAGXXXXXXXXXXAGCTTCCTTACAACTTCAAAAATGAAAAGAATGAAAGAGT | efxeg |
| Marker2890 | TTCCTCCAATTTCATTTTGCTGAAAAAATAAAATTTGTAGXXXXXXXXXXAGCTTCCTTACAACTTCAAAAATGAAAAGAATGAAAGAGT | efxeg |
| Marker2891 | GTTCAGGTAGTGCCCTTATCTATTTTCATATGTCATTGTGXXXXXXXXXXGATTTACTGCATGACGTTCAGGTTCTTAGGTGTTAGCATA | lmxll |
| Marker2891 | GTTCAGGTAGTGCCCTTATCTATTTTCATATGTCATTGTGXXXXXXXXXXGAGTTACTGCATGACGTTCAGGTTCTTAGGTGTTAGCATA | lmxll |
| Marker2892 | TTCTGTGAATAATGCTTCCCTTCCCTTTTGCATCTGCGAAXXXXXXXXXXTACAAAAGTTATTTCGCAACTTTATTCTATTTTCTCATTG | lmxll |
| Marker2892 | TTCTGTGAATAATGCTTCCCTTCCCTTTTGCACCTGCGAAXXXXXXXXXXTACAAAAGTTATTTCGCAACTTTATTCTATTTTCTCATTG | lmxll |
| Marker2902 | AAAGAACAATGTGGACACAAAATGGAGTAATGGAGTGTCAXXXXXXXXXXGAAGAAAAAAAACAGAAGAGAAAGCTGGCATTTACAAAAT | lmxll |
| Marker2902 | AAAGAACAATGTGGACACAAAATGGAGTGATGGAGTGTCAXXXXXXXXXXGAGAAAAAAAAACAGAAGAGAAAGCTGGCATTTACAAAAT | lmxll |
| Marker2903 | AAGATCCACACTGGAGTGGGAAAGCCATTTGGAGAAATTGXXXXXXXXXXACAGATGTATTGAGCGATGAATCTGTAAGTACTTTACCAC | hkxhk |
| Marker2903 | AAGATCCACACCGGAGTGGGAAAGCCATTTGGAGAAATTGXXXXXXXXXXACAGATGTATTGAGCGATGAATCTGTAAGTACTTTACCAC | hkxhk |
| Marker2913 | AAACCACGTGTCTGGAAATATAAGCGCCCTGTAGATGGATXXXXXXXXXXCCGAAACGAGAACAAGCAGTGCACTATCTGAGCCGCACCC | nnxnp |
| Marker2913 | AAACCACGTGTCTGGAAATATAAGCGCCCTGTAGATGGATXXXXXXXXXXTCCGAACGAGAACAAGCAGTGCACTATCTGAGCCGCACCC | nnxnp |
| Marker2919 | CACACTAAAATAAGACCGCGACTAAAATATTCATACAAACXXXXXXXXXXGGCACTGGCGGACCAGAATTTTTTTAGAGGAGGTCCAATA | lmxll |
| Marker2919 | CACACTAAATAAGACCGCGACTAAAATATTCATACAAACTXXXXXXXXXXGCACTGGCGGACCCAGAATTTTTTTAGAGGAGGTCCAATA | lmxll |
| Marker2930 | TAAAATAAAATAAAGGTCCTTCACAAAGAATAATTCTAACXXXXXXXXXXTCCAGTGTAAAACTGCATCCCGGGGGCATTGCTCCATAGG | lmxll |
| Marker2930 | TAAAATAAAATAAAAGGTCCTTCACAAAGAATAATTCTAAXXXXXXXXXXTCCAGTGTAAAACTGCATCCCGGGGGCATTGCTCCATAGG | lmxll |
| Marker2937 | TCTTCACCCACAGTTGTTCATTATCAAAGGAATCAACAAGXXXXXXXXXXGTCTTCGCATTTTTTCCAGAATATCTTTTTATGTAGGAAC | efxeg |
| Marker2937 | TCTTCACCCACAGTTGTTCATTATCCAAGGAATCAACAAGXXXXXXXXXXGTCTTCGCATTTTTTCCAGAATATCTTTTTATGTAGGAAC | efxeg |
| Marker2948 | TGTTACAACGCCAGAGAATTGGTAAGACAACAACCACAAGXXXXXXXXXXTTTTTATTTTTTGGTCAAACGATAAAGAAAAACTGTGGTG | nnxnp |
| Marker2948 | TGATACAACGCCAGAGAATTGGTAAGACAACAACCACAAGXXXXXXXXXXTTTTTATTTTTTGGTCAAACGATAAAGAAAAACTGTGGTG | nnxnp |
| Marker2949 | TGACAAAAGAAGCATATAATAATCTGGATATACAAAGTGAXXXXXXXXXXTGAAAAAGAATTCAGGATTATCCTGTAACCAAGCACTTAC | lmxll |
| Marker2949 | TGACAAAAGAAGCATATAATAATCTGGATATACACAGTGAXXXXXXXXXXTGAAAAAGAATTCAGGATTATCCTGTAACCAAGCACTTAC | lmxll |
| Marker2950 | CCTAAATTTACAAAGACTGAAAGAGCCAAAAATGATATTCXXXXXXXXXXCACAGAATGTTATGCGGTCCTACCATCTGCAACTGCTATC | lmxll |
| Marker2950 | CCTAAATTTACAAAGACTGAAAGAGCCAAAAATGATATTCXXXXXXXXXXCACAGACTGTTATGCGGTCCTACCATCTGCAACTGCTATC | lmxll |
| Marker2964 | GATTTACTCATCTCAGTGTACATAAAAATGATGGAGAGCCXXXXXXXXXXGCAAATGACCTTCTTTGAGGCTCTTATGGGTGGCAGTCCC | efxeg |
| Marker2964 | GATTTACTCATCTCAGTGTACATAAAAATGATGAAGAGAGXXXXXXXXXXGCAAATGACCTTCTTTGAGGCTCTTATGGGTGGCAGTCCC | efxeg |
| Marker2970 | TATGTCGTCTGAAATGGTTTGAATGGGCAAGCAACTCACAXXXXXXXXXXAATAGTTTTGTCATCTTTGTTCGTGCATGTGCTTTTGTGT | lmxll |
| Marker2970 | TATGTCGTCTGAAATGGTTTGAATGGGCAAGGAACTCACAXXXXXXXXXXAATAGTTTTGTCATCTTTGTTCGTGCATGTGCTTTTGTGT | lmxll |
| Marker2978 | AAATCCTGGAAAAATTGAATTTCCCACTCATGGATTGTTAXXXXXXXXXXTAATAAGCTGTATTGAATGAACAGTAATCAAGATCGGGCA | nnxnp |
| Marker2978 | AAATCCTGGAAAAATTGAATTTCCCACTCACGGATTGTTAXXXXXXXXXXTAATAAGCTGTATTGAATGAACAGTAATCAAGATCGGGCA | nnxnp |
| Marker2981 | CCATCCTGGTCTTCAAAAGCTCACATACAATGCTGAAGCGXXXXXXXXXXAGTGTTAGATTTATTTTAGATTTACCATTGGCTCGGGTCT | lmxll |
| Marker2981 | CCATCCTGGTCTTCAAAAGCTCACATACAACGCTGAAGCGXXXXXXXXXXAGTGTTAGATTTATTTTAGATTTACCATTGGCTCGGGTCT | lmxll |
| Marker2982 | CAAAACAAAAACAAGAAACCAAAGAAAAACACAAACAACAXXXXXXXXXXCCATTTTTATGATTGAAGTTGCCATCTCTCTCCATCTTTA | nnxnp |
| Marker2982 | CAAAACAAAAACAAGAAACCAAAGAAAAACACAAACAGCAXXXXXXXXXXCCATTTTTATGATTGAAGTTGCCATCTCTCTCCATCTTTA | nnxnp |
| Marker2984 | GTGACAATAAAACAGAAGTTCGTTTTGATTTGGTTCACTCXXXXXXXXXXTACTTATGAGGTCATTCTCTTCTCTCTGCAGTATGTGTGA | nnxnp |
| Marker2984 | GTGACAATAAAGCAGAAGTTCGTTTTGATTTGGTTCACTCXXXXXXXXXXTACTTATGAGGTCATTCTCTTCTCTCTGCAGTATGTGTGA | nnxnp |
| Marker2997 | TGTTTGGGTTGAGTGCATATTTCCTTGGCCCAGTCATTTAXXXXXXXXXXATGTGGGACAAGAGGAAATTGAACTTCAACAATACGAGCT | nnxnp |
| Marker2997 | TGTTTGGGTTGAGTGTATATTTCCTTGGCCCAATCATTTAXXXXXXXXXXATGTGGGACAAGAGGAAATTGAACTTCAACAATACGAGCT | nnxnp |
| Marker2998 | GTTACTATTGTAAGTGTTGGCATCATCAAGTGTGGCATCTXXXXXXXXXXGGAGTCAAGAATAATGGCTGAAGAGTGTGGAGTAGAGACT | lmxll |
| Marker2998 | GTTACTATTGTAAGTGTTGGCATTATCAAGTGTGGCATCTXXXXXXXXXXGGAGTCAAGAATAATGGCTGAAGAGTGTGGAGTAGAGACT | lmxll |
| Marker3003 | ACCTATAGCATGAAGGATATCTCTAAGTGTTTCTTGATGGXXXXXXXXXXCCTATAGATATTCCAATTACCACCTCAGATACATAGCCCA | lmxll |
| Marker3003 | ACCTATAGCATGAAGGATATCTCTAAGTGTTTCTTGATGGXXXXXXXXXXCCTATAGATATTCCAATTACCACCCCAGATACATAGCCCA | lmxll |
| Marker3006 | CCCTCATCATTGGTCATCATGAAGAGGTAATTATTAGATGXXXXXXXXXXAAAGGGAACATAGCTCAGATGTCCTTACAAGGCGACCTAA | nnxnp |
| Marker3006 | CCCTCATCAGCGGTCATCATGAGGAGGTAATTATTAGATGXXXXXXXXXXAAAGGGAACATAGCTCAGATGTCCTTACAAGGCGACCTAA | nnxnp |
| Marker3010 | TTATAGAGCAATGTGTAATGGGAGGCCAAAAGCAAACATGXXXXXXXXXXATCAACAGTTGATAGTCATTTATGTGCGTGAATATGAACG | nnxnp |
| Marker3010 | TCATAGAGCAATGTGTAATGGGAGGCCAAAAGCAAACATGXXXXXXXXXXATCAACAGTTGATAGTCATTTATGTGCGTGAATATGAACG | nnxnp |
| Marker3013 | AGAGATATGAGGAACCAATATCTTATTCGACCAAAACAATXXXXXXXXXXCATGCCCTGCCCTTACTTTTCTCTTGTTCTTCTTTCTTTG | nnxnp |
| Marker3013 | AGAGATATGAGGAACCAATATCTTATTCGACCAAAACAATXXXXXXXXXXCATGCCCTGCCCTTACGTTTCTCTTGTTCTTCTTTCTTTG | nnxnp |
| Marker3019 | TACTTTACTTGTTCCATCTTTATCCAATAAATTGATGTCCXXXXXXXXXXCTATCCGTTGAGTACAAACAAGTGTACTACTATGTTTACG | lmxll |
| Marker3019 | TACTTTACTTGTTCCATCTTTATCCAATAAATTGATGTCCXXXXXXXXXXCTATCCATTGAGTACGAACAAGTGTACTACTATGTTTACG | lmxll |
| Marker3032 | ATCTTTGTCTTGCATGCATACAGTTTGTCCAAACTTCCATXXXXXXXXXXTGATTTTTCCATCAACTTGCCCCGGAAACTTTTGCTGGGT | lmxll |
| Marker3032 | ATCTTTGTCTTGCATGCATGCAGTTTGTCCAAACTTCCATXXXXXXXXXXTGATTTTTCCATCAACTTGCCCCGGAAACTTTTGCTGGGT | lmxll |
| Marker3033 | GTTCGCTTGAATAAGTATCGCGTTTGTTTGAAAAAATAAGXXXXXXXXXXACGAGTAACTTTACCCAACGAATGCCCCTCTTGTGTTCGC | nnxnp |
| Marker3033 | GTTCGCTTGAATAAGTATCCCGTTTGTTTGAAAAAATAAGXXXXXXXXXXACAAGTAACTTTACCCAACGAATGCCCCTCTTGTGTTCGC | nnxnp |
| Marker3035 | TTTTCCCCCCTCTTCAAATGCTAAATTGTGTTATTGGGATXXXXXXXXXXTTTGAGGACATTTTTTTGTCATGTTTATGGCCTAATAGGT | lmxll |
| Marker3035 | TTTTTCCCCCTCTTCAAATGCTAAATTGTGTTATTGGGATXXXXXXXXXXTTTGAGGACATTTTTTTGTCATGTTTATGGCCTAATAGGT | lmxll |
| Marker3037 | GTCGTCAGTTTTTGTGTGTATGTGCGTGAGAGAAATAGAGXXXXXXXXXXTCTGTAAAATCACCAGTTTCGTAGTAAATAGGCCGACTCG | hkxhk |
| Marker3037 | GTCGTCAGTTTTTGTGTGTATGTGCGTGAGAGAAATAGAGXXXXXXXXXXTCTGTAAAATCACCAGTTTCGTAGTAAACAGGCCGACTCG | hkxhk |
| Marker3046 | CAACTTATAGGGAATCAGTATCATTCCTACCCCGATTCCAXXXXXXXXXXAAGAATATGGTACATAGCTCACCAACTCCAATTACCAATA | nnxnp |
| Marker3046 | CAACTTATAGGGAATCAATATCATTCCTACCCCGATTCCAXXXXXXXXXXAAGAATAGGGTACATAGCTCACCAACTCCAATTACCAATA | nnxnp |
| Marker3047 | TGAGTAGCATTCAATATCTGACATCATCCTACGTCACTGCXXXXXXXXXXGGGTAATTTTGTGGGTTCCACACCCATTGAGGCAGTGTTT | nnxnp |
| Marker3047 | TGAGTAGCATTCATTATCTGACATCATCCTACGTCACTGCXXXXXXXXXXGGGTAATTTTGTGGGTTCCACACCCATTGAGGCAGTGTTT | nnxnp |
| Marker3048 | GAAGAGAAGAATAAAAGAATTTATACATATGCGCTGAAGTXXXXXXXXXXTACAAGGGGAGATGCAAATCACCACACATATACAGCAAAC | lmxll |
| Marker3048 | GAAGAGAAGAATAAAAGAATTTATACATATGCACTGAAGTXXXXXXXXXXTACAAGGGGAGATGCAAATCACCACACATATACAGCAAAC | lmxll |
| Marker3049 | ATGGTTATGGTGATTTCATGAATGCAGACAATGAAATCTTXXXXXXXXXXTATCATGACATACAAGTGTTGTTTCTGGAGTTTCAATTAT | lmxll |
| Marker3049 | ATGGTTATGGTGATTTCATGAATGCAGACAATGAAATCTTXXXXXXXXXXTATCATGACATACAAGTTTTGTTTCAGGAGTTTCAATTAT | lmxll |
| Marker3051 | CTGCCTCTATTACTCTATCCATTACAAATGCATCGCTTCTXXXXXXXXXXAACTACAAGTTCAGGGATTAGAGGGTTGTAATGTTTTCGT | lmxll |
| Marker3051 | CTGCCTCTATTACTCTATCCATTACAAATGCATGGCTTCTXXXXXXXXXXAACTACAAGTTCAGGGATTAGAGGGTTGTAATGTTTTCGT | lmxll |
| Marker3052 | TGAAATGGGTTATGGTAAAACAATAATGGAGAGAAATGCCXXXXXXXXXXCATATCTGATCCTCCACTCATCAATTGACACGTGGATCAC | lmxll |
| Marker3052 | TGAAATGGGTTATGGTAAAACAATAATGGAGAGAAATGCCXXXXXXXXXXCATATATGATCCTCCACTCATCAATTGACACGTGGATCAC | lmxll |
| Marker3053 | AAATCATTGATGGGGAACTAAACGAGGCCAGAAAGGTTGCXXXXXXXXXXGAAGAACATGATTAGGAGTATTTATTTGTTCATTCAATAT | lmxll |
| Marker3053 | AAATCATTGATGGGGAACTAAACGAGGGCAGAAAGGTTGCXXXXXXXXXXGAAGAACATGATTAGGAGTATTTATTTGTTCATTCAATAT | lmxll |
| Marker3055 | TGAATTCTCTTTCTAAAAAAAAAAGTTCGGATATGAATATXXXXXXXXXXGCCCGTCAACAACGAAGGGAGGTTTTGCAATTTCCATGCC | lmxll |
| Marker3055 | TGAATTCTCTTTCTAAAAAAAACAGTTCGGATATGAATATXXXXXXXXXXGCCCGTCAACAACGAAGGGAGGTTTTGCAATTTCCATGCC | lmxll |
| Marker3058 | TCCTACCATCGTGCCCTAACTCCTTTGCCATCACATCTATXXXXXXXXXXTGGTTGAAGGACAACATACAAAAGCCAAAGTTTACTTCCG | lmxll |
| Marker3058 | TCCTACCATCGTGCCCTAACTCCTTTGCCATCGCATCTATXXXXXXXXXXTGGTTGAAGGACAACATACAAAAGCCAAAGTTTACTTCCG | lmxll |
| Marker3060 | AATTATATAATTACAATGGACAACCCTTTATCATAGTTTTXXXXXXXXXXTTTTGTATGAATGGAGATATTTTCACAGACCTCGCCTAAC | nnxnp |
| Marker3060 | AATTATATAATTACAATGGACAACCCTTTATCATAGTTTTXXXXXXXXXXTTTTGTATGAAGGGAGATATTTTCACAGACCTCGCCTAAC | nnxnp |
| Marker3065 | TGAGACCCCTCCCATTTGCTTAGCCATAGCTTCTTGATCAXXXXXXXXXXTTGGTGTTTTGGCTTTCCTTATGTGCTCTAACATTTCTTC | lmxll |
| Marker3065 | TGAGACCCCTCCCATTTGCTTAGCCATAGCTTCTTGATCAXXXXXXXXXXTTGGTGTTTTGGCTTTCCTTATGTGCTCTAACATTTTTTC | lmxll |
| Marker3066 | AGCAACATGATGTTACACTTGCCCTTATGACAAGACGAGCXXXXXXXXXXCGCAACTATTGACTTGTGAAGGATGGGAAAGAGTTCCGCA | nnxnp |
| Marker3066 | AGCAACATGATGTTACGCTTGCCCTTATGACAAGACGAGCXXXXXXXXXXCACAACTATTGACTCGTGAAGGATGGGAAAGAGTTCCGCA | nnxnp |
| Marker3077 | GGTAGCTGGGAGGACTCTGGCTTGACTTGGGCTTGGTTGGXXXXXXXXXXCTAATTTCCATTTATATATATGTGGAGAAATAAAATGGAG | lmxll |
| Marker3077 | GGTAGCTGGGAGGACTTTGGCTTGAATTGGGCTTGGTTGGXXXXXXXXXXCTAATTTCCATTTATATATATGTGGAGAAATAAAACGGAG | lmxll |
| Marker3080 | TCATCTAAGTTGTTGTATGTGACACACTATAATTCTGAATXXXXXXXXXXTATTTCTGTCTCTTCCCAAAACCAAGATGTATCGTGCTCC | lmxll |
| Marker3080 | TCATCTAAGTTGTTGTATGTGACACACTATAATTCTGAATXXXXXXXXXXTATTTCTGTCTCTTCCCAAAACCAAGATGCATCGTGCTCC | lmxll |
| Marker3083 | AATTTGTGTTCATATTTTTTGTGAAAAGATTCTTCCAAGAXXXXXXXXXXAGAACAAAACAAGAGGAAAATATGAAGTAAAAAATGCATG | nnxnp |
| Marker3083 | AATTTGTGTTCATATTTTTTGTGAAAAGATTCTTCCAAGAXXXXXXXXXXAGAACAAAACAAGAGGAAAATATGAAGTCAAAAATGCATG | nnxnp |
| Marker3088 | TTGTGAACTATTCCACAGCTACTCTACCATTGCCTGGACAXXXXXXXXXXGTTATCCATGTCATCGGAAGTTATCAGGTACAATACTTAT | lmxll |
| Marker3088 | TTGTGAACTATTCCACAGCTACTCTACCGTTGCCTGGACAXXXXXXXXXXGTTATCCATGTCATTGGAAGTTATCAGGTACAATACTTAT | lmxll |
| Marker3095 | GGAATGATGCTTACGAAAACTCTCGAATTTACAAAGAGAAXXXXXXXXXXCTTATGCCCAAATTTGTGGAGGAGGTTGCGTGGAGAACAG | nnxnp |
| Marker3095 | GGAATGATGCTTACGAAAACTCTCGAATTTACAAAGAGAAXXXXXXXXXXCTTATGCCCAGATTCGTGGAGGAGGTTGCGTGGAGAACAG | nnxnp |
| Marker3100 | AATGTAAGCTATATTGTCAATTTGGTATAATTCAAACTCAXXXXXXXXXXTGGAGGAAAATGGCAAATTGACACAATTCATTTGCAAGCA | lmxll |
| Marker3100 | AATGTAAGCTATATTGTCAATTTGGTATCATTCAAACTCAXXXXXXXXXXTGGAGGAAAATGGCAAATTGACACAATTCATTTGCAAGCA | lmxll |
| Marker3107 | CGGCATCGTAGAGAGCGTTCAAGCCAGAGAATGTACAACCXXXXXXXXXXTTTTTATTTTGCTTACAATGTACAATTTAGGGTTGCCATC | lmxll |
| Marker3107 | CGGCATCGTAGAGAGCGTTCAAGCCAGAGAATGTACAACCXXXXXXXXXXTTTTTATTTTGCTTACAATGTACAATTTAGGGTAGCCATC | lmxll |
| Marker3110 | CTCCTGAATTTTCTTATCCTTCTCATCCTGGTAATATAGAXXXXXXXXXXGTTCAAAAAGGGAAAAATACATGGATTTAGAAAGAATATA | lmxll |
| Marker3110 | CTCCTGAATTTTCTTATCCTTCTCATCCTGGTAATATAGAXXXXXXXXXXGTTCAAAAAGGGAAAAATACATGGATTTATAAAGAATATA | lmxll |
| Marker3111 | AAATGAAGAACAGGTTGATGGTTTGATATCCCCTTTTGTAXXXXXXXXXXCATCTGATTGTGTTGATACAAGTAAACTGTCATATTTCAT | lmxll |
| Marker3111 | AAATGAAGAACAGGTTGATGGTTTGATATCCCCTTTTGTAXXXXXXXXXXTCTGATCTTGTGTTGATACAAGTAAACTGTCATATTTCAT | lmxll |
| Marker3114 | TAGCACTCTTTCTTCTTTGCTCATAGTAATCTGGGCCAGAXXXXXXXXXXTTATTTTCTGGAACCATTCCTCCGGAGCTGGGGAAGTTGA | lmxll |
| Marker3114 | TAGCACTCTTTCTTCTTTGCTCATAGTAATCTGGGCCAGAXXXXXXXXXXTTATTTTCTGGAACCATTCCTCCAGAGCTGGGGAAGTTGA | lmxll |
| Marker3115 | GCATAGCAAGACATATTGGAGATAGTGTGGCCGAGCCGAGXXXXXXXXXXGGGCCGAGCACATATATGGAGATAATAATTTTCCCTCTCT | lmxll |
| Marker3115 | GCATAGCAAGACATATTGGAGATAGTGTTGCCGAGCCGAGXXXXXXXXXXGGGCCGAGCACATATATGGAGATAATAATTTTCCCTCTCT | lmxll |
| Marker3119 | ATCAGCCAAACCCACCATGCAATGCATTTCCTTCCAATATXXXXXXXXXXGTCTAAAAGAAGGCGCAAATTAGGAAAATAATTACCATAA | lmxll |
| Marker3119 | ATCAGCCAAACCCACCATGCAATGCATTTCCTTCCAATGTXXXXXXXXXXGTCTAAAAGAAGGCGCAAATTAGGAAAATAATTACCATAA | lmxll |
| Marker3134 | AAACTGTAAACTAAAACATATGTAACAATAAAAAGAAAATXXXXXXXXXXAACCAATTTGATCCAATAACTAACTAATTTGATCCAACTA | lmxll |
| Marker3134 | AACTGTAAACTAAAACATATGTACCAATAAAAAGAAAATTXXXXXXXXXXAACCAATTTGATCTAATAACTAACTAATTTGATCCAACTA | lmxll |
| Marker3138 | AAGGAAAAAGTGTTGTATTATTATTATATATCTATTGCGTXXXXXXXXXXTCCTTAGCCAGAGAAGTATCAGCTGTTCAATTTTCCATTT | lmxll |
| Marker3138 | AAGGAAAAAGTGTTGTATTATTATTATATATCTATTGCGTXXXXXXXXXXTCCTTAGCCAGATAAGTATCAGCTGTTCAATTTTCCATTT | lmxll |
| Marker3147 | TATAACCGACATGGAATATAAGATTAGTTTCTAAATAAAGXXXXXXXXXXTTATGATGAGGAATAGAATTCATTCTGGTAAGACTTCACT | lmxll |
| Marker3147 | TATAACCGACATGGAATATAAGATTAGTTTCTAAATAAAGXXXXXXXXXXTTATGATGAGGAACAGAATTCATTCTGGTAAGACTTCACT | lmxll |
| Marker3150 | GTCTGCACAACAACCACTGACAGTGACCTTGGTGGCGTGCXXXXXXXXXXTAAGATCTTGATTTTGGTGTTGGAAGTATAAGATGTTTAT | nnxnp |
| Marker3150 | GTCTGCACAACAACCACTGACAGTGACCTTGGTGGCGTGCXXXXXXXXXXTAAGATCTTGATTTTGGTGTTGGAAGTATAAGATATTTAT | nnxnp |
| Marker3152 | GCTTATCAGTGAGGTCCCCATTGGCAACTTGCATAGGGACXXXXXXXXXXTCTGTGTCCATGGACCCAAGCTATCTGGTTTGGCTCCCCC | lmxll |
| Marker3152 | GCTTATCAGTGAGGTCCCCATTGGCAACTTGCATAGGGACXXXXXXXXXXTCTGTGTCCATGGACCCAAGCTGTCTGGTTTGGCTCCCCC | lmxll |
| Marker3153 | AGGTGCAATTTTTCTTACACAGGCAAACCTCACTCACTACXXXXXXXXXXATAGAGGACACTGTAAGTGTTACCTATTTTCCCTCCCATT | nnxnp |
| Marker3153 | AAGGTGCAATTTTTCTTACACAGGCAAACCTCACTCACTAXXXXXXXXXXATAGAGGACACTGTAAGTGTTACCTATTTTCCCTCCCATT | nnxnp |
| Marker3159 | ACAATTATCGTTCAGCATCTGTGATAACTGATAACGTCCAXXXXXXXXXXACCATTATTTCCCTCTGATTACTGTTCTTATTATTGATAC | nnxnp |
| Marker3159 | ACAATTATCGTTCAGCATCTGTGATAACTGATAACGTGCAXXXXXXXXXXACCATTATTTCCCTCTGATTACTGTTCTTATTATTGATAC | nnxnp |
| Marker3170 | CTGTTCTTTCTGATAAAGAAAGTGAATGGGGGTTGGAGGAXXXXXXXXXXAAAAAATGGTCAGAGAAGAACAAAAATACTGGACACTTTA | nnxnp |
| Marker3170 | CTGTTCTTTCTGATAAAGAAAGTGAATGGGGGTTGGAGGAXXXXXXXXXXAAAAAATGGTCAGAGAAGAACAAAAATACTGGACACGTTA | nnxnp |
| Marker3174 | ACACTTCTTGCAACTACTTTCCTCCGAAAGTGGTACTTTGXXXXXXXXXXCACAAGGAAGCTGCCAGAGTTACATTTGATCCACTCAACT | lmxll |
| Marker3174 | ACACTTCTTGCAAGTACTTTCCTCCGAAAGTGGTACTTTGXXXXXXXXXXCACAAGGAAGCTGCCAGAGTAACGTTTGATCCACTCAACT | lmxll |
| Marker3185 | AAAAAAATAAATACTCAGTCATATTTTTCACTTCCCACACXXXXXXXXXXATTTATGAAACGTGAAACACAGCTTTTGCGGTGTGCTAGA | nnxnp |
| Marker3185 | AAAAAAATAAATACTCAATCATATTTTTCACTTCCCACACXXXXXXXXXXATTTATGAAACGTGAAACACAGCTTTTGCGGTGTGCTAGA | nnxnp |
| Marker3190 | TCTAAAAGTGAAGCTCCACCAAAGTTAGAGCTCGGCGGTTXXXXXXXXXXAAATAGGGGACTTACACTTCATTTTTGCAAACTCAATGTC | lmxll |
| Marker3190 | TCTAAAAGTGAAGCTCCACCAAAGTTAGAGCTCGGCGGTTXXXXXXXXXXAAATAGGGGACTTGCACTTCATTTTTGCAAACTCAATGTC | lmxll |
| Marker3193 | TAATCAGTAGTAATAATCATGCAGGGGATGTTACCCATAAXXXXXXXXXXGGTCCTCAAGTACAATGCTCGTCCCTTGAGGTAAGTACTT | hkxhk |
| Marker3193 | TAATCAGTAGTAATAATCATGTAGGGGATGTTACCCATAAXXXXXXXXXXGGTCCTCAAGTACAATGCTCGTCCCTTGAGGTAAGTACTT | hkxhk |
| Marker3201 | TCTGGGGTTTGTAAGAGGATGAACTTTTCTCTTTTGGAAGXXXXXXXXXXAAAGTAAGCAGCTGGTTCAGATGCCACAGAGTACACACTG | nnxnp |
| Marker3201 | TCTGGGGTTTGTAAGAGGATGAACTTTTCTCTTTTGGTGGXXXXXXXXXXAAAGTAAGCAGCTGGTTCAGATGCCACAGAGTACACACTG | nnxnp |
| Marker3208 | TTATATATGTTTGCCAATAGCTATACCTGGTTTTGTAAATXXXXXXXXXXAGCCAAATATTAGGTGAAGAAAACGAATGATTGTTTCATG | lmxll |
| Marker3208 | TTATATATGTTTGCCAATAGCTATACCTGGTTTTGTAAATXXXXXXXXXXACCCAAATATTAGGTGAAGAAAACGAATGATTGTTTCATG | lmxll |
| Marker3211 | CCACACTCAAACCGAGTTGTGTTGGGGTGTGGCCAATTCAXXXXXXXXXXTATTATTCTTTTTATTATTGCTTTTGTGTCTACACTTTAT | lmxll |
| Marker3211 | CCACACTCAAACAGAGTTGTGTTGGGGTGTGGCCAATTCAXXXXXXXXXXTATTATTCTTTTTATTATTGCTTTTGTGTCTACACTTTAT | lmxll |
| Marker3213 | TGGAGTTACTTCAACCGAAAGCATTGATGATGGCAGAATCXXXXXXXXXXTCCCCTTTTCTAAAGTTGAAAAAACCCTTATTCTCCAATG | lmxll |
| Marker3213 | TGGAGTTACTTCAACCGAAAGCATTGATGATGGCAGAATCXXXXXXXXXXTCCCCTTTTCTAAAGCTGAAAAAACTCTTATTCTCCAATG | lmxll |
| Marker3219 | GAAAAAGCAGATGAAGAAGATGGAGAGATGGATAGAAGAGXXXXXXXXXXGCAAATTACATGGAATCGGTCCGGTTCGGGTTTCGCCGGT | nnxnp |
| Marker3219 | GAAAAAGCAAATGAAGAAGATGGAGAGATGGATAGAAGAGXXXXXXXXXXGCAAATTACATGGAATCGGTCCGGTTCGGGTTTCGTCGGT | nnxnp |
| Marker3220 | CACATATTTGGGGCCAAGACACACTAAGAGATTTTATGTCXXXXXXXXXXGGCACAGTCGAATTGTCTTGAACGTAATCTATCTTTTTCC | lmxll |
| Marker3220 | CACATATTTGGGGCCAAGACACACTAAGAGATTTTATGTCXXXXXXXXXXGGCACAGTCAAATTGTCTTGAACGTAATCTATCTTTTTCC | lmxll |
| Marker3221 | ACTACCTGGTTATTTGTCAATACTTGATAAACATCCTTTGXXXXXXXXXXTTGTTTGCTCTTGAAAGGAAGGAATGAGGACCCCATAACT | nnxnp |
| Marker3221 | ACTACCTGGTTATTTGTCAATACTTGATAGACATCCTCTGXXXXXXXXXXTTGTTTACTCTTGAAAGGAAGGAATGAGGACCCCATAACT | nnxnp |
| Marker3222 | GTTACACAGCATATTTTTGTCCCCAGATACAGAGTGCAGTXXXXXXXXXXTGTAAATAAGCAAGTTACAATTTGCAACCCCAATAATTCA | lmxll |
| Marker3222 | GTTACACAGCATATTTTTGTCCCCAGATACAGAGTGCAGTXXXXXXXXXXGTAAAATAAGCAAGTTACAATTTGCAACCCCAATAATTCA | lmxll |
| Marker3223 | ATGGTGTGATTATAGTCTCTGGATGACCATAACCAATTCCXXXXXXXXXXGGGCTACATGGAAAACTTGATAGGCAACAACATTCTACCC | hkxhk |
| Marker3223 | ATGGTGTGATTATAGTCTCTGGATGACCATAACCAATTCCXXXXXXXXXXGGGCTACATGGAAAACTTGATAGACAACAACATTCTACCC | hkxhk |
| Marker3229 | TGATGTTGCTACAACGAAGGTTGAGGAGGCTATAGTGCTTXXXXXXXXXXTACATCAGCATCTTATCAAATTAGCTTGACAAGATATACA | lmxll |
| Marker3229 | TGATGTTGCTACAACGAAGGTTGAGGAGGCTATAGTGCTTXXXXXXXXXXTACATCAGCATCTTATCAAATTAGCTTGACAAGATATGCA | lmxll |
| Marker3234 | ACAAAGAAAACAAGAAACGTCCTTCATCAGACTCTCTTGTXXXXXXXXXXTGAGACCAGGCGCAAACTCGACAAAATGTTGGATGCAATG | lmxll |
| Marker3234 | ACAAAGAAAACAAGAAACGTCCTTCATCAGACTCTCTTGTXXXXXXXXXXTGAGAACAGGCGCAAACTCGACAAAATGTTGGATGCAATG | lmxll |
| Marker3237 | GATATTCCACTATGCATATCTCTGGCAGAGACCGAAAACCXXXXXXXXXXGCCAAAAATCTAAATGTTCCCGTGTAAAAGCAGTATGGGA | lmxll |
| Marker3237 | GAGATTCCACTATGCATATCTCTGGCAGAGACCGAAAACCXXXXXXXXXXGCCAAAAATCTAAATGTTCCCGTGTAAAAGCAGTATGGGA | lmxll |
| Marker3238 | ATGATGAGATAAAGTTGAGAAAGGGGTGGCTTAGTAATTAXXXXXXXXXXGACCGTTGATCACAGCATTCCCAATCTAACCCCACCATTT | lmxll |
| Marker3238 | ATGATGAGATAAAGTTGAGAGGGGGGTGACTTAGTAATTAXXXXXXXXXXGACCGTTGATCACAGCATTCCCAATCTAACCCCACCATTT | lmxll |
| Marker3240 | AAAAATACAATAATGTAAAAAGTTTGTAACATATTACAAAXXXXXXXXXXTCCCACATGCAAACACCCAATTATGCATTCACATAACTCT | nnxnp |
| Marker3240 | AAAAATACAATAATGTAAAAAGTTTGTAACATATTACAAAXXXXXXXXXXTCCCACATGCAAACACGCAATTATGCATTCACATAACTCT | nnxnp |
| Marker3246 | ATACTCATAAAAAAATAATCCAAAAAAAGACTTCAGCCCAXXXXXXXXXXACACAGACCACAAATTCCCACCAAATCCTGTTATTGTTTT | nnxnp |
| Marker3246 | ATACTCATAAAAAAAATAATCCAAAAAAAGACTTCAGCCCXXXXXXXXXXACACAGACCACAAATTCCCACCAAATCCTGTTATTGTTTT | nnxnp |
| Marker3264 | ATGGATTCCCAATTTTAGATCTATGAGAGGCCCATGCGCTXXXXXXXXXXAATTCAATATCTCTTGAATGAGCAGGAGTTATTGGAAACA | nnxnp |
| Marker3264 | ATGGATTCCCAATTTTAGATCTATGAGAGGCCCATGCGCTXXXXXXXXXXAATTCAATATCTCTTGAATGAGTAGGAGTTATTGGAAACA | nnxnp |
| Marker3268 | TTAGTAAGGTATACACTCACCAAGGTCCTCTGCCTTCTTCXXXXXXXXXXCTTGCCTCTGAGTTCGTCTACTATCTCCATAGCCCGAATC | hkxhk |
| Marker3268 | TTAGTAAGGTATACACTTACCAAGGTCCTCTGCCTTCTTCXXXXXXXXXXCTTGCCTCTGAGTTCGTCTACTATCTCCATAGCCCGAATC | hkxhk |
| Marker3273 | CCGGAGCCTGCCCTTGGCTTTTATCCTCCCTTTGAAGCGCXXXXXXXXXXGGAGGAGGGCGTATTGACTGTGGAAACCGAGTAAAGTTAT | lmxll |
| Marker3273 | CCGGAGCCTGCCCTTGGCTTTTATCCTCCCTTTGAAGCGCXXXXXXXXXXGGGGTAGGGCGTATTGACTGTGGAAACCGAGTAAAGTTAT | lmxll |
| Marker3276 | TCCAGCAGTATTGTGCTGAGCCAGGAGAAAATCTGGATTCXXXXXXXXXXATATGAACTTTCTAATACGGCACAAGAATGACATCCAAAC | nnxnp |
| Marker3276 | TCCAGCAGTATTGTGCTGAGCCAGGAGAAAATCTGGATTCXXXXXXXXXXATATGAACTTTCTAATATGGCACAAGAATGACATCCAAAC | nnxnp |
| Marker3285 | TGAAACAAATGGGCACCCAAAATGCAGCTTGTAAAAACTTXXXXXXXXXXGTGACATGAAGGCATGAACTATGAATTCCCATTGAGTAAT | hkxhk |
| Marker3285 | TGAAACAAATGGGCACCCAAAACGCAGCTTGTAAAAACTTXXXXXXXXXXGTGACATGAAGGCATGAACTATGAATTCCCATTGAGTAAT | hkxhk |
| Marker3288 | TAAACAACTTCAATACTTTGATAAATAAAAAAAGTAAACAXXXXXXXXXXCCTCAGCAACCTAATGGCAATGGCATTTCCGCAATAAACA | lmxll |
| Marker3288 | TAAACAACTTCAATACTTTGATAAATAAAAAAAGTAAACAXXXXXXXXXXCCTCAGCAACCTTATGGCAATGGCATTTCCGCAATAAACA | lmxll |
| Marker3293 | CCATAACCCAGACTAATCCCCAATCACACCGGGTCGGCCCXXXXXXXXXXGAGGAGTTTCTCCTTATGGATAAGGAGGGAAGTATCGTCA | lmxll |
| Marker3293 | CCACAACCCAGACTAATCCCCAATCACACCGGGTCGGCCCXXXXXXXXXXGAGGAGTTTCTCCTTATGGATAAGGAGGGAAGTATCGTCA | lmxll |
| Marker3309 | GTAAATATTTTTCTGGGGGGAAACGAATACTTTGTACCCAXXXXXXXXXXGCTGTTTGGGCAAAAATCTTCATGCCTCGAACTGATGGCT | nnxnp |
| Marker3309 | GTAAATATTTTTCTCGGGGGAAACGAATACTTTGTATCCAXXXXXXXXXXGCTGTCTGGGCAAAAATCTTCATGCCTCGAACTGATGGCT | nnxnp |
| Marker3310 | GAATTTTGGCACCTTGCTCGTCCGCCACCTTATCTTCCAAXXXXXXXXXXTGTTAGAAGATACTTCCCCTGGATCATTATGCAGAGGCAT | nnxnp |
| Marker3310 | GAATTTTGGCACCTTGCTCGTCCGCCACCTTATCTTCCAAXXXXXXXXXXTGTTAGAAGATACTTCCCCTGGATCATTATGCAGAGGCGT | nnxnp |
| Marker3311 | CCAGATAAACAAAGGAGAGCAAAGTAAAGAAGTGAGCAGAXXXXXXXXXXTTGTTTGGGGTTGCGTGGAATTGATTTCTTTTAGGGCTGC | lmxll |
| Marker3311 | CCAGATAAACAAAGGAGAGCAAAGTAAGGAAGTGAGCAGAXXXXXXXXXXTTGTTTGGGGTTGCGTGGAATTGATTTCTTTTAGGGCTGC | lmxll |
| Marker3315 | TTGGCACGACCAGTTTTATGGTTGAGCAGCCAAGGTCCAAXXXXXXXXXXTAATGGCGGATACATGAATTTTTCAGGAAAGGTGCAATAT | nnxnp |
| Marker3315 | TTGGCACGACCAGTTTTATGGTTGAGCAGCCAAGGTCCAAXXXXXXXXXXTAATGGCGGATACATGAATTTTTCAGGGAAGGTGCAATAT | nnxnp |
| Marker3318 | ATTCCTTACGAATCCTCCTCGAAAGATTGACATGTCCACTXXXXXXXXXXGATAATAACAATGATTGACAAATATAGTTCACATGTGCGG | lmxll |
| Marker3318 | ATTCCTTACGAATCCTCCACGAAAGATTGACATGTCCACTXXXXXXXXXXGATAATAACAATGATTGACAAATATAGTTCACATGTGCGG | lmxll |
| Marker3326 | GTCTAAGGCCGTACGGAAGGATTACTCCATAAAAGTCATCXXXXXXXXXXAATCATTTCATGGATGTTTGAAATAATTTCATCAACCTTC | nnxnp |
| Marker3326 | GTCTAAGGCCGTACGGAAGGATTACTCCATAAAAGTCATCXXXXXXXXXXAATCATTTCATGGATGTTTGAAATGATTTCATCAACCTTC | nnxnp |
| Marker3334 | TCTCCGTCTTCCCATGCCCGAACCCTCCGAGTGCCGCCCAXXXXXXXXXXGTCATGGAGTACATGGACTCCGGCACCCTCGAGACCTTGC | lmxll |
| Marker3334 | TCTCCGTCTTCCCTTGCCCGAACCCTCCGAGTGCCGCCCAXXXXXXXXXXGTCATGGAGTACATGGACTCCGGCACCCTCGAGACCTTGC | lmxll |
| Marker3335 | TTCCCTCAAAGGATAGCCTCAGATGCACCAAGGGGGAAATXXXXXXXXXXATAACTGCTGGAGCTTATGAATTTGATTTTTGGATCAAGT | lmxll |
| Marker3335 | TCCCCTCAAAGGATAGCCTCAGATGCACCAAGGGGGAAATXXXXXXXXXXATAACTGCTGGAGCTTATGAATTTGATTTTTGGATCAAGT | lmxll |
| Marker3336 | ACATACCCTAAAAACTTCTGCATACAACACACTTTGGTCAXXXXXXXXXXTCATTCGACGTCAAAACGACGTCGTTCCACTTTTGTTTTT | lmxll |
| Marker3336 | ACATACCCTAAAAACTTCTGCATACAACACACTTTGGTCAXXXXXXXXXXTCATTCGACGTCAAAACGACGTTGTTCCACTTTTGTTTTT | lmxll |
| Marker3338 | TACAATAAAATGAAAGTCCAAATCAACTGACCTGAACAAGXXXXXXXXXXTTCTTTTTGGTAAGCACTTCCACTGTTCAGTAAGATACAA | lmxll |
| Marker3338 | TACAATAAAATGAAAGTGCAAATCAACTGACCTGAACAAGXXXXXXXXXXTTCTTTTTGGTAAGCACTTCCACTGTTCAGTAAGATACAA | lmxll |
| Marker3343 | TGGAGATTCTTCGTTGAACATGTCAAGATGTTGCATGACGXXXXXXXXXXTAATGATTTGAAAACTATATAAAATAGGAAAGTAAATCCC | lmxll |
| Marker3343 | TGGAGATTCTTCGTTGAACATGTCAAGATGTTGCATGACGXXXXXXXXXXTAATGATTTGACAACTATATAAAATAGGAAAGTAAATCCC | lmxll |
| Marker3348 | GTTGAATGAAGCTTTTTTTTCGTCATTTTATTTTCAATTTXXXXXXXXXXGTCAAGTGGGTGAGCTTTCGAATTCCAGCAACAAGTATAA | lmxll |
| Marker3348 | GTTGAATGAAGCATTTTTTTCGTCATTTTATTTTCAATTTXXXXXXXXXXGTCAAGTGGGTGAGCTTTCGAATTCCAGCAACAAGTATAA | lmxll |
| Marker3353 | AGCATAAAGGTAAGCTAAATGCGTTTTTTCTTCTTTCTCTXXXXXXXXXXTGCATATATTCGCTCTAAGCTATGAAGTCGAAAGGAAAAA | nnxnp |
| Marker3353 | AGCATAAAGGTAAGCTAAATGCGTTTTTTCTTCTTTCTCTXXXXXXXXXXTGCATATATTCGCACTAAGCTATGAAGTCGAAAGGAAAAA | nnxnp |
| Marker3355 | CACAACCCACAACCTTCAGAGAGCAAGCTTCCTCTCTTGCXXXXXXXXXXCTTCCTAAGGCCTTGTTGCCCTTTCGAAGCATTTTGGGGC | lmxll |
| Marker3355 | CACAACCCACAACCTTCAGAGAGCAAGCTTCCTCTCTTGCXXXXXXXXXXCTTCCTAAGGCCTTGCTGCCCTTTTGAAGCATTTTGGGGC | lmxll |
| Marker3361 | TATTCTTCCTAATTTGGGGAGCTGAAGAAACTAAAAATCTXXXXXXXXXXGGTGGGTCATATCCCCAGCCAACCTGAACGCATTCATGGT | lmxll |
| Marker3361 | TATTCTTCCTAATTTGGGGAGCTGAAGAAACTAAAAATCTXXXXXXXXXXGGTGGGTCATATCCCCGGCCAACCTGAACGCATTCATGGT | lmxll |
| Marker3363 | TAAAAATATTTGCCGCATTGGTTCAAAAAGCAAAAAAAGAXXXXXXXXXXTCTCTCTCTCTCTCTCTCTCTCTCTCTCTCTCATTAGATG | lmxll |
| Marker3363 | TAAAAATATTTGCAGCATTGGTTCAAAAAGCAAAAAAAGAXXXXXXXXXXACTCTCTCTCTCTCTCTTTCTCTCTCTCTCTCATTAGATG | lmxll |
| Marker3365 | ATACTCAAAATCTTTTTTTGTTTTGTTTCATTTGCTCTTAXXXXXXXXXXAACCGTACTTGATCAAACACGAGGGAAACCAGCATTGTGG | nnxnp |
| Marker3365 | ATACTCAAAATCTTTTTTTGTTTTGTTTCATTTGCTCTTAXXXXXXXXXXAACCGTACCTGATCAAACACGAGGGAAACCAGCATTGTGG | nnxnp |
| Marker3368 | AGGTCTCACACAACCAAAGATCAAGAATCCAGCTATCCAAXXXXXXXXXXCCCAGGGTCATGTTGAGTCAGCCAAGTATCAGACCATATA | nnxnp |
| Marker3368 | AGGTCTCACACAACCAAAGATCAAGAATCCAGCTATCCAAXXXXXXXXXXCCCAGGGTCATGTTGAGGCAGCCAAGTATCAGACCATATA | nnxnp |
| Marker3379 | AAAACCAAGCCAAAACAATATAAATAAAACTCTCACAAATXXXXXXXXXXGGAAAAGAAGAGGGGAGGAAGAACAACGGCTTCCACCCCC | nnxnp |
| Marker3379 | AAAAACCAAGCCAAAACAATACAAATAAAACTCTCACAAAXXXXXXXXXXGGAAAAGAAGAGGGGAGGAAGAACAACGGCTTCCACCCCC | nnxnp |
| Marker3385 | TGAATGTTAGCTGTGTTGCCTTAGAGAATCCGAAAAGATAXXXXXXXXXXCGTAATTATTGAGGAGGAAGGAGATGCAAAAGGGTAGTTT | lmxll |
| Marker3385 | TGAATGTTAGCTGTGTTGCCTTGGAGAATCCGAAAAGATAXXXXXXXXXXCGTAATTATTGAGGAGGAAGGAGATGCAAAAGGGTAGTTT | lmxll |
| Marker3386 | GTGCTTGACATTTGGTTTCTTCCCTTTCCAAATTTCATATXXXXXXXXXXTTTTTCATTCATGATCAGTTTGTACAAGACATGAAAGGAA | lmxll |
| Marker3386 | GTGCTTGACATTTGGTTTCTTCCCTTTCCAAATTTCATACXXXXXXXXXXTTTTTCATTCATGATCAGTTTGTACAAGACATGAAAGGAA | lmxll |
| Marker3392 | TCCTGAGGCTAATTCTGTATGTGATTCAGATCTGAGTTTTXXXXXXXXXXTTTGAACTGAAGACTGATTTGAGAACCCTCTGATATGAAT | lmxll |
| Marker3392 | TCCTGAGGCTAATTCTGTATGTGATTCAGATCTGAGTCTTXXXXXXXXXXTTTGAACTGAAGACTGATTTGAGAACCCTCTGATATGAAT | lmxll |
| Marker3396 | AATGCAGGCAATTTGACCTTCCAGTAGATGCAGTGCCACCXXXXXXXXXXAAGCAGTGAAGCTAAAGTGCCAGATAATTTCTCGAAGCCT | lmxll |
| Marker3396 | AATGCAGGCAATTTGACCTTCCAGTAGATCCAGTGCCACCXXXXXXXXXXAAGCAGTGAAGCTAAAGTGCCAGATAATTTCTTGAAGCCT | lmxll |
| Marker3399 | TGAACACTTCCAACTTGGAGCAATCATAGAAAAACTGACTXXXXXXXXXXGACCAAAATCCAACCAATCCTCAAGTCAATGTGACTGAAG | lmxll |
| Marker3399 | TGAACACTTCCAACTTGGAGCAATCATAGAAAAACTGACTXXXXXXXXXXGACCAAAATCCAACCAATCCTCAAGTCAACGTGACTGAAG | lmxll |
| Marker3406 | AACCAGGAGGGCTGTCACTCCCAAACTCTGGCATGGATCCXXXXXXXXXXATTTATATGGTTTAGATGATTGTTTAGCGTATAGCATGTA | lmxll |
| Marker3406 | AACCAGGAGGGCTGTCACTCCCAAACTCTGGCATGGATCCXXXXXXXXXXATTTATATGGTTTAGATGATTGTTTAGCGTATAGCGTGTA | lmxll |
| Marker3421 | GTTATCGGAATTTTTTGGGGTTGATGATTATGAGTCTCAGXXXXXXXXXXGGTTTTCAATTGTGTGCTAAGTTCATAGCTTTCTCCTTTA | lmxll |
| Marker3421 | GTTATCGGAATTTTTTGGGGTTGATGATTATGAGTCTCAGXXXXXXXXXXGGTTTTCAATTGTGTGCTAAGTTCATAGCTTCCTCCTTTA | lmxll |
| Marker3422 | CCTTTTAGTAATTTGCAACTGGGCTTCTGTAATAAAGAATXXXXXXXXXXAGGAATAGAATTTTGTCAACTTTAGTGAAGCTGTACACTT | nnxnp |
| Marker3422 | CCTTTTAGTAATTTGCAACTGGGGTTCTGTAATAAAGAATXXXXXXXXXXAGGAATAGAATTTTGTCAACTTTAGTGAAGCTGTACACTT | nnxnp |
| Marker3424 | TCGGGGATAGGGTATAAGGCTGCAGGATGTTACGGCATCAXXXXXXXXXXTCTGCCGGCGCTGGATTCAGAAAATTGATAAGCAAAGCCT | nnxnp |
| Marker3424 | TCGGGGATAGGGTATAGGCTGCAGGATGTTACGGCATCAGXXXXXXXXXXTCTGCCGGCGCTGGATTCAGAAAATTGATAAGCAAAGCCT | nnxnp |
| Marker3425 | CATGTGTGCAGATGTGCAGAATTTTTTTGTTATATGTTTTXXXXXXXXXXGATGCGACTTTAGAACGCAACCACACATAAGGACAAGTTA | lmxll |
| Marker3425 | CATGTGTGCAGATGTGCAGAATTTTTTTGTTATATGTTTTXXXXXXXXXXGATGCGACTTTAGAATGCAACCACACATAAGGACAAGTTA | lmxll |
| Marker3427 | TCTAACCCCATAATCATGTGTAGTACCAAACATATCATTGXXXXXXXXXXGATGACAACCAAACAAACACAAAAATTCGACATCCAAGAC | lmxll |
| Marker3427 | TCTAACCCCATAATCATGTGTAGTACCAAACATATCATTGXXXXXXXXXXGATGACAACCAAACAAACACAAAAATTCCACATCCAAGAC | lmxll |
| Marker3437 | GGGATTTACTTTCCTATTTTATATAGTTGTCAAATCATTGXXXXXXXXXXATCTTGTACAAAGTGTTGCTAATAAAAGCCATTTCTCAAT | lmxll |
| Marker3437 | GGGATTTACTTTCCTATTTTATATAGTTGTCAAATCATTGXXXXXXXXXXATCTTGTACAAAGAGTTGCTAATAAAAGCCATTTCTCAAT | lmxll |
| Marker3438 | CACCATTTAGATCTGTCATCATCTATGTGCATCAATTTCCXXXXXXXXXXCAAATTACCCTGTAATAATCATATTTGATAGTTTTCCATA | lmxll |
| Marker3438 | CACCATTTAGATCTGTCATCATCAATGTGCATCAATTTCCXXXXXXXXXXCAAATTACCCTGTAATAATCATATTTGATAGTTTTCCATA | lmxll |
| Marker3441 | CAAGCTGATCTTGCAATGCAGGTTCGCACACTCTCTGAGAXXXXXXXXXXCGATCGGTAAACTCAAACTCAATGTTGAGAACACTGTAAA | hkxhk |
| Marker3441 | CAAACTGATCTTGCAATGCAGGTTCGCACACTCTCTGAGAXXXXXXXXXXCGATCGGTAAACTCAAACTCAATGTTGAGAACACTGTAAA | hkxhk |
| Marker3447 | CTTTCAATTTCAGTTTGAATAGACTAAGGCACTAAGATCTXXXXXXXXXXGAAACTCACAGGACTCTGTTTGTGGTTTTGGGCTTACAGA | lmxll |
| Marker3447 | CTTTCAATTTCAGTTTGAATAGACTATGGCACTAAGATCTXXXXXXXXXXGAAACTCACAGGACTCTGTTTGTGGTTTTGGGCTTACAGA | lmxll |
| Marker3449 | GCTTCATGTACCTCAAGCTCTGCAAATGCCATTTCTGGCTXXXXXXXXXXCTTGGCAGATTCTACGTCTACTTCTGATGGAGGAGGGTTC | lmxll |
| Marker3449 | GCTTCACGTACCTCAAGCTCTGCAAATGCCATTTCTGGCTXXXXXXXXXXCTTGGCAGATTCTACGTCTACTTCTGATGGAGGAGGGTTC | lmxll |
| Marker3452 | TATTATTATCTCTCTCTCTCTCTCTCTCTCTCTCTCTCTCXXXXXXXXXXATATAATACGCCATTCTTTTTGTTTATTGGTGCTTGTGTT | nnxnp |
| Marker3452 | TATTATTATCTCTCTCTCTCTCTCTCTCTCTCTCTCTCAGXXXXXXXXXXATATAATACGCCATTCTTTTTGTTTATTGGTGCTTGTGTT | nnxnp |
| Marker3455 | AGCATACCCATTTAGCTTTACCAAAGTACGCATAATCAACXXXXXXXXXXACGGTTTATGGGTTTAGGGTTCAGGGTTTATGCTCAATGA | lmxll |
| Marker3455 | AGCATACCCATTTAGCTTTACCAAAGTACGCATAATCAACXXXXXXXXXXATGGTTTATGGGTTTAGGGTTCAGGGTTTATGCTCAATGA | lmxll |
| Marker3461 | ACGTGGGTTGAGCTAAAAAATAATGCAACTTGTTTGTGCTXXXXXXXXXXAAACTCTTCTTCCTTGCTCTCTCTTTTTCAAATGATAGCT | lmxll |
| Marker3461 | ACGTGGGTTGAGCTAAAAAATAATGCAACTTGTTTGTGCTXXXXXXXXXXAAACTCTTCTTCCTTGCTCTCTCTTTTGCAAATGATAGCT | lmxll |
| Marker3465 | AGTGTGTGCAAAGTTAGATAGAAGGAGAGGGACAACTCCTXXXXXXXXXXGAAAGAAAACATATCAAGCATGCCAAAATCTATAGTGTAA | lmxll |
| Marker3465 | AGTGTGTGCAAAGTTAGATAGAAGGAGAGGGACAACTCCTXXXXXXXXXXAAAAGAAAACATATCAAGCATGCCAAAATCTATAGTGTAA | lmxll |
| Marker3476 | ATCAAACTCCATGCTAAATTATTGTCTAAAGTGCCTCAAAXXXXXXXXXXTAAATATATTTGTTAGTAATTGATTTTCTTACAATTTTAG | lmxll |
| Marker3476 | ATCAAACTCCATGCTAAATTATTGTCTAAAGTGCCTCAAAXXXXXXXXXXTGAATATATTTGTTAGTAATTGATTTTCTTACAATTTTAG | lmxll |
| Marker3487 | TGAAATCATAGCACATATTTCGTGTGAAATCAAACAACATXXXXXXXXXXGCAAACAATCATAAGAATCCAAAATCATTCAACCATGCAC | lmxll |
| Marker3487 | TGAAATCACAGCACATATTTCGTGTGAAATCAAACAACATXXXXXXXXXXGCAAACAATCATAAGAATCCAAAATCATTCAACCATGCAC | lmxll |
| Marker3490 | TATTTGTGAGGTTCCACAGTGTCATAGGAATTGGCCTTGAXXXXXXXXXXAGGCTGGTACAAAAACCAAGCTCATAAGGGATTGAAGAGT | lmxll |
| Marker3490 | TATTTGTGAGGTTCCACAGTGTCATAGGAATTGGCCCTGAXXXXXXXXXXAGGCTGGTACAAAAACCAAGCTCGTAAGGGATTGAAGAGT | lmxll |
| Marker3494 | TAAAACCAGTTATCACTGTCTGTCAGATGTCTTTCGATTTXXXXXXXXXXCGTCATTATGTTAGATTTTTTTTGTTTAGCTTTATGTATG | lmxll |
| Marker3494 | TAAAACCAGTTATCGCTGTCTGTCAGATGTCTTTCGATTTXXXXXXXXXXCGTCATTACGTTAGATTTTTTTTGTTTAGCTTTATGTATG | lmxll |
| Marker3495 | TGAATAATCTTATTTTGGGTCAGGATGTTATAATGTACCCXXXXXXXXXXATGCGTTCCGTGATCTCATAAGGTCCGATATAACGAGGAC | lmxll |
| Marker3495 | TGAATAATCTTATTTTGGGTCGGGATGTTACAATGTACCCXXXXXXXXXXATGCGTTCCGTGATCTCATAAGGTCCGATATAACGAGGAC | lmxll |
| Marker3502 | GAAGCACATCTTTCCCACAAGTTAGTAGAAATCCATTTTCXXXXXXXXXXGAAAAGGCAAAGGCAAGCTATAATTCATGCATAGCATGAT | lmxll |
| Marker3502 | GAAGCACATCTTTCCCACAAGTTAGTAGAAATACATTTTCXXXXXXXXXXGAAAAGGCAAAGGCAAGCTATAATTCATGCATAGCATGAT | lmxll |
| Marker3503 | TATAATTGCTCTACTGAGCTATTTACTCCATTGATTATTCXXXXXXXXXXCTGACAATAGGGTGTAGATGAGTTTGTGGTTTTCTAAAAC | hkxhk |
| Marker3503 | TATAATTGCTCTACTGAGCTATTTACTCCATTGATTATTCXXXXXXXXXXCTGACAATAGGGTGTAGAGGAGTTTGTGGTTTTCTAAAAC | hkxhk |
| Marker3506 | GTTTCCTATAAAAAAAAGTATGTATTGATAGGTTTACTTTXXXXXXXXXXCGACTGGCCTTTCCTGGAAACCAGAGCTGCATATCATGGG | nnxnp |
| Marker3506 | GTTTCCTATAAAAGAAAAGGTATGTGTTGATAGGTTTACTXXXXXXXXXXCGACTGGCCTTTCCTGGAAACCAGAGCTGCATATCATGGG | nnxnp |
| Marker3515 | ACTCAGTTTCTGCAGTGATGATCCTAATGTGGGATGCATAXXXXXXXXXXATTCTCATTTACTCCTATATAAAGGGCATCCCTGCACTCT | lmxll |
| Marker3515 | ACTCAGTTTCTGCAGTGATGATCGTAATGTGGGATGCATAXXXXXXXXXXATTCTCATTTACTCCTATATAAAGGGCATCCCTGCACTCT | lmxll |
| Marker3521 | TCGCAGTATGGAGAAAACCACCATAGAATTGGCATCGAACXXXXXXXXXXCATTTTTACACATGATTTATCATGTTATTCACCCAATATT | nnxnp |
| Marker3521 | TCGCAGTATGGAGAAAACCACCATAGAATTGGCATCGAACXXXXXXXXXXCATTTTTACACATGATTTATCATGTTATTCACCAAATATT | nnxnp |
| Marker3523 | AATCTTTTGCTCTTTGCTCCATTTATCCATTGTGCTTCATXXXXXXXXXXGTTCGAAGTCTGAACGGCTGCAGGGTTGCAGATCAAATTC | nnxnp |
| Marker3523 | AATCTTTTGCTCTTTGTTCCATTTATCCATTGTGCTTCATXXXXXXXXXXGTTCGAAGTCTGAACGGCTGCAGGGTTGCAGATCAAATTC | nnxnp |
| Marker3524 | CTTTTGGTCGTCCATATTAGCTTACATATATTTCATCTAAXXXXXXXXXXTACTGCAATTCTGCAATGAAATATGACTGACCAGATTGTT | lmxll |
| Marker3524 | CTTTTGGTCGTCCATATTAGCTTACATATATTTCATCTAAXXXXXXXXXXTACTGCAATTCTGCAATGAAATATGACTGGCCAGATTGTT | lmxll |
| Marker3526 | AATTACAGGGACAAAATTGACCAAATTGAAATAACAGGGAXXXXXXXXXXAATCCCATCTTGTTCTTGTCCTGTTGCTGCATGCTGTGTG | nnxnp |
| Marker3526 | AATTACAGGGACAAAATTGACCAAATTGAAATGACAGGGAXXXXXXXXXXAATCCCATCTTGTTCTTGTCCTGTTGCTGCATGCTGTGTG | nnxnp |
| Marker3530 | TATCCAAATGTATTGTGTTCTACATTTTGACTCGTGTTCTXXXXXXXXXXATAACATTTAGGCTGCTGACAAGGTTTATAAGTTGTATAT | nnxnp |
| Marker3530 | TATCCAAATGTATTGTGTTCTACATTTTGACTCGTGTTCTXXXXXXXXXXATAACATTTAGGCTGCTGACAAGGTTTAGAAGTTGTATAT | nnxnp |
| Marker3536 | AGCACATGAAGATTTGAAGGCCTCAATCTGTGACTGGCACXXXXXXXXXXAACGCAGCGTTGTAATAAAATTGTTCTCTTGGGCTGTCAT | lmxll |
| Marker3536 | AGCACATGAAGATTTGAAGGCCTCAATCTGTGACTGGCACXXXXXXXXXXAACGCAGCGTTGTGATAAAATTGTTCTCTTGGGCTGTCAT | lmxll |
| Marker3538 | CTGCAATTGCAATGAACTCTGTAGCTGTTGTAGGATTTTGXXXXXXXXXXAAATAATGACGGCAGCATTTTGTCAAGGGTGATATAATAT | lmxll |
| Marker3538 | CTACAATTGCAATGAACTCTGTAGCTGTTGTAGGATTTTGXXXXXXXXXXATAATGACGGCAGCACATTTTGTCAAGGGTGATATAATAT | lmxll |
| Marker3540 | ACTTGTGAGTAATAAGGGCATTCAATGCGGCATCCAATTTXXXXXXXXXXAAGTTCATGTGTGCACTCCTTTGTTTGCAAAAGGGCATCC | lmxll |
| Marker3540 | ACTTGTGAGTAATAAGGGCATTCAATGCGGCATCCACTTTXXXXXXXXXXAAGTTCATGTGTGCACTCCTTTGTTTGCAAAAGGGCATCC | lmxll |
| Marker3552 | CTAATCGGCATACATGGAATAAAAAATTAGAAAAAAACAAXXXXXXXXXXAAGACTCGTTCTCTGGTTCAATAGGAAATTCATCAGAATA | hkxhk |
| Marker3552 | CTAGTCGGCATACATGGAATAAAAAATTAGAAAAAAACAAXXXXXXXXXXAAGACTCGTTCTCTGGTTCAATAGGAAATTCATCAGAATA | hkxhk |
| Marker3560 | TTCATGAAGAAATGATCATCCTACCTCAGCTTGATGAAGAXXXXXXXXXXAATCAGAACTAATGGCATACAAGCCTATTGATCTGTCAGT | nnxnp |
| Marker3560 | TTCATGAAGAAATGATCATCCTACCTCAGCTTGATGAAGAXXXXXXXXXXAATCAGAACTAATGACATACAAGCCTATTGATCTGTCAGT | nnxnp |
| Marker3562 | TTGGCTAACCCATCACCTTAGGAAAAGCCCAAGCAACCAAXXXXXXXXXXAGAGCTTCCAATGGCTATAAAAGGGAGTGTCTTCTACCCA | lmxll |
| Marker3562 | TTGGCTAACCCATCACCTCAGGAAAAGCCCAAGCAACCAAXXXXXXXXXXAGAGCTTCCAATGGCTATAAAAGGGAGTGTCTTCTACCCA | lmxll |
| Marker3573 | TGAATATTTCATATTCCCCATAAGGCCCCTAATAACTTTCXXXXXXXXXXGGTAAAGCATCTGCAGGCACTGCTGCCGGCGAGAGAAGGT | lmxll |
| Marker3573 | TGAATATTTCATATTCCCCATAAGGCCCCTAATAACTTTCXXXXXXXXXXAGTAAAGCATCTGCAGGCACTGCTGCCGGCGAGAGAAGGT | lmxll |
| Marker3584 | GAGGAAGCACAAGAAGGACATCGCAGCGGAAGTAAGATGTXXXXXXXXXXAGGAATTTGATTTGAGTATGAGAAATTTGAGATAAGGCAT | lmxll |
| Marker3584 | GAGGAAGCACAAGAAGGACATCGCAGCGGAAGTAAGATGTXXXXXXXXXXCGGAATTTGATTTGAGTATGAGAAATTTGAGATAAGGCAT | lmxll |
| Marker3592 | ATCCCACAAACACTCCAATTGGACCATTTGCTTCATTAGTXXXXXXXXXXGAATTATTCATTCTCAATCTATGAACAAACACAATAATGT | nnxnp |
| Marker3592 | ATCCCACAAACACTCCAATTGGACAATTTGCTTCATTAGTXXXXXXXXXXGAATTATTCATTCTCAATCTATGAACAAACACAATAATGT | nnxnp |
| Marker3604 | ACAAAAAACAAAAAACAAAAAACAAAAAAAACAAAAAACAXXXXXXXXXXCGCATGCCCAAATTGGTAGCCGAGACTGCGTGGAGCACAG | lmxll |
| Marker3604 | AAAAAAAACAAAAAACAAAAAACAAAAAAAACAAAAAACAXXXXXXXXXXCGCATGCCCAAATTGGTAGCCGAGACTGCGTGGAGCACAG | lmxll |
| Marker3611 | AGTTGTTTCTTTGTTTTATAATTTTGTAATCTTACGTATTXXXXXXXXXXAGAAACAAAGAATGTGGACAAGTTCTTCACTGGGCTCAAA | lmxll |
| Marker3611 | AGTTGTTTCTTTGTTTTATAATTTTGTAATCTTACGTATTXXXXXXXXXXAGAAACAAAGAATGTAGACAAGTTCTTCACTGGGCTCAAA | lmxll |
| Marker3613 | TGGACTACAGGTCAAGTAAATGAATAAGTATTCTCCAAATXXXXXXXXXXATTACCATGAAAAAGCATGCACTTTTATCAAAGCCAAATA | nnxnp |
| Marker3613 | TGGACTACAGGTCAAGTAAATGAATAAGTATTCTCCAAATXXXXXXXXXXATTACCATGAAAAAGCATGCACTTTCATCAAAGCCAAATA | nnxnp |
| Marker3614 | CGATAGATGCAACAGCCTTACTGTGGAAATCAATGAGGTTXXXXXXXXXXAGGCTTTGCCACATTTTTTGCATCTTTAGAGTGAGCATGA | lmxll |
| Marker3614 | CGATAGATGCAACAGCCTTACTGTGGAAATCAATGAGGTTXXXXXXXXXXAGGCTTTGCCACATTTTTTGCATCTTTAGAGTGAGCACGA | lmxll |
| Marker3622 | AGGACAAAGGACTTTGGGTTAGGGCTTCTTTTATGGTCTTXXXXXXXXXXCCTTTGCCTTGCAGCCACGGTCTGTGGGTCAGATGAGATC | nnxnp |
| Marker3622 | AGGACAAAGGACTTTGGGTTAGGGCTTCTTTTATGATCTTXXXXXXXXXXCCTTTGCCTTGCAGCCACGGTCTGTGGGTCAGATGAGATC | nnxnp |
| Marker3627 | GAGAGGTCAGGGGTTCGATCATTGCCCTCGTATGGAGTCAXXXXXXXXXXATTCTGTTACGATAGGACTCAATTGAAACTACTTGTCGAA | nnxnp |
| Marker3627 | GAGAGGTCAGAGGTTCGATCATTGCCCTCGTATGGAGTCAXXXXXXXXXXATTCTGTTACGATAGGACTCAATTGAAACTACTTGTCGAA | nnxnp |
| Marker3635 | CGAGGCCACTGTATCCTGTCGTTCTTTGTACGTCTTTGGTXXXXXXXXXXAGGAATTCATCTTTTTTACTCAATATATCCCACACAATTT | lmxll |
| Marker3635 | CGAGGCCACTGTATCATGTCGTTCTTTGTACGTCTTTGGTXXXXXXXXXXAGGAATTCATCTTTTTTACTCAGTATATCCCACACAATTT | lmxll |
| Marker3637 | TAGAATTGTCAAGGCCTGGAAACAGATTGATGGGTTGACAXXXXXXXXXXTAGTTTCATCTCAAAACGACACCGTCTAGGACCAGGTCGC | lmxll |
| Marker3637 | TAGAATTGTCAAGGCCTGGAAACAAATTGATGGGTTGACAXXXXXXXXXXTAGTTTCATCTCAAAACGACACCGTCTAGGACCAGGTCGC | lmxll |
| Marker3649 | TTAGGAAATAAGTGGAATCCGAATTACATAATATCTATACXXXXXXXXXXAAGTTGTTCACTTCATCAAGATAGTAGTATGGATTTTTCT | lmxll |
| Marker3649 | TTAGGAAATAAGTGGAATCCTAATTACATAATATCTATACXXXXXXXXXXAAGTTGTTCACTTCATCAAGATAGTAGTATGGATTTTTCT | lmxll |
| Marker3651 | GCCGAGCAGGCCGATTTGAAGAAGCAGAGGCATTGCTGGAXXXXXXXXXXACATCCAAATTGGGCAGAGATTCATCAGCATTTGGAGAAT | hkxhk |
| Marker3651 | GCCGAGCAGGCTGATTTGAAGAAGCAGAGGCATTGCTGGAXXXXXXXXXXACATCCAAATTGGGCAGAGATTCATCAGCATTTGGAGAAT | hkxhk |
| Marker3662 | GAAGGATTACAGGATCAAGAAACAAAGGATTCACGCTGGCXXXXXXXXXXACTTGCACGCATACCCAATAGTTATTTCATTTGTTATGTT | nnxnp |
| Marker3662 | GAAGGATTATAGGATCAAGAAACAAAGGATTCACGCTGGCXXXXXXXXXXACTTGCACGCATACCCAATAGTTATCTCATTTGTTATGTT | nnxnp |
| Marker3664 | CTAAAGGAAAACCTTGGTGCAGTATCATCAAACCCTTTGTXXXXXXXXXXCCTGAGCTCTCATTTATGGTATTGTCACTATGGTTTTGTT | lmxll |
| Marker3664 | CTAAAGGAAAACCTTGGTGCAGTATCATCAAACCCTTGGTXXXXXXXXXXCCTGAGCTCTCATTTATGGTATTGTCACTATGGTTTTGTT | lmxll |
| Marker3671 | TTTTAGAGTTCATTTAGCTTCACAAATTTAGCGTAAACCAXXXXXXXXXXCACATGTTTGGTTTGTATAGGAATGTAAAACAAAGTTTGC | hkxhk |
| Marker3671 | TTTTAGAGTTCATTTAGCTTCACAAATTTAGTGTAAACCAXXXXXXXXXXCACATGTTTGGTTTGTATAGGAATGTAAAACAAAGTTTGC | hkxhk |
| Marker3675 | GTGACAGCTCCAAGATAATGATGGAGACAAATCCTCGCAAXXXXXXXXXXTTGAACTCTCAAATTGATTTACTGTGAAATTTCTATCTTT | lmxll |
| Marker3675 | GTGACAGCTCCAAGATAATGATGGAGACAAATCCTCGCGAXXXXXXXXXXTTGAACTCTCAAATTGATTTACTGTGAAATTTCTATCTTT | lmxll |
| Marker3677 | TCAACCAACCAATAATCAATATAACCAAACAACATCCATAXXXXXXXXXXTGTGATGTCAGCGCTGACGTCAGCGAGGCAAAAAAAATAT | lmxll |
| Marker3677 | TCAACCAACCAATAATCAATATAACCAAACAACATCCATAXXXXXXXXXXTGTGATGTTAGCGCTGACGTCAGCGAGGCAAAAAATATAT | lmxll |
| Marker3682 | CATAATCGAGTAATTCACCGTAGACACACACTTCATTATCXXXXXXXXXXAGGATTTGCCACCTTAGGAATAAGAACAATATAGGTATTA | lmxll |
| Marker3682 | CATAATCGAGTCATTCACCGTAGACACACACTTCATTATCXXXXXXXXXXAGGATTTGCCATCTTAGGAATAAGAACAATATAGGTATTA | lmxll |
| Marker3689 | TCTAATTGAACTTGGGAACTTATCTGCTTGAGTTAGTTATXXXXXXXXXXGAAGAAGTTTTCAATATACTGAGAAGATACCGCATGCGGC | hkxhk |
| Marker3689 | TCTAATTGAACTTGGGAACTTATCTGCTTGAGTTAGTTATXXXXXXXXXXGAAGAAGTTTTCAGTATACTGAGAAGATACCGCATGCGGC | hkxhk |
| Marker3691 | AGAAAAAACAATAACATTCATAAACTGACAAAATGAGAATXXXXXXXXXXACCTTCCAGTCCAGAGAAAATGAAATGGGCGGTCCCTGGT | nnxnp |
| Marker3691 | AGAAAAAACAATAACATTCATAAACTGACAAAATGAGAATXXXXXXXXXXACCTTCCAGTCCAGAGAAACTGAAATGGGCGGTCCCTGGT | nnxnp |
| Marker3703 | TATCAAAGAGAGGGAGGGAATTCGACAGAAATGTATTGATXXXXXXXXXXAGCGTTCTCACTTGATCCGAGTCGTTCTTTTATAGGTTTT | lmxll |
| Marker3703 | TATCAAAGAGAGGGAGGGAATTCGACAGAAATGTACTGATXXXXXXXXXXAGCGTTCTCACTTGATCCGAGTCGTTCTTTTATAGGTTTT | lmxll |
| Marker3706 | GCACCTCAGAGTGCTAAATCTGAGTGGATATGGTACTAAAXXXXXXXXXXCATTTCAGAGCTCCTGAAACTGAATGAACTTCGAGGCAAA | efxeg |
| Marker3706 | GCACCTCAGAGTGCTAAATCTGAGTGGATATGGTACTAAAXXXXXXXXXXCATTTCAGAGCTCCTGAAAATGAATGAACTTCGAGGCAAA | efxeg |
| Marker3708 | TGAATCCCACTCGCTGAGCAAAAATTCCTCTCCTTCGCTTXXXXXXXXXXGATCAAAGCTTTCCACCTGTCCAGCCCTTCTTGGGTGGGC | efxeg |
| Marker3708 | TGAATCCCACTCGCTGAGCAAAAGTTCCTCTCCTTCGCTTXXXXXXXXXXGGTCAAAGCTTTCCACCTGTCCAGCCCTTCTTGGGTGGGC | efxeg |
| Marker3711 | AACAGGCACCAATAAAGCCAGCAAGAGAGACAAGCAACATXXXXXXXXXXATATGCTTTGGGATTTTGTGTGCAAGTAGGGTTTTTTATC | lmxll |
| Marker3711 | AACAGGCACCAATAAAGCCAGCAAGAGAGACAAGCAACATXXXXXXXXXXATATGCTTTGGGATTTTGTGTGCAAGTAGGGTTTTTGATC | lmxll |
| Marker3713 | GTCTCTCAATTTCTAAAATTGTTCCCGTGGTCCTTCCACCXXXXXXXXXXGCTTTCTTCAAAGCTCAGAAGGGTACGTGTACATTGGTTG | hkxhk |
| Marker3713 | GTCTATCAATTTCTAAAATTGTTCCCGTGGTCCTTTCACCXXXXXXXXXXGGTTTCTTCAAAGCTCAGAAGGGTACGTGTACATTGGTTG | hkxhk |
| Marker3717 | TGGGAGGTATAAATACCAAGCCCTATCTCAAATCTCACCCXXXXXXXXXXTCTGAATCAGCCACATTGATAGACCTTACTCTTGGAGCTC | lmxll |
| Marker3717 | TGGGAGGTATAAATACCAAGCCCTATCTCAAATCTCACCCXXXXXXXXXXTCTGAATCAGCCACATTGATAGACCTTACTTTTGGAGCTC | lmxll |
| Marker3720 | GACTTCTGGTCTGCAAAGTCAATGCCCTTTTTCATTACACXXXXXXXXXXGAAAATCCAATCCAAACAAAGAACATCCTACTCAAAGCCA | nnxnp |
| Marker3720 | GACTTCTGGTCTGCAAAGTCAATGCCTTTTTTCATTACACXXXXXXXXXXGAAAATCCAATCCAAACAAAGAACATCCTACTCAAAGCCA | nnxnp |
| Marker3721 | AAAGATATAAAAAAACAAAAGGGAGTGTAAGTGGATAAAAXXXXXXXXXXTTATTCTGGCTTTGCTTGTCTCACAACCCTTTTTGGAATA | lmxll |
| Marker3721 | AAAAATATAAAAAAACAAAAGGGAGTGTAAGTGGATAAAAXXXXXXXXXXTTATTCTGGCTTTGCTTGTCTCACAACCCTTTTTGGAATA | lmxll |
| Marker3723 | CAACACTCTCTCATGCGCTGCGAATTTCCTTTTGTTTGTAXXXXXXXXXXCAGAGTTCTAATGGACTTGACATGCTCAACATTTCGAGGA | hkxhk |
| Marker3723 | CAACACTTTCTCATGCGCTACGAATTTCCTTTTGTTTGTAXXXXXXXXXXCAGAGTTCTAATGGACTTGACATGCTCAACATTTCGAGGA | hkxhk |
| Marker3729 | GGTAATAAATAATTGCAAGATTATAGCTTTTGATCTGAAAXXXXXXXXXXTTGCATGTGCACGGAAAGGAAAGCTTCACTAATGCTAACT | lmxll |
| Marker3729 | GGTAATAAATAATTGCAAGATTATAGCTTTTGATCTGAAAXXXXXXXXXXTTGCATGTGCACGGAAAGGAAAGCTCCACTAATGCTAACT | lmxll |
| Marker3733 | AATTCTTCACAAAGAGGGATCTAAAGTTTCTATTGAGATGXXXXXXXXXXACCCTCATTGGAAGGGTGCGTTTACTATTTTATGGAAGAC | lmxll |
| Marker3733 | AATTCTTCACAAAGAGGGATCTAAAGTTTCTATTGAGATGXXXXXXXXXXACCCTCATTGGAAGGAAGCGTTTACTATTTTATGGAAGAC | lmxll |
| Marker3737 | AAAAAGGTTGTATCTCATTTATTGACTATCTTATTGGTCAXXXXXXXXXXTGCCAAACCTCAACTGCAAGAATATAAAAATAAAACAAAA | nnxnp |
| Marker3737 | AAAAAGGTTGTATATCTCATTTATTGACTATCTTATTGGTXXXXXXXXXXTGCCAAACCTCAACTGCAAGAATATAAAAATAAAACAAAA | nnxnp |
| Marker3738 | TAATCAGTATCTAACAGATTGAAATTCATGATACACATCAXXXXXXXXXXAGAATGGAAGTGGGAAAATCTGGTAAGACAACCAGTTTCA | hkxhk |
| Marker3738 | TAATCAGTATCTAACAGATTGAAATTCATGATACACATCAXXXXXXXXXXAGAATGGAAGTGGGAAAACCTGGTAAGACAACCAGTTTCA | hkxhk |
| Marker3739 | TAACTTGAATGGTAAAGACACTTCAGAAAATAAGTAAATGXXXXXXXXXXACAAAATCCTTGATGCACTTGCTAACACATCTGCGAATTC | lmxll |
| Marker3739 | TAACTTGAATGGTAAAGACACTTCAGAAAATAAGTAAATGXXXXXXXXXXACAAAATCCTTGATGCACTTGCTAACACGTCTGCGAATTC | lmxll |
| Marker3743 | AAACTTGTAACATGCAATTATGAGAGATGGTGAATGGTAGXXXXXXXXXXATCTGAAGTCATTGTGGGAGAAAATAAAGACCTGAAAAAG | nnxnp |
| Marker3743 | AAACTTGTAACATGCAATTATGAGAGATGGTGAATGGTAGXXXXXXXXXXATCTGAAGCCATTGTGGGAGAAAATAAAGACCTGAAAAAG | nnxnp |
| Marker3751 | CACAAAGCAAGGGCACGAGAACACCAATTTTGAGCCCATCXXXXXXXXXXGATCAGAATGCTACTGACCTGGATGATAACAGTGATGATT | lmxll |
| Marker3751 | CACAAAGCAAGGGCACGAGAACACCAATTTCGAGCCCATCXXXXXXXXXXGATCAGAATGCTACTGACCTGGATGATAACAGTGATGATT | lmxll |
| Marker3752 | GAAAATACTAAAATTTCAATAATTCAATTGAGTGGTCAAAXXXXXXXXXXTCTATAAGAATTCTTGCAGATAAAATGGTCGATCGAATGA | nnxnp |
| Marker3752 | GAAAGTACTAAAATTTCAATAATTCAATTGAGTGGTCAAAXXXXXXXXXXTCTATAAGAATTCTTGCAGATAAAATGGTTGATCGAATGA | nnxnp |
| Marker3757 | GCGACGAAAAGATTATGAGATGGAAGTATATATGCGGCACXXXXXXXXXXTGGTTTTGCAGCTTTTATATTTATATTTTTCTAATTTTGG | nnxnp |
| Marker3757 | GCGACGAAAAGATTATGAGATGGAAGTATATATGCCGCACXXXXXXXXXXTGGTTTTGCAGCTTTTATATTTATATTTTTCTAATTTTGG | nnxnp |
| Marker3760 | GAGTTGGTTTGCATAAGTCATACCAGTTGAGCCAGCACAAXXXXXXXXXXAAGATATGTGCCAATGACAATGCCAGTTGCAAATATCTCT | lmxll |
| Marker3760 | GATTTGGTTTGCATAAGTCATACCAGTTGAGCCAGCACAAXXXXXXXXXXAAGATATGTGCCAATGACAATGCCAGTTGCAAATATCTCT | lmxll |
| Marker3765 | AGGAAAAAAAAAAGAAAAAAAAGAAAAAGAAAAAAAAGAAXXXXXXXXXXTGGAATCCCACATCGGTGGGGTGAAAGAAAACAAATGGGT | nnxnp |
| Marker3765 | AGGAAAAAAAAAAGAAAAAAAAGAAAAAGAAAAAAAAAGAXXXXXXXXXXTGGAATCCCACATCGGTGGGGTGAAAGAAAACAAATGGGT | nnxnp |
| Marker3768 | ATTAGCTATTTGCATTGTTTCCACAGACTTGACTCGTAGAXXXXXXXXXXCGTAAAAAGTTATAACCATAGTTATGCTTATGAACACGTA | nnxnp |
| Marker3768 | ATTAGCTATTTGCATTGTTTCCACAGACTTGACTCGTAGAXXXXXXXXXXCGTAAAAAGTTATAACCATAGTTATGCTTATGAACACGTG | nnxnp |
| Marker3770 | TTTATTGAAGAAAAAAAATCACATAAATATACATATAGAGXXXXXXXXXXTTTGCTTTACAGAGATCACCCGCACACAAAAGCTTGAGCA | efxeg |
| Marker3770 | CTTATTGAAAAGAAAAAAAATCACATAAATATACATATAGXXXXXXXXXXTTTGCTTTACAGAGATCACCCGCACACAAAAGCTTGAGCA | efxeg |
| Marker3772 | CACAAAGCAAGTGCACGAGAATACCAATTTCGAGCCCATCXXXXXXXXXXCATGGAGGCAACGCCGATCAGAATGCTGCTGACCTTGATT | lmxll |
| Marker3772 | CACAAAACAAGTGCACGAGAATACCAATTTCGAGCCCATCXXXXXXXXXXCATGGAGGCAACGCCGATCAGAATGCTGCTGACCTTGATT | lmxll |
| Marker3779 | GAATCTGACAGATGGAGGGGCTGATTACTCATTTGAATGTXXXXXXXXXXCTTCATTGAAGCTTCAATAAGTCCAATCTCCCTTCTAATA | lmxll |
| Marker3779 | GAATCTGACAGATGGAGGGGCTGATTACTCATTTGAATGTXXXXXXXXXXCTTCATTGAAGCTTCAATGAGTCCAATCTCCCTTCTAATA | lmxll |
| Marker3780 | GGCGAGCGATCGCCATTCGATCGTGCGATATAGAATTATCXXXXXXXXXXTTGTTTGAGTTGCTCGTTGAGGTTTGGGATTGAGGATTGG | lmxll |
| Marker3780 | GGCGAGCGATCGTCATTCGATCGTGCGATATAGAATTATCXXXXXXXXXXTTGTTTGAGTTGCTCGTTGAGGTTTGGGATTGAGGATTGG | lmxll |
| Marker3784 | TTTGAACAGAGGGAGTTTGCTATGCAAAGAAGGACTATAAXXXXXXXXXXTTGAATAACATATGGTAGCGACTTGTAACTGTGTTCTCAT | lmxll |
| Marker3784 | TTTGAACAGAGGGAGTTTGCTATGCAAAGAAGGACTATAAXXXXXXXXXXTTGAATACCATATGGTAGCGACTTGTAACTGTGTTCTCAT | lmxll |
| Marker3786 | AACTTTGAATCGTTTTAGGTAGCTTCTCTTAGTCTTGAGAXXXXXXXXXXATCGACATGAGGTATGTCTTGACATCTTGCTTTTCAATGC | lmxll |
| Marker3786 | AACTTTGAACCGTTTTAGGTAGCTTCTCTTAGTCTTGAGAXXXXXXXXXXATCGACATGAGGTATGTCTTGACATCTTGCTTTTCAATGC | lmxll |
| Marker3797 | GTGACAAGGTACAAGCAAAAATCTATGAATACTCAACAAAXXXXXXXXXXTTCGGCAAATTCCTCTGGGAATTTCTTTTGTATAGTGTCA | nnxnp |
| Marker3797 | GTGACAAGGTACAAGCAAAAATCTATGAATACTCAACAAAXXXXXXXXXXTTCGGCAAATTCCTCTGGGAATTTCTTTTGTGTAGTGTCA | nnxnp |
| Marker3813 | AGGCACGTGAGCGACGTGCGTGTTTGGGTTTGATTAGGCAXXXXXXXXXXAACATCAGTTCACCAAAACAGCATTTGAGGTATATTAGAA | lmxll |
| Marker3813 | AGGCACGTGAGCGACGTGCGTGTTTGGGTTTTGATTAGGCXXXXXXXXXXAACATCAGTTCACCAAAACACCATTTGAGGTATATTAGAA | lmxll |
| Marker3818 | AACATTACCATAAAATCAACATCCTACCCAACCATGTTATXXXXXXXXXXCTGCCCAGGGAGCACATCAAGAAATGGGTGTGAGAGAGAG | nnxnp |
| Marker3818 | AAATTACCATAAAATCAACATCCTACCCAACCATGTTATAXXXXXXXXXXCTGCCCAGGGAGCACATCAAGAAATGGGTGTGAGAGAGAG | nnxnp |
| Marker3819 | CACTCCCCCTCAAGTTGGAGAGTGAATGTCAAGAACTTCCXXXXXXXXXXGAAAGAGAGACGATCTTTTGTCTCTTTGTTCTCCAAGAAA | lmxll |
| Marker3819 | CACTCTCCCTCAAGTTGGAGAGTGAATGTCAAGAACTTCCXXXXXXXXXXGAAAGAGAGACGATCTTTTGTCTCTTTGTTCTCCAAGAAA | lmxll |
| Marker3823 | GCTTGTGAAACTGTACTTTTGCTTGTGTGATTTGTGTGTAXXXXXXXXXXCTGCTGCATATCTTTGGTCAGTTTTCAATCATTATCATTT | nnxnp |
| Marker3823 | GCTTGTGAAACTGTACTTTTGCTTGTGTGATTTGTGTGTAXXXXXXXXXXTGCTGCATATCTTTGGTCAGTTTTCAATCATTATCATTTT | nnxnp |
| Marker3824 | GTTGGGGACATATTGGCATTACTAGTAGTGGTATCATTCAXXXXXXXXXXTTGAAATAGAAATGGAAAGGAATAATATTGGTTTTTATGT | efxeg |
| Marker3824 | GTTGGGGACATATTGGCGTTACTAGTAGTGGTATCATTCAXXXXXXXXXXTTGAAATAGAAATGGAAAGGAACAATATTGGTTTTTATGT | efxeg |
| Marker3825 | AGTTGGGATTTTGGAAGTGATGGATTTGCTCTCATCTTATXXXXXXXXXXGATTCAATCTGTTCCTTGCTAATGGCAAAAGTACCTCTTC | nnxnp |
| Marker3825 | AGTTGGGATTTTGGAAGTGATGGATTTGCTCTCATACTTAXXXXXXXXXXGATTCAATCTGTTCCTTGCTAATGGCAAAAGTACCTCTTC | nnxnp |
| Marker3832 | CGACATTGAATATGCTGTCGTTTGGTTGGGGGCCTTGTGGXXXXXXXXXXATAGTAATGTTGATTTGCTGATGGAGTTTTTTTTTCATTT | nnxnp |
| Marker3832 | CGACATTGAATATGCTGTCGTTTGGTTGGGGGCCTTGTGGXXXXXXXXXXTAGTAATGTTGATTTGCTGATGGAGTTTTTTTTTTCATTT | nnxnp |
| Marker3834 | GTAAACAATTTAGAGTTCTTCCTCAATAGAAAAAAAATCTXXXXXXXXXXTCTCTGTCAATTTACCTATTTGTTCTCCGTTTGGATTTTG | lmxll |
| Marker3834 | GTAAACAATTTAGAGTTCTTCCTCAATAGAAAAAAAAATCXXXXXXXXXXTCTCTGTCAATTTACCTATTTGTTCTCCGTTTGGATTTTG | lmxll |
| Marker3837 | TGGAGCAGCAAGTATTATATTATCAATATCTTTCCTGCATXXXXXXXXXXAATAGTGATATGCTGTTACAAACATGCACTTTGGGGACAG | nnxnp |
| Marker3837 | TGGAGCAGCAAGTATTATATTATCAATATCCTTCCTGCATXXXXXXXXXXAATAGTGATATGCTGTTACAAACATGCACTTTGGGGACAG | nnxnp |
| Marker3844 | AGTTGCAGATTTCCTCCTCTTCCCCCCTTCAGCCTCCAAAXXXXXXXXXXGTATGTTGAGATGACACCTTTGAATTTTTCCAGGAATTAT | lmxll |
| Marker3844 | AGTTGCAGATTTCCTCCTCTTCCCCCCTTCAGCCTCCAAAXXXXXXXXXXGTATGTTGAGATGGCACCTTTGAATTTTTCCAGGAATTAT | lmxll |
| Marker3846 | TTCTCAAACCATTAGTCACCGAAATCCTCATCCGATCAACXXXXXXXXXXGAGGAATGACTTAGTCATTAGATCATTCACCTTTGAGGCA | lmxll |
| Marker3846 | TTCTCAAACCATTAGTCACCGAAATCCTCATCCGATCAACXXXXXXXXXXGAGGAATGACTTAGTCATTAGATCATTCATCTTTGAGGCA | lmxll |
| Marker3848 | TGATTTCCAATTTACTTTTCAACTAAAGCTCAAGATTCAAXXXXXXXXXXGGCAAAAGTGAAGCTCAATTTGCCAATCAGAACCATGGCC | lmxll |
| Marker3848 | TGATTTCCAATTTACTTTTCAACTAAAGCTCAAGATTCAAXXXXXXXXXXGGCAAATGTGAAGCTCAATTTGCCAATCAGAACCATGGCC | lmxll |
| Marker3856 | TTCAATGAGTTGACAGTTTGAATCAAACGAACAAATTCATXXXXXXXXXXTATACTTCTGCATTTGAATGTTGAGGTTATGGGTTGTAAG | lmxll |
| Marker3856 | TTCAATGAGTTGACAGTCTGAATCAAACGAACAAATTCATXXXXXXXXXXTATACTTCTGCATTTGAATGTTGAGGTTATGGGTTGTAAG | lmxll |
| Marker3858 | TGGGAATTCCAACTCTCAGAAGGCTTAGAACATTTGAGAGXXXXXXXXXXCCACCTTTTGAAAATAAGTTATCAATTGTCATGTTATGTG | lmxll |
| Marker3858 | TGGGAATTCCAACTCTCAGAAGGCTTAGAACATTTGAGAGXXXXXXXXXXCTACCTTTTGAAAATAAGTTATCAATTGTCATGTTATGTG | lmxll |
| Marker3870 | GAACGATTTAGTCATCAAATCATTCATCTTTGCAAAGAATXXXXXXXXXXTTCGCAATTTCAAGGTTTGAGGTCTATCAGAGAACAAGAA | lmxll |
| Marker3870 | GAACGATTTAGTCATCAAATCATTCATCTTTGCAAAGAATXXXXXXXXXXTTCGCAATTTCAAGGTTTGAGGCCTATCAGAGAACAAGAA | lmxll |
| Marker3878 | CATGTTGATGGTTTCACGTTTCTCATGTTTATGCCTAATAXXXXXXXXXXAATGATGCATATCATGGTGGCGAATATCATAAGTTCGCTT | hkxhk |
| Marker3878 | CATGTTGATGGTTTCACGTTTCTCATGTTTATGCCTAATAXXXXXXXXXXAATGATGCAGATCATGGTGGCGAATATCATAAGTTCGCTT | hkxhk |
| Marker3879 | GCTAATGAATAAAGAAGAGGAAAAAACAGACATCTTTCACXXXXXXXXXXTGGGGTGTTAGTTGGATTGGCACAAGTCTTTGGTGTACTC | hkxhk |
| Marker3879 | GCTAATGAATAAAGAAGAGGAAAAAACAGACATCTTTCACXXXXXXXXXXTGGGGTGTTAGTTGGATTGGCACAAGTCTTTGGTGTGCTC | hkxhk |
| Marker3885 | GAAAGCCATGCCTGGAGGTTCCCCTAAAATGATTATTACTXXXXXXXXXXCCATGCATTCTTCAAGAGATATGTTTCCAAAAATAATTCT | nnxnp |
| Marker3885 | GAAAGCCATGCCTGGAGGTTCCCCAAAAATGATTATTACTXXXXXXXXXXCCATGCATTCTTCAAGAGATATGTTTCCAAAAATAATTCT | nnxnp |
| Marker3887 | AATGTTTTTCATGGGTCTTTTCAGGCTTATATATATATTCXXXXXXXXXXTGTTCATCTTCTCTGCTCCGGAAGATTTATGCTCTATGAT | lmxll |
| Marker3887 | AATGTTTTTCATGGATCTTTTCAGGCTTATATATATATTCXXXXXXXXXXTGTTCATCTTCTCTGCTCCGGAAGATTTATGCTCTATGAT | lmxll |
| Marker3889 | CAACAAAAGATTACTAACTGCTTCGATTTTTGCAAACTTTXXXXXXXXXXCTGTGAGACTCACACTTCTTCTCTCACTCGGACTCTGGCA | nnxnp |
| Marker3889 | CAACAAAAGATTACTAACTGCTACGATTTTTGCAAACTTTXXXXXXXXXXCTGTGAGACTCACACTTCTTCTCTCACTCGGACTCTGGCA | nnxnp |
| Marker3892 | GAGAATTTACTCATGCACCTAAGGTCTTGTGTTCGAATAAXXXXXXXXXXGTTAGCCACAATTTTGACCCCAAAGAGAGAAAAATAAAGA | nnxnp |
| Marker3892 | GAGAATTTACTCATGCACCTGAGGTCTTGTGTTCGAATAAXXXXXXXXXXGTTAGCCACAATTTTGACCCCAAAGAGAGAAAAATAAAGA | nnxnp |
| Marker3893 | AAGGAAACCTTCTAATCCTTGGAACCAATGATGCCCTCCAXXXXXXXXXXCATTTCCCATTGCCGAAAGCATTAGAGGATAGGTGAAGAG | lmxll |
| Marker3893 | AAGGAAACCTTCTAATCCTTGGAACCAATGATGCCCTCCAXXXXXXXXXXCATTTCCCATTGCTGAAAGCATTAGAGGATAGGTGAAGAG | lmxll |
| Marker3900 | TAAATGGCATGCCGCCACCACCACCTTTGTCTGGTACAGCXXXXXXXXXXTTTAGACAATTATGACATACCAAGATGGAAACTACTTCTT | hkxhk |
| Marker3900 | TAAATGGCATGCCGCCACCACCACCTTTGTCTGGTACAGCXXXXXXXXXXTTTAGACAATTATGACATACCCAGATGGAAACTACTTCTT | hkxhk |
| Marker3902 | TGAGTAACACGTACCTCCAAGATACTTGCTCTTCTCGATGXXXXXXXXXXTACATTCAAAAATTCGTTTTTCAATGTCTGTAAAGCTAAT | lmxll |
| Marker3902 | TGAGTAACACGTACCTCCAAGATACTTGCTCTTCTCGATGXXXXXXXXXXTACATTCAGAAATTCGTTTTTCAATGTCTGTAAAGCTAAT | lmxll |
| Marker3903 | TGATCACTGAGAAGTTGCTTTAGGAAGCAATTGTAAGTAGXXXXXXXXXXCAATAGAGAAAGATACAAGTAGATCTGAACATGTTCTGTC | lmxll |
| Marker3903 | TGATCACTGAGAATTTGCTTTAGGAAGCAATTGTAAGTAGXXXXXXXXXXCAATAGAGAAAGATACAAGTAGATCTGAACATGTTCTGTC | lmxll |
| Marker3906 | TGCAAAATCAAGACTTCAATTACAATGTTTTCTTGCATACXXXXXXXXXXATAAGTGGCATTCAAGAAATATCAAAGTTATAAGTTCTTG | lmxll |
| Marker3906 | TGCAAAATCAAGACTTCAATTACAATGTTTGCTTGCATACXXXXXXXXXXATAAGTGGCATTCAAGAAATATCAAAGTTATAAGTTCTTG | lmxll |
| Marker3909 | TTGGAAATAAATAATTAGAAGAATAATGAGAGAGAGAGAGXXXXXXXXXXAAATCTTCCATTCTCATCTCTTGAGAATATACATTATCGT | lmxll |
| Marker3909 | TTGGAAATAAATAATTAGAAGAATCATGAGAGAGAGAGAGXXXXXXXXXXAAATCTTCCATTCTCATCCCTTGAGAATATACATTATCGT | lmxll |
| Marker3915 | AACAAGATTATATTTTAGTAAGTAATACCAGGGGATGTATXXXXXXXXXXGGCCCTGTCTCACTTGACTTGCCAAATTTTTGACAACAAA | lmxll |
| Marker3915 | AACAAGATTATATTTTAGTAAGTAATACCAGGGGATGTATXXXXXXXXXXGGCCCTGTCTCACTTGACTTGCCAAATATTTGACAACAAA | lmxll |
| Marker3917 | AAATGTAATTGTTTCGCCACTTCCAAATATACCAACAAGTXXXXXXXXXXGTCATCACCATGGTCTCTGAAGAGCAGATTATAGGCTGAC | lmxll |
| Marker3917 | AAATGTAATTGTTTCGCCACTTCCAAATATACCAACAAGTXXXXXXXXXXGTCATCACCATGGTCTCTGAAGAGCAGATTATAGGCTGAT | lmxll |
| Marker3920 | ATTGTTCTACTTCAGCTCTTCAACACACACACACACACACXXXXXXXXXXTATAATAAATACATAATGCAGGCATTTCCAATTCATTTGG | lmxll |
| Marker3920 | ATTGTTCTACTTCAGCTCTTCAACACACACACACACACACXXXXXXXXXXTATAATAAATACATAATGCAGGCATTTCCAATTCGTTTGG | lmxll |
| Marker3932 | TCACCAAATCATTTTTCAGCAAGTTTCACACTAAAAAAATXXXXXXXXXXCTTCTTTTCTTTTTTCTTTTTTCTTCTTTTTTTTTAGCAA | nnxnp |
| Marker3932 | TCACCAAATCATTTTTCAGCAAGTTTCACACTAAAAAAATXXXXXXXXXXTCTTCTTTTCTTTTTTCTTTTTTCTTCTTTTTTTCAGCAA | nnxnp |
| Marker3943 | GAAAGCATGCGAAAATTTTCGGGCCCTTTATGATTTGGTCXXXXXXXXXXAAAGGTTCCATTGAATAGTCATTACATTGAAACAACAAAT | lmxll |
| Marker3943 | GAAAGCATGCGAAAATTTTCGGGCCCTTTATGATTTGGTCXXXXXXXXXXAAAGGTTCCATTGAATAGTCGTTACATTGAAACAACAAAT | lmxll |
| Marker3949 | AGCAAGAAATAAAAGAAGTTGAATGGTCCAACTTCACTATXXXXXXXXXXGGGAGTCTTTCACGGGCATAGAATATTTCTTTGACAAATA | nnxnp |
| Marker3949 | AGCAAGAAATAAAAGGAGTTGAATGGTCCAACTTCACTATXXXXXXXXXXAGGGAGTCTTTCACGGGCATAGAATATTTCTTGACAAATA | nnxnp |
| Marker3954 | ACACCCACAACTTGCTTCTTGGAATGTAATAGACCCGTAAXXXXXXXXXXGAAGGCCCCCAAACCTGGTCCAAAACATCAGAATTGTTAG | lmxll |
| Marker3954 | ACACCCACAACTTGCTTCTTGGAATGTCATAGACCCGTAAXXXXXXXXXXGGAGGCCCCCAAACCTGGTCCAAAACATCAGAATTGTTAG | lmxll |
| Marker3961 | TCACATCTTTGATCTCAAATTCTCAATCCCCATGGCAAACXXXXXXXXXXAAAAGGGTGCAATTTCAGTATGATCCGCAAAGCTATGCTC | hkxhk |
| Marker3961 | TCACATCTTTGATCTCAAATTCTCAATCCCCATGGCAAACXXXXXXXXXXAGAAGGGTGCAATTTCAGTATGATCCGCAAAGCTATGCGC | hkxhk |
| Marker3967 | GGGCCTTTCAGTCATTTACACGGAGAAGGCGGAGCCAATCXXXXXXXXXXCTGCTCGCCGAGTCTTGCTCTCTCTCTATATTGATTGCGA | lmxll |
| Marker3967 | GGGCCTTTCAGTCATTTACACGGAGAAGGCGGAGCCAATCXXXXXXXXXXCTGCTCGCCGAGTCGTGCTCTCTCTCTATATTGATTGCGA | lmxll |
| Marker3970 | CCCAATATTCAGCAGGATTGTTGATCTCCATTGCATACTCXXXXXXXXXXATCATCCGGCTCTTGTACTTTAGTGCTTTTTATTTGTTTA | lmxll |
| Marker3970 | CCCAATATTCAGCAGGATTGTTGATCTCCATTGCATACTCXXXXXXXXXXATCATCCGGCTCTTGAACTTTAGTGCTTTTTATTTGTTTA | lmxll |
| Marker3975 | ACTCTAAAGCCCTTTTCTCTGCATCAAAGTAGAGAGAGGAXXXXXXXXXXAAATAGCCCATTCACTGTTTTTATTTTATACTCAAATATA | nnxnp |
| Marker3975 | ACTCTAAAGCCCTTTTCTTTGCATCAAAGTAGAGAGAGGAXXXXXXXXXXAAATAGCCCATTCACTGTTTTTATTTTATACTCAAATATA | nnxnp |
| Marker3977 | CATGAAAGAGTCCGGTGATTGTAAATGACACATGATCTCCXXXXXXXXXXATAGTTCAATAACTTACAACCAAAATGAAAGTTGTCAGTA | nnxnp |
| Marker3977 | CATGAAAGAGTCAGGTGATTGTAAATGACACATGATCTCCXXXXXXXXXXATAGTTCAATAACTTACAACCAAAATGAAAGTTGTCAGTA | nnxnp |
| Marker3980 | TATAAGTGTGTAATTTTTATTAGTTTGATCTAAACTGTCAXXXXXXXXXXAACTTTAGTCATGTGACGTATCAATGCTTCAAAAATGTGT | nnxnp |
| Marker3980 | TATAAGTGTGTAATTTTTATTAGTTTGATCTAAACTGTCAXXXXXXXXXXAACTTTAGTCATGTGACGTATCAATGCTTCAAAAATGTGC | nnxnp |
| Marker3986 | AGAGACAAATGGGTGTAATTTATGCGGTAAAAAGCCTCTGXXXXXXXXXXCAATACTCACCGTTTTAGTTGCATGTTAGGTGGCCAATGT | lmxll |
| Marker3986 | AGAGACAAATGGGTGTAATTTATGCGGTGAAAAGCCTCTGXXXXXXXXXXCAATACTCACCGTTTTAGTTGCATGTTAGGTGGCCAATGT | lmxll |
| Marker3992 | CTGCTTTGCTCCAAACTTGTCCATCCAGGGATGTCTCGTTXXXXXXXXXXCATCTTTGTTGTCTCGACCTCTGCCTTCATCTCTGTTAGC | lmxll |
| Marker3992 | CTCCTTTGCTCCAAACTTGTCCATCCAGGGATGTCTCGTTXXXXXXXXXXCATCTTTGTTGTCTCGACCTCTGCCCTCATCTCTGTTACC | lmxll |
| Marker4007 | ACAACATTGGCAGCGAAATCTTCAGCATTTCTTCGCGAACXXXXXXXXXXCTAAAGGTTGTTAGGTTGAAGAAATAAGTGTCTGACTTGA | hkxhk |
| Marker4007 | ACAACATTGGCAGCGAAATCTTCAGCATTTCTTCGCGAACXXXXXXXXXXCTAAAGGTTGGTAGGTTGAAGAAATAAGTGTCTGACTTGA | hkxhk |
| Marker4023 | ATCTTTTGAGCAACAGAAGGGTTCGGTCTTTTCGTTCTGTXXXXXXXXXXCAAACGACTTTGTTTGGTCCAAGGCTCAGTCAAATTTTTT | lmxll |
| Marker4023 | ATCTTTTGAGCAACAGAAGGGTTCGGTCTTTTCGTTCTGTXXXXXXXXXXCAAACGACGTCGTTTGGTCCAAGGCTCAGTCAAATTTTTT | lmxll |
| Marker4024 | AATAATTACAAATATACGGGGCCTCGTGTTTTTGTTTCCGXXXXXXXXXXCCCAAAAGTCATATTATGTCAATTGCATAAGTAATCACCC | nnxnp |
| Marker4024 | AATAATTACAAATATACGGGGCCTCGTGTTTTTGTTTCCGXXXXXXXXXXCCCAAAAGTCACACTATGTCAATTGCATAAGTAATCACCC | nnxnp |
| Marker4026 | GGTGTCTATCATAGTTGTGTAGGTCCAAGCGAATGTACAAXXXXXXXXXXCTTACGGTGTCATAATTAGACTTCATGGACTCCACCTTTG | lmxll |
| Marker4026 | GGTGTCTATCATAGTTGTGTAGGTCCAAGCGAATGTACAAXXXXXXXXXXCTTACGGTGTCATAATCAGACTTCATGGACTCCACCTTTG | lmxll |
| Marker4029 | GTCGTCAGTCTTTTGTGTGTTTGTGTGTGTGTGTGTGTGTXXXXXXXXXXCCAATGTATAAATGGTCCAGCTAAATCGCAATCACGACAA | hkxhk |
| Marker4029 | GTCGTCAGTCTTTTTGTGTGTTTGTGTGTGTGTGTGTGTGXXXXXXXXXXCCAATGTATAAATGGTCCAGCTAAATCGCAATCACGACAA | hkxhk |
| Marker4040 | TCCCAGAAAAGTAGCTTTGATTTTGATTGAGCACTATTTCXXXXXXXXXXCATCAGTCATTACGTATCTCCAATGTATATATATACTGTA | lmxll |
| Marker4040 | TCCCATAAAAGTAGCTTTGATTTTGATTGAGCACTATTTCXXXXXXXXXXCATCAGTCATTACGTATCTCCAATGTATATATATACTGTA | lmxll |
| Marker4043 | ACTCCATCACAAGGATTCATATGGAAGGCTGCACCAATCTXXXXXXXXXXTTTTGTGGTGCCTCAAAGTGTTGGTCGTAATTTGAAAGGG | lmxll |
| Marker4043 | ACTCCATCACAAGGATTCATATGGAAGGCTGCACCAATCTXXXXXXXXXXTTTCGTGGTGCCTCAAAGTGTTGGTCGTAATTTGAAAGGG | lmxll |
| Marker4048 | TATTTGCCATTACCTCCGTTGTGGGTAAAACATTCCAAAAXXXXXXXXXXGCTACTGGGCCCAAACTGACCAGTTCCTTTTTGGTATTGG | lmxll |
| Marker4048 | TATTTGCCATTACCTCCGTTGTGGGTAAAACATTCCAAAAXXXXXXXXXXTGCTACTGGGCCAAACTGACCAGTTCCTTTTTGGTATTGG | lmxll |
| Marker4051 | CTTACTGCTTTTTGACTGATCAAGTGCAAGTCCTGTAACCXXXXXXXXXXCTGCCACTTCAGAGACTCATTAGGAATCCCAGTCCAAACT | lmxll |
| Marker4051 | CTTACTGCTTTTTGACTGATCAAGTGCAAGTCCTGTAACCXXXXXXXXXXCTGCCACTTCAGAGACTCATTAGGAATCCCAGTCCAATCT | lmxll |
| Marker4052 | AAGAAAGATCAGGATGCAAGAACAGACTAAATGGGGCTGCXXXXXXXXXXAGTAAAAGAGTATAACTTGTGGGGTTAGGAAAGCCTTAGC | hkxhk |
| Marker4052 | AAGAAAGATCAGGATGCAAGAACAGACTAAATGGGGCTGCXXXXXXXXXXAGTAAAAGAGTATAACTTGTGGGGTTAGCAAAGCCTTAGC | hkxhk |
| Marker4075 | GAACTGATTTTTGGAAAGTTGAGAAAAGATTTGGAAAATGXXXXXXXXXXTTGACGGAATATTATTGGAATTGACAGAATAGTAATGGAA | lmxll |
| Marker4075 | GAACTGATTTTTGGAAAGTTGAGAAAAGATTTGGAAAATGXXXXXXXXXXTTGACGGAATATTATTGGAATTGACGGAATAGTAATGGAA | lmxll |
| Marker4078 | AGAATAAGAGAAAAATTATGTGTTTAGGGTTTTGTGATAAXXXXXXXXXXCAAAGATTTGTCAACTAAATAGAATGGGAAAGTAAATCTC | lmxll |
| Marker4078 | AGAATAAGAGAAAAATTATGTGTTTAGGGTTTTGTGATAAXXXXXXXXXXCAAAGATTTGTCAACTAAATAAAATGGGAAAGTAAATCTC | lmxll |
| Marker4083 | ACATTGGCGAATGCAACGCACGCGTTGTTGGTAAAAAAAAXXXXXXXXXXGCATGAACTATGCTCAGAAATTGTATTGGCTTGAATTGGC | hkxhk |
| Marker4083 | ACATTTGCGAATGCAACGCACGCGTTGTTGGTAAAAAAAAXXXXXXXXXXGCATGAACTATGCTCAGAAATTGTATTGGCTTGAATTGGC | hkxhk |
| Marker4084 | AACTACAACACGGTTGCCAAAAAAAAAAAAAAAAAAAAAAXXXXXXXXXXGACTTTTGTTTCTTGAAAGCACTGAAAGGCAGAAATGCAA | lmxll |
| Marker4084 | AACTACAACACGGTTGCCAAAAAAAAAAAAAAAAAAAAAAXXXXXXXXXXGACTTTTGTTTCTTGAAAGCACTGAAAGCCAGAAATGCAA | lmxll |
| Marker4087 | CATTACCAAGCAGAAGGACTAAAGGTAAGCTCTTACCTTTXXXXXXXXXXTTCTTGCATTTCGCGATTTTCCTACAAGGAGAATGGAGTA | hkxhk |
| Marker4087 | CATTACCAAGCAGAAGGACTAAAGGTAAGCTCTTACCTTTXXXXXXXXXXTTCTTGCATTTCGCGATTTTCCTACAACGAGAATGGAGTA | hkxhk |
| Marker4088 | GGGGTTGTCGTTTGGTTTATAATACATTGAGCATGGAGAAXXXXXXXXXXTAGGAATTTCCAAAACATTTTTCCAAAGTTCATTTGAATA | nnxnp |
| Marker4088 | GGGGTTGTCGTTTGGTGAATAATACATTGAGCATGGAGAAXXXXXXXXXXTAGGAATTTCCAAAACATTTTTCCAAAGTTCATTTGAATA | nnxnp |
| Marker4100 | CTTTGGCCTTTTCAATATTCCGCCAATTTCGTATTGAAATXXXXXXXXXXGTTTGCGCAGTATACATAATGAACTAATATATAATTTAGC | lmxll |
| Marker4100 | CTTTGGCCTTTTCAATATTCTGCCAATTTCGTATTGAAATXXXXXXXXXXGTTTGCGCAGTATACATAATGAACTAATATATAATTTAGC | lmxll |
| Marker4105 | CATAAAAGCACCTGCTCTAAGTCCGAATACAGATGGAATTXXXXXXXXXXGGAATTCGCAATTCTCGAGCATGTGTTTCGAACATTACAG | lmxll |
| Marker4105 | CATAAAAGCACCTGCTCTAAGTCCCAATACAGATGGAATTXXXXXXXXXXGGAATTCGCAATTCTCGAGCATGTGTTTCGAACATTACAG | lmxll |
| Marker4107 | ATTCAAGATTGAACATCCTATAATTGAGTATGTTGTTTCAXXXXXXXXXXTTTGGATGTTGACCTCATTGCCTGTAGATAGCTTATTTGG | nnxnp |
| Marker4107 | ATTCAAGATTGAACATCCTATAATTGAGTATGTTGTTTCAXXXXXXXXXXATTGGATGTTGATCTCATTGCCTGTAGATAGCTTATTTGG | nnxnp |
| Marker4108 | CTCATGAGATGATCCACAAAAGGCATGCATTAGCCCAAGAXXXXXXXXXXAAAAGCAGGCTCAAACAAGACCGAAATGTCGAATCCCTAA | lmxll |
| Marker4108 | CTCATGAGATGATCCACAAAAGGCATGCATTAGCCCAGGAXXXXXXXXXXAAAAGCAGGCTCAAACGAGACCGAAATGTCGAATCCCTAA | lmxll |
| Marker4113 | GTTGGAGATGATATCTGGTCACATAAGCCCCTTATAATTGXXXXXXXXXXTATGCACAGACATACAAGTGAACAACAGTGACACTTTCAT | lmxll |
| Marker4113 | GTTGGAGATGATATCTGATCACATAAGCCCCTTATAATTGXXXXXXXXXXTATACACAGACATACAAGTGAACAACAGTGACACTTTCAT | lmxll |
| Marker4128 | TGAGAGCAAGTATCTGCGCTTTGCTTGTACCATAATCAACXXXXXXXXXXTTACTATGCCAAAATTACCGATTTCCCCATTCTTGCTGAC | lmxll |
| Marker4128 | TGAGAGCAAGTATCTGCGCTTTGCTTGTACCATAATCAACXXXXXXXXXXTTACTATGCCAAAATTACCGATTTCCCCATTCTTGTTGAC | lmxll |
| Marker4132 | GAAAGAGAAAATCGCATATAGTCTCCTCAACATTGAACAAXXXXXXXXXXATTCTCTCCTTCCACAGCAGAAGGCATGTTATCAGGTACG | lmxll |
| Marker4132 | GAAAGAGAAAACCGCATATAGTCTCCTCAACATTGAACAAXXXXXXXXXXATTCTCTCCTTCCACAGCAGAAGGCATGTTATCAGGTACG | lmxll |
| Marker4135 | ACGGAAATAACAATATTTTGCTATTTTCGATCAATCATTGXXXXXXXXXXGCTCGTGAGCTAAAGGCAAGTGCAGTAATAATAATATTTA | lmxll |
| Marker4135 | ACGGAAATAACAATATTTTGCTATTTTCGTTCAATCATTGXXXXXXXXXXGCTCGTGAGCTAAAGGCAAGTGCAGTAATAATAATATTTA | lmxll |
| Marker4136 | AAAATTCAAAATGGCTTTTTCCCCTGCTACCTCATTGCACXXXXXXXXXXATTATGGCGGCGGCAGTTGGAGCGGGAGCAATACCACGGA | hkxhk |
| Marker4136 | AAAATTCAAAATTGCTTTTTCCCCTGCTACCTCATTGCACXXXXXXXXXXATTATGGCGGCGGCAGTTGGAGCGGGAGCAATACCACGGA | hkxhk |
| Marker4137 | AAAATAAATCTCAACCTATAAAAAGAACCAAAACCTCATAXXXXXXXXXXCTTCTGTTCTCAGAGACCAAACAGAAGCAGAAGCAGAAGC | hkxhk |
| Marker4137 | AAAATAAATCTCAACCTATAAAAAGAACTAAAACCTCATAXXXXXXXXXXCTTCTGTTCTCAGAGACCAAACAGAAGCAGAAGCAGAAGC | hkxhk |
| Marker4142 | AAAACCATTTAGCCTCTCTCTCTCTCTCTCTCTCTCTCTCXXXXXXXXXXCTCAAATTCTCTTCCCGCTTCTCAACTTCAGATATTGAAA | lmxll |
| Marker4142 | AAAAACCATTTAGCCTCTCTCTCTCTCTCTCTCTCTCTTCXXXXXXXXXXCTCAAATTCTCTTCCCACTTCTCAACTTCAGATATTGAAA | lmxll |
| Marker4143 | TAGGTCAAACTACATAAATATTTTCTCTGGCATTATAAAAXXXXXXXXXXTGCGAGTCCATTATATATATCTTGTTGTGTTTATAGTAAA | lmxll |
| Marker4143 | TAGGTCCAACTACATAAATATTTTCTCTGGCATTATAAAAXXXXXXXXXXTGCGAGTCCATTATATATATCTTGTTGTGTTTATAGTAAA | lmxll |
| Marker4175 | CAATATCGTCACAGATGTTGTCTTCATCAGTACTATCAATXXXXXXXXXXTCTTCTGGTACCGGAACCAGTGCAACTGAGCCTTCACCAT | hkxhk |
| Marker4175 | CAATATCGTCAGAGATGTTGTCTTCATCAGTACTATCAATXXXXXXXXXXTCTTCTGGTACCGGAACCAGTGCAACTGAGCCTTCACCAT | hkxhk |
| Marker4185 | AAAGGCTAACCTCCTCGATCGACAAGAAGAAAGGTTGGTAXXXXXXXXXXTTACAAGGAACAGGTTTTCCCCAACAAAATCTCGTCTTTC | lmxll |
| Marker4185 | AAAGGCTAACCTCCTCGATCGACAAGAAGAAAGGTTGGTAXXXXXXXXXXTTACAAGGAACAGGTTTTCCCCAACAAAATCCCGTCTTTC | lmxll |
| Marker4186 | GATAGACTCCATCCACTACAATATAAATGGTTGAAAATCAXXXXXXXXXXTTCAAACTTACTAAGTGCAACCAAACCGATTACATCCAAT | hkxhk |
| Marker4186 | GATAGACTCCATCCACTACAATATAAATGGTTGAAAATTAXXXXXXXXXXTTCAAACTTACTAAGTGCAACCAAACCGATTACATCCAAT | hkxhk |
| Marker4197 | GGTTTAGTGTGAAAGATAAATCATGAAGAAGTCTACTAATXXXXXXXXXXGTTTGCTTTCTCTCTGCTTGTTACCAAATACTTTTCAAGT | nnxnp |
| Marker4197 | GGTTTAGTGTGAAAGATAAATCATGAAGAAGTCTACTAATXXXXXXXXXXGTTTGCTTTCTCTCTGCTTGTTACCAAATACTTTTCGAGT | nnxnp |
| Marker4199 | GGGCTGTTCAGTAGCATATTTAGAGGCCCGTTTTTGTTTTXXXXXXXXXXGTTTCACACTCTCTCAGAAGATCTTGCTGCTCAACCTGGT | lmxll |
| Marker4199 | GGGCTGTTCGGTAGCATATTTAGAGGCCCGTTTTTGTTTTXXXXXXXXXXGTTTCACACTCTCTCAGAAGATCTTGCTGCTCAACCTGGT | lmxll |
| Marker4202 | TGTTCATGCCTTTCTCTTTCCCCAAAAATATTCCGCTTTCXXXXXXXXXXCGTCATTGAGAAAATGAAGAAATATGCTATTACTAACCTA | nnxnp |
| Marker4202 | TGTTCATGCCTTTCTCTTTCCCCAAAAATATTCCGCTTTCXXXXXXXXXXCGTCATTGAGAAAATGAAGAAATATGCTATTTCTAACCTA | nnxnp |
| Marker4204 | ATTTCATTTACTAAATTACTAAACGATAGCTGGCTGGAAAXXXXXXXXXXTATTTGGGTCACTATCGAATTGTCCAAATTATGGTGATTT | lmxll |
| Marker4204 | ATTTCATTTACTAAATTACTAAACGATAGCTGGCTGGAAAXXXXXXXXXXGATTTGGGTCACTATCGAATTGTCCAAATTATGGTGATTT | lmxll |
| Marker4205 | TGAAATAATGATGTGACTAATATAATAGTCCACATAAGGGXXXXXXXXXXGAGTAGGTATGAGTGCAACCATGCTACCAAATCAAGAATT | hkxhk |
| Marker4205 | TGAAATAATGATGTGGCTAATATAATAGTCCACATAAGGGXXXXXXXXXXGAGTAGGTATGAGTGCAACCATGCTACCAAATCAAGAATT | hkxhk |
| Marker4208 | GCATTGCTGACTTCTCAGACTGAGTACAAAGAGGAAGGACXXXXXXXXXXATTTTTGTTTCTCATCTTATAAAACTTAGTTCCCAGTTGA | lmxll |
| Marker4208 | GCATCGCTGACTTCTCAGACTGAGTACAAAGAGGAAGGACXXXXXXXXXXATTTTTGTTTCTCATCTTATAAAACTTAGTTCCCAGTTGA | lmxll |
| Marker4215 | GGTTCGGATTGTTATAATCTACACCCTTATAAAAATTTTGXXXXXXXXXXACACGCTCCAAAATCTCATAGGGCCCAATGTAACGAGGAC | lmxll |
| Marker4215 | GGTTTGGATTGTTATAATCTACACCCTTATAAAAATTTTGXXXXXXXXXXACACGCTCCAAAATCTCATAGGGCCCAATGTAACGAGGAC | lmxll |
| Marker4217 | CTCCAACTCAACTTTAGGGATGATTCCTATAAAACTCATAXXXXXXXXXXGTACCCCATTTATATTAGAGACCATGATTAGATGTAGCTT | lmxll |
| Marker4217 | CTCCAACTCAACTTTAGGGATGATTCCTATAAAACTCATAXXXXXXXXXXGTACCCCATTTATATTAGAGACCATGATCAGATGTAGCTT | lmxll |
| Marker4223 | TTTCCACATCTCTAAACAAGCCCCTAAAAGGGGTAATGAGXXXXXXXXXXACATCAAACAAAACACATGCCTGGCTACGAAAAGTCAGGT | lmxll |
| Marker4223 | TTTCCACATCTCTAAACAAGCCCCTAAAAGGGGTAATGAGXXXXXXXXXXACATCAAACAAAACACATGCCTGGCTACGAAAAGTTAGGT | lmxll |
| Marker4226 | TAATTCAACTCAATTATGTTATCTCATTCCTTTTTGTGTTXXXXXXXXXXTTGGTAATTTTGATGACACTTGCATAACGTGGGTAGGATT | nnxnp |
| Marker4226 | TAATTCAACTCAATTATGTTATCTGATTCCTTTTTTGGTTXXXXXXXXXXTTGGTAATTTTGATGACACTTGCATAACGTGGGTAGGATT | nnxnp |
| Marker4227 | TAAATCAGCGCTGGCTTCTTCTGTCCATTACTATATATGCXXXXXXXXXXAACTTACCTTTTATGGAACAAAACTATGCGTATATTAGTT | lmxll |
| Marker4227 | TAAATCAGCGCTGGCTTCTTCTGTCCATTACTATATATGCXXXXXXXXXXAATTTACCTTTTATGGAACAAAACTATGCGTATATTAGTT | lmxll |
| Marker4233 | ATACATATTTATATAGCATTCATGAAACATGATAAAGCACXXXXXXXXXXTTTCAGAACAGTGAACAAACAAATATCAGACAAGTCAAAT | lmxll |
| Marker4233 | ATACATATTTATATAGCATTCATGAATCATGATAAAGCACXXXXXXXXXXTTTCAGAACAGTGAACAAACAAATATCAGACAAGTCAAAT | lmxll |
| Marker4237 | TCATTTTCAAAAGGAAACTCACACTAAATAGTAAACAAGAXXXXXXXXXXTGATCTATTTTCTGTGCTGTTTATCTGAAGGCACCACCCA | hkxhk |
| Marker4237 | TCATTTTCAAAAGGAAACTCACACTAAATAGTAAACAAGAXXXXXXXXXXTGATATATTTTCTGTGCTGTTTATCTGAAGGCACCACCCA | hkxhk |
| Marker4240 | AGGTACCTACGCAACTACTACTCTCAATGCTGCTCATTGAXXXXXXXXXXTGCTCTGAGCATTGAAGATCTTGTTCATAATTGATGGGAT | lmxll |
| Marker4240 | AGGTACCTACGCAACTACTACTCTCAATGCTGCTCATTGAXXXXXXXXXXTGCTCTGAGCATCGAAGATCTTGTTCATAATTGATGGGAT | lmxll |
| Marker4246 | GGACATGGAGTATGTTCGCACTTGATGGCAGACAAAGTTGXXXXXXXXXXGGTACGGTATTGAGCCCATACCACTACCGATATCGGAAAT | nnxnp |
| Marker4246 | GGACATGGAGTATGTTCGCACTTGATGGCAGACAAAGTTGXXXXXXXXXXGGTACGGTATTGAGCTCATACCACTACCGATATCGGAAAT | nnxnp |
| Marker4247 | TGTGTCCATGTAGTTCTGTACTTGTTAGTGCCCTTCAATTXXXXXXXXXXTCTAACTGCATTGCATTTTCACAATCAAACTTTCTTTCTT | nnxnp |
| Marker4247 | TGTGTCCATGTAGTTCTGTACTTGTTAGTGCCCTTCAATTXXXXXXXXXXTCTAACTGCATTGAATTTTCACAATCAAACTTTCTTTCTT | nnxnp |
| Marker4248 | ATGTTTATGTGGCACTTTTGACATATCGGTTGGAGATGCTXXXXXXXXXXAGCCAACGCAAGTTTCTGATTCTCGAGGGAATGTTTCCGC | nnxnp |
| Marker4248 | ATGTTCATGTGGCACTTTTGACATATCGGTTGGAGATGCTXXXXXXXXXXAGCCAACGCAAGTTTCTGATTCTCGAGGGAATGTTTCCGC | nnxnp |
| Marker4254 | GTTTGAAATTGTAATGACTTTTACATTCATTATAGTAGACXXXXXXXXXXAAAGGGTAAAGTGTCGTTTTACCCTGGAAATAAAAGTCAT | hkxhk |
| Marker4254 | GTTTGAAATTGCAATGACTTTTACATTCATTATAGTAGACXXXXXXXXXXAAAGGGTAAAGTGTCGTTTTACCCTGGAAATAAAAGTCAT | hkxhk |
| Marker4256 | TATGGAAAATCAAATTCAGGCTTTATAGATAAGTTTGTTAXXXXXXXXXXTTTTCTATTCTCATATTCATATATATTGGCAGAACAAACT | lmxll |
| Marker4256 | TATGGAAAATCAAATTCAGGCTTTGTAGATAAGTTTGTTAXXXXXXXXXXTTTTCTATTCTCATATTCATATATATTGGCAGAACAAACT | lmxll |
| Marker4265 | GTGTTCCTTTCCATGGTGTAGTGGGCCTATAGAGACTACCXXXXXXXXXXGAAGAAGAAGAAGAAAATTACAATATCTGAACATATATAA | lmxll |
| Marker4265 | GTGTTCCTTTCCATGGTGTAGTGGGCCTATAGAGACTACCXXXXXXXXXXAGAGAAGAAGAAGAAAATTACAATATCTGAACATATATAA | lmxll |
| Marker4270 | AACGTGCGTTCCAACATAAGTGAAATTGAAAGCCCAACAAXXXXXXXXXXATTTCGTCATCTATTAGACATGCAAAAATAAAAGATTGCA | lmxll |
| Marker4270 | AACGTGCGTTCGAACATAAGTGAAATTGAAAGCCCAACAAXXXXXXXXXXATTTCGTCATCTATTAGACATGCAAAAATAAAAGATTGCA | lmxll |
| Marker4284 | CCGTAAAAGATTCTTCAGCTGATTGAGAAGTCTCTTCATGXXXXXXXXXXGCATCTTCACTAACTTTTGACAATTCAAACAAAGCTTGTC | nnxnp |
| Marker4284 | CCGTAAAAGATTCTTCAGCTGATTGAGAAGTCTCTTCATGXXXXXXXXXXGCATCTTCACTAACTCTTGACAATTCAAACAAAGCTTGTC | nnxnp |
| Marker4292 | AAGGACCGCAACTATGGCAATTGCATGGGAGTATATGCTAXXXXXXXXXXCATATGCTGTGAAGTTTGAGTAGCTTTAGGACTCTCCTAA | nnxnp |
| Marker4292 | AAGGACCGCAACTATGGCAATTGCATGGGAGTATATGCTAXXXXXXXXXXCATATGCTGCGAAGTTTGAGGAGCTTTAGGACTCTCCTAA | nnxnp |
| Marker4293 | TTCCTGGACGGACCATTCAATTGGGTTTCTTTCATTTTTCXXXXXXXXXXTCCGTACACTTTCACGCCCGAGGTTTTTATTTTTTTCTTC | lmxll |
| Marker4293 | TTCCTGGATGGACCATTCAATTGGGTTTCTTTCATTTTTCXXXXXXXXXXTCCGTACACTTTCACGCCCGAGGTTTTTATTTTTTTCTTC | lmxll |
| Marker4297 | ATTTTCTGTCAAAAGTAAGGATAAATTCGGAATTTCATCCXXXXXXXXXXGTTGGGTTTTATGGTAACCAGAAGGTGGTTGGAATCGGGA | lmxll |
| Marker4297 | ATTTTCTGTCAAAAGTAAGGATAAATTCGGAATTTCATCCXXXXXXXXXXGTTGGGTTTTATGGTAACCAGAAGGTGGTTGGAATTGGGA | lmxll |
| Marker4300 | TTCTAAATTGGAGGACAGTAAAATGCTTGAGGTATACATAXXXXXXXXXXTTATCATATAGTCTAACATGTAATGTTGTTAGACTTGCGG | nnxnp |
| Marker4300 | TTATAAATTGGAGGACAGTAAAATGCTTGAGGTATACATAXXXXXXXXXXTTATCATATAGTCTAACATGTAATGTTGTTAGACTTGCGG | nnxnp |
| Marker4311 | TCCAGATTTTGTATGACAAGAAAGTTTTACATGCGTTGTCXXXXXXXXXXACGGGGGGTCAATTGATTCAATCAAAAGCAAAGTGGTCTC | lmxll |
| Marker4311 | TCCAGATTTTGTATGACAAGAAAGTTTTACAGGCGTTGGCXXXXXXXXXXACGGGGGGTCAATTGATTCAATCAAAAGCAAAGTGGTCTC | lmxll |
| Marker4315 | GAGAAAAACTGACACTACCAAATGTTATTTCAACTGTTCAXXXXXXXXXXCATTTGCTTCATATGTCTTGCCAAAATGTATAGGGTACAG | nnxnp |
| Marker4315 | GAGAAAAACTGACACTACCAGATGTTATTTCAACAGTTCAXXXXXXXXXXCATTTGCTTCATATGTCTTGCCAAAATGCATAGGGTACAG | nnxnp |
| Marker4319 | GCCAAGGTTTGATTTCAACTTTGTGGATGTATGGTTCAAAXXXXXXXXXXTTTCAGTGATCATCTACCGAATTTGTTACTGAGTTTGTAC | lmxll |
| Marker4319 | GCCAAGGTTTGATTTCAACTTTGTGGATGTATGGTTCGAAXXXXXXXXXXTTTCAGTGATCATCTACCGAATTCGTTACTAAGTTTGTAC | lmxll |
| Marker4331 | TAGGCAGAAGGGGGACAAATGAAGAACGAGAGCAAAAAGGXXXXXXXXXXGCAATTTTTTGCTGCAATTTGCTTCATTCTCTATCTTGCA | nnxnp |
| Marker4331 | TAGGCAGAAGGGGGACAAATGAAGAACGAGAGCAAAAAAGXXXXXXXXXXGCAATTTTTTGCTGCAATTTGCTTCATTCTCTATCTTGCA | nnxnp |
| Marker4332 | TAAGAATGGTTACTTATCACATCATGTAGATTTGAGCCACXXXXXXXXXXTGATGAGATTGACAAAGGAAAAATAAATTGAGAAATAGAT | lmxll |
| Marker4332 | TAAGAATGGTTACTTATCACATCATGTAGATTTGAGCCACXXXXXXXXXXTTGATGAGATTGACAAAGGAAAAATAAATTGAGAAATAGA | lmxll |
| Marker4333 | GAAGACATCACAGGCTTGGACTAACTTCGACGGGCCTGGGXXXXXXXXXXAACCGGGGAGGTACCTAAGGTAGTGCTAATAACACTCAAA | hkxhk |
| Marker4333 | GAAGACATCACAGGCTTGGACTAACTCCGACGGGCCTGGGXXXXXXXXXXAACCGGGGAGGTACCTAAGGTAGTGCTAATAACACTCAAA | hkxhk |
| Marker4338 | AAACAAAAACAAAAAGAGTTTTTTGAGTTTTTTTTTTTTTXXXXXXXXXXGAGCTTGCTCGCGCATCATATTTGAATGCACGCCAAAGCA | lmxll |
| Marker4338 | AAACAAAAACAAAAAGAGTTTTTTGAGTATTTTTTTTTTTXXXXXXXXXXGAGCTTGCTCGCGCATCATATTTGAATGCACGCCAAAGCA | lmxll |
| Marker4340 | AAGAATTAGAGCTCCCATTCTAAAAAGTATATCTTATAAAXXXXXXXXXXCCCTGACTAAGCAGCACAGGTACCACAATACAGCTCCAGC | lmxll |
| Marker4340 | AAGAATTAGAGCTCCCATTCTAAAAAGTATCTCTTATAAAXXXXXXXXXXCCCTGACTAAGCAGCATAGGTACCACAGTACAGCTCCAGC | lmxll |
| Marker4345 | TTTGTGTGGCAGGAAACATATCTAATTTCCTCCAGTGAGGXXXXXXXXXXTTCAGTTGTGAATGTGCACTTTCTGCCCTATTTATAAACA | nnxnp |
| Marker4345 | TTTGTGTGGCAGGAAACATATCTAATTTCCTTCAGTGAGGXXXXXXXXXXTTCAGTTGTGAATGTGCACTTTCTGCCCTATTTATAAACA | nnxnp |
| Marker4361 | TGTGGGAAGTTTTTGGCATGATAGAGGAATTGAAATAATGXXXXXXXXXXTTTCTGCAAATTATGGTCTGCATGTAGATTATTTGGAGAG | lmxll |
| Marker4361 | TGTGGGAAGTTTTTGGCATGATAGAGGAATTGAAATAATGXXXXXXXXXXTTTCTGCAAATTATAGTCTGCATGTAGATTATTTGGAGAG | lmxll |
| Marker4364 | AAGACAAGAACCAAAAATCCTCCTTTGAATAAAAGGAAATXXXXXXXXXXAGATCTCCTAATCTTACATCATTCAGAACCATCGCTCACA | lmxll |
| Marker4364 | AAGACAAGAACCAAAAATCCCCCTTTGAATAAAAGGAAATXXXXXXXXXXAGATCTCCTAATCTTACATCATTCAGAACCATCGCTCACA | lmxll |
| Marker4368 | AAATCCAGAGTTTCATTGGGGATGCAGTTCGCAAATCGGGXXXXXXXXXXGAAATAAAACTAACCTGAAAAGACCATTTTACCCATAAAT | lmxll |
| Marker4368 | AAATCCAGAGTTTCATTTGGGGATGCAGTTTGCAAATCGGXXXXXXXXXXGAAATAAAACTAACCTGAAAAGACCATTTTACCCGTAAAT | lmxll |
| Marker4370 | AGAAACTATGCCGCTAATATAAGAATGCATCCAAGGATAAXXXXXXXXXXTAAGCTTTACCAATTCATTGACCATTTTTATTCTGTAGGC | lmxll |
| Marker4370 | AGAAACTATGCCGCTGATATAAGAATGCATCCAAGGATAAXXXXXXXXXXTAAGCATTACCAATTCATTGACCAATTTTATTCTGTAGGC | lmxll |
| Marker4372 | ATGCACCATCTTGAGAAGGGCGTTGTGCAACAAAGCAAAAXXXXXXXXXXGCTTTTCTTAGAATTCAACGCAGCATATCACAAACTTTGC | lmxll |
| Marker4372 | ATGCACCATCTTGAGAAGGGCGTTGTGCAACAAAGCAAAAXXXXXXXXXXGCTTTTCTTAGAATTCAACGTAGCATATCACAAACTTTGC | lmxll |
| Marker4380 | CAAAAAATATATATACAAAGGCTTTTTAGGAGCTATGGTCXXXXXXXXXXCATGTACAAACCACACAACACAAAATCATACAACCATTTT | nnxnp |
| Marker4380 | CAAAAAATATATATACAGAAAAGGCTTTTTAGGAGCTATGXXXXXXXXXXCATGTACAAACCACACAACACAAAATCATACAACCATTTT | nnxnp |
| Marker4382 | CACATTCTCGAGCCTTTCCCTGGATAAGCAAGAAGAAGCTXXXXXXXXXXCAAGCATGAGATTGCTGCTAATGCGTATAAGCTGGGATAC | hkxhk |
| Marker4382 | CACATTCTCGAGCCTTTCCCTGGATAAGCAAGAAGAAGCTXXXXXXXXXXCAAGCATGAGGTTGCTGCTAATGCGTATAAGCTGGGATAC | hkxhk |
| Marker4383 | TAAACAACAGCTGAATGAACAACAAACTTATCCTCTTTCTXXXXXXXXXXAACTATTTTCACAAACGTTTGTGCAAAAGTTCTTCTCCTA | lmxll |
| Marker4383 | TAAACAACAGCTGAATGAACAACAAACTTATCCTCTTTCTXXXXXXXXXXAACTATTTTCACAAATGTTTGTGCAAAAGTTCTTCTCCTA | lmxll |
| Marker4385 | GTTAGTGATACTACTTTTCATAAGGTGACACAACATTAGAXXXXXXXXXXGAAGAAGGTCTGCCTGTGTGGAATCGGAGGGTTCATATCA | lmxll |
| Marker4385 | GTTAGTGATTCTACTTTTCATAAGTTGACACAACATTAGAXXXXXXXXXXGAAGAAGGTCTGCCTGTGTGGAATCGGAGGGTTCATATCA | lmxll |
| Marker4388 | GTCTGACTTCTAATAAGAGCGATTGTGGCCGAGAGAGGAAXXXXXXXXXXAATCTGCTCCCTGGGAGGCTTGTGTTTGGTGTTGAACCCT | lmxll |
| Marker4388 | GTCTGACTTCTAATTAGAGCGATTGTGGCCGAGAGAGGAAXXXXXXXXXXATCTGCTCCGTGGGAGGCTTGTGTTTGGTGTTTGAACCCT | lmxll |
| Marker4394 | CCTTTATATTTATTTTGTGCAGCCGTCATTGGGTCTTGATXXXXXXXXXXTTTTGTATTATCAACGACGGTGAATTCAAAATACCGTCGT | lmxll |
| Marker4394 | CCTTTATATTTATTTTGTGCAGCCGTCATTGGGTCTTGATXXXXXXXXXXTTTTGTATTATCAACGACGGTCAATTCAAAATACCGTCGT | lmxll |
| Marker4399 | ATGGACATGAAATATGTACGATATGCAATGTTATTATCTGXXXXXXXXXXGCCATTTCATTCACTTCACCAAGTTACAAAAAGTCAGCAA | lmxll |
| Marker4399 | ATGGACATGAAATATGTACGATATGCAATGTGATTATCTGXXXXXXXXXXGCCATTTCATTCACTTCACCAAGTTACAAAAAGTCAGCAA | lmxll |
| Marker4405 | TCTCATTAGAATTTTCATGGGTAGATGACCCATGAGAAAAXXXXXXXXXXATAAGGCAAAGTTGTGTCAAGCCTTCCTCACCCATTTTTT | lmxll |
| Marker4405 | TCTCATTAGAATTTTCATGGGTAGATGACCCATGAGAAAAXXXXXXXXXXTAAGGCAAAGTTGTGTCAAGCCTTCCTCACCCATTTTTTT | lmxll |
| Marker4406 | TCAGCACGCAAAGAGTCAACATCAGGAGAAGAATCACAACXXXXXXXXXXGGGGAGAAAACAATAAAAACTAAGAGAAAGATATCACACT | hkxhk |
| Marker4406 | TCAGCACACAAAGAGTCAACATCAGGAGAAGAATCACAACXXXXXXXXXXGGGGAGAAAACAATAAAAACTAAGAGAAAGATATCACACT | hkxhk |
| Marker4408 | GACCCCTCCAACGTGTAGGGATTGGTCATGTGATCAACATXXXXXXXXXXGATGTCGGCTGTGATTGGAGTGGTGAATTTTGTGTACTAT | lmxll |
| Marker4408 | GACCCCTCCAACGAGTAGGGATTGGTCATGTGATCAACATXXXXXXXXXXGATGTCGGCTGTGATTGGAGTGGTGAATTTTGTGTACTAT | lmxll |
| Marker4409 | TTTGAAAATGAACCTAACAAACAGGTGCGCGGTTTATTATXXXXXXXXXXAGTTCTGAAGATGAACTTCATCAATAGCATGGCCCAATAT | lmxll |
| Marker4409 | TTTGAAAATGGACCTAACAAACAGGTGTGCGGTTTATCATXXXXXXXXXXAGTTCTGAAGATGAACTTCATCAATAGCATGGCCCAATAT | lmxll |
| Marker4412 | AAAAGAAAATTATCTACAAGGGAAAACACACAGTGATTGAXXXXXXXXXXTGAAAGCTTCCCAGGTGGATCAGAAACCTTCGAAATCATT | lmxll |
| Marker4412 | AAAGAAAATTATCTACAAGGGAAAACACACAGTGATTGAAXXXXXXXXXXTGAAAGCTTCCCAGGTGGATCAGAAACCTTCGAAATCATT | lmxll |
| Marker4417 | TTTCTAACAATCTGGATTGTTTTTGGTGATTGACAGAGACXXXXXXXXXXAGAACGTCAATCCTGAAGGAGGTTCGAATTTCTCCTTTTT | hkxhk |
| Marker4417 | TTTCTAACAATCTGGATTGTTTTTGGTGATTGACAGAGACXXXXXXXXXXGAGAACGTCAATCCTGAAGGAGGTTCGAATTTCTCCTTTT | hkxhk |
| Marker4424 | CTAAAGTCTGATAATTATCTTATTCTTATAGATTCCTTAGXXXXXXXXXXTCATAATGGTTCTTCTTGTTTCACTCCTTCTGAATTGAAA | hkxhk |
| Marker4424 | CTAAAGTCTGCGATAATTATCTTATTCTTATAGATTCCTTXXXXXXXXXXTCATAATGGTTCTTCTTGTTTCACTCCTTCTGAATTGGAA | hkxhk |
| Marker4433 | AATGGCCCCTACCCGGATGCTTTCATCATCATCTCTATCAXXXXXXXXXXATTCAGGACTATGGAAGTCCAGAGTTGATTGAGGTGAGGA | nnxnp |
| Marker4433 | AATGGCCCCTACCCGGATGCTTTCATCATCATCTCTATCAXXXXXXXXXXATTCAGGACTATGGAAGTCCAGAGCTGATTGAGGTGAGGA | nnxnp |
| Marker4434 | TGGAAGCTGTAATATGGCCTCAAAAATACCAGGATGCATTXXXXXXXXXXAGTAGCTTTTTCTCGTTGTTTATTTTTGTAAGGCTTTTTC | hkxhk |
| Marker4434 | TGGAAGCTGTAATATGGCCTCAAAAACACCAGGATGCATTXXXXXXXXXXAGTAGCTTTTTCTCGTTGTTTATTTTTGTAAGGCTTTTTC | hkxhk |
| Marker4440 | AACCCTTAGCATCATAAGACCATAAATATATACTACTTCAXXXXXXXXXXATTACTTTTCATCCTGTTGGAAAAAATAACAATAAAATTG | lmxll |
| Marker4440 | AACCCTTAGCATCATAAGACCATAATATATACTACTTCAAXXXXXXXXXXATTACTTTTCATCCTGTTGGAAAAAATAACAATAAAATTG | lmxll |
| Marker4443 | ACATTATTCTTCCATGTCGATACAGTCGAGACAACCCCTGXXXXXXXXXXAGAGGTTGAAATATGATTGTACAAACTAATGATGGTCAAC | lmxll |
| Marker4443 | ACATTATTCTTCCATGTTGATACAGTCGAGACAACCCCTGXXXXXXXXXXAGAGGTTGAAATATGATTGTACAAACAAATGATGGTCAAC | lmxll |
| Marker4444 | TCAAATTTCAAGACGACAACCACTTTTATAGATGAATCTCXXXXXXXXXXCTCCTCCTGCACGTTTCAATTCAACAAACACTTCTTCAAC | lmxll |
| Marker4444 | TCAAATTTCAAGACGACAACCACTTTTATAGATGAATCTCXXXXXXXXXXCTCCTCCCGCACGTTTCAATTCAACAAACACTTCTTCAAC | lmxll |
| Marker4446 | AAAGTTATTTTTGATGGTTGAAGGGATGTTCAAGCAAGCAXXXXXXXXXXGCCCATCTGAAGTAAGGTTAGATTCTTCTCTTTCAGTAGA | lmxll |
| Marker4446 | AAAGTTATTTTTGATGGTTGAAGGGATGTTCAAGCAAGCAXXXXXXXXXXGCCCATCTGAAATAAGGTTAGATTCTTCTCTTTCAGTAGA | lmxll |
| Marker4447 | ACATAAACAAAACGGCATCTGTGGCTATGCATTTTACCTCXXXXXXXXXXCAGGACACCATAGAAAGTAAGCACACCATAGACGGTAAAG | nnxnp |
| Marker4447 | ACATAAACAAAACGGCATCTGTGGCTATGCATTTTACCTCXXXXXXXXXXCAGGACACCATAGAAAGTAAGCATACCATAGACGGTAAAG | nnxnp |
| Marker4454 | TTCCCGTGTGTGCTGTTTTTGACTTTACTGAAAGTGATGAXXXXXXXXXXAAAGCAAAGGAAGAGTGTTTTTGGTTGATAGAATTTTATA | nnxnp |
| Marker4454 | TTCTCGTGTGTGCTGTTTTTGACTTTACTGAAAGTGATGAXXXXXXXXXXAAAGCAAAGGAAGAGTGTTTTTGGTTGATAGAATTTTATA | nnxnp |
| Marker4465 | GAAATAAGCAAAGAAGTTATTCAGCTGTTTCAGTAATCTTXXXXXXXXXXTTCTATTGCCTGAATAGACATGGCAGAACATGCAAAAGAT | lmxll |
| Marker4465 | GAAATAAGCAAAGAAGTTATTCAGCTGCTTCAGTAATCTTXXXXXXXXXXTTCTATTGCCTGAATAGACATGGCAGAACATGCAAAAGAT | lmxll |
| Marker4470 | TGTTATCCAAGTCCTTGATCAGTTGATCTGTGGTATTTAGXXXXXXXXXXACATAACAACTATCTGCCTTGATCTTCTTTATAGTTCATT | lmxll |
| Marker4470 | TGTTATCCAAGTCCTTGATCAGTTGATCTGTGGTATTTAGXXXXXXXXXXACAGAACAACTATCTGCCATGATCTTCTTTATAGTTCATT | lmxll |
| Marker4472 | GATACAAAACACGTTTTCGTTTTCTGACCAAATTGCCAAAXXXXXXXXXXTATATGATTGATTGTCACAACAAGTTATCACCAAAAAACT | nnxnp |
| Marker4472 | GATACAAAACACGTTTTCGTTTTCTGACCAAATTGCCAAAXXXXXXXXXXTATATGATTGATTGTCACAACAAGTTATCACCAAAAAGCT | nnxnp |
| Marker4474 | ATGGAGCTGCATCCACCTGGAGAATGGCACTTTTCACCAAXXXXXXXXXXTCTTAGTTCTTAGAAATCCATGAAGCATAAATGATTGATG | lmxll |
| Marker4474 | ATGGAGCTGCATCCACCTGGAGAATGGCACTTTTCACCAGXXXXXXXXXXTCTTAGTTCTTAGAAATCCATGAAGCATAAATGATTGATG | lmxll |
| Marker4481 | ATATTGCACTAAAATTTGGACGGGCTGCGCAAACGCAAAAXXXXXXXXXXATTCGTTTATCTTCTGATTCAAACAACGTATTTTCTTGTT | lmxll |
| Marker4481 | ATATTGCACTAAAATTTGGACGGGCTGCGCAAACGCAAAAXXXXXXXXXXATTCGCTTATCTTCTGATTCAAACAACGTATTTTCTTGTT | lmxll |
| Marker4483 | ATTTAGATTTTCAATGTGTTTTCCTTATTTACTTCAAATGXXXXXXXXXXCATACAGAGCTTACTATGCAAAAGAAGCTGTATGTAATAA | lmxll |
| Marker4483 | ATTTAGATTTTCAATGTGTTTTCCTTATTTACTTCAAATGXXXXXXXXXXCATACAGAGCTTACTATGCAAAAGAAGCTGTATGTGATAA | lmxll |
| Marker4497 | GCCTTGTATTTTGAGCTAATAAAATTGTGATTTGTTATGTXXXXXXXXXXGGAGGGTGATGCGGCAGATACCAGGGAGAATGCGCCCCCC | nnxnp |
| Marker4497 | GCCTTGTATTTTGAGCTAATAAAATTGTGATTTGTTATGTXXXXXXXXXXGGAGGGTGATGCTGCAGATACCAGGGAGAATGCGTCCCCC | nnxnp |
| Marker4501 | AAGCCTTAGCACTTTAGGATTTGGTCTCATTTTAGGGTTGXXXXXXXXXXAAAAAAGGTCTATAAAATCTACTACGATAATGAGCAGAAA | lmxll |
| Marker4501 | AAGCCTTAGCACTTTAGGATTTGGTCTCATTTTAGGGTTGXXXXXXXXXXAAAAAAAGTCTATAAAATCTACTACGATAATGAGCAGAAA | lmxll |
| Marker4506 | ATCCCCCCAAAAAATGAAAGGCTATCCTTATAATTGTATCXXXXXXXXXXTTATGTCCACAATTCACTTCAATGCTTCCACGTTGTAACC | lmxll |
| Marker4506 | ATCCCCCCAAAAAAATGCAAGGCTATCCTTATAATTGTATXXXXXXXXXXTTATGTCCACAATTCACTTCAATGCTTCCACGTTGTAACC | lmxll |
| Marker4514 | TTTAGATATGAACCAATTATCAGGCACGCATGTATCAATAXXXXXXXXXXCAATAATATGGTGTTTTTGAGTTTTTTTGGGTTTGGGTTG | nnxnp |
| Marker4514 | TTTAGATATGAACCAATTATCAGGTATGCATGTATCAATAXXXXXXXXXXCAATAATATGGTGTTTTTGAGTTTTTTTGGGTTTGGGTTG | nnxnp |
| Marker476 | CGCACATCCCTTCGGCCCGGAACTCCAGGCGGTGAGATTTXXXXXXXXXXTATGAAGCCACTTCTCATGCAGTATGGCTAAGGAATTTTA | abxcd |
| Marker476 | CGCACATCCCTTCGGCCCGGAACTCCAGGCCGTGAGATTTXXXXXXXXXXTATGAAGCCACTTCTCATGCAGTATGGCTAAGGAATTTTA | abxcd |
| Marker476 | CGCACATCCCTTTGGCCCGGAACTCCAGGCCGTGAGATTTXXXXXXXXXXTATGAAGCCACTTCTCATGCAGTATGGCTAAGGAATTTTA | abxcd |
| Marker476 | CGTACATCCCTTCGGCCCGGAACTCCAGGCCGTGAGATTTXXXXXXXXXXTATGAAGCCACTTCTCATGCAGTATGGCTAAGGAATTTTA | abxcd |
| Marker684 | AATACGCAAGTCCTAACAGTTTAGAACTATACGCTCTGCAXXXXXXXXXXTAATAATGGGAAATTCATGCGAATCTTATGGAGATCTATA | efxeg |
| Marker684 | AATACGCAAGTCCTAACAGTTCAGACCTATACGCTCTGCAXXXXXXXXXXTAATAATGGGAAATTCATGCGAATCTTATGGAGATCTACA | efxeg |
| Marker684 | AATACGCAAGTCCTAACAGTTTAGACCTATACGCTCTGCAXXXXXXXXXXTAATAATGGGAAATTCATGCGAATCTTATGGAGATCTATA | efxeg |
| Marker726 | AAGCTCTTTTACTGTATCAACCAAACACTCAATTAGATAGXXXXXXXXXXTAAAATGTACCGACTATATAAACTCATGCTTATTATCCCA | nnxnp |
| Marker726 | AAGCTCTTTTACTGTGTCAACCAAACACTCAATTAGATAGXXXXXXXXXXTAAAATGTACCGACTTTATAAACTCATGCTTATTATCCCA | nnxnp |
| Marker726 | AAGCTCTTTTACTGTGTCAACCAAACACTCAATTAGATAGXXXXXXXXXXTAAAATGTACCGACTATATAAACTCATGCTTATTATCCCA | nnxnp |
| Marker735 | ATTTTTTACACTAAAAGCCAACTTTGTAAAGGTTAGTGTAXXXXXXXXXXTGTGGCTTACATGAAGTGTCCTATGTGGTAGATAAATTGT | efxeg |
| Marker735 | ATTTTCTACACTAAAAGCCAACTTTGTAAAGGTTAGTGTAXXXXXXXXXXTGTGGCTTACATGAAGTGTTCTATGTGGTAGATAAATTGT | efxeg |
| Marker735 | ATTTTCTACACTAAAAGCCAACTTTGTAAAGGTTAGTGTAXXXXXXXXXXTGTGGCTTACATGAAGTGTCCTATGTGGTAGATAAATTGT | efxeg |
| Marker751 | AGACGTTATTACCCCTAATGACTTTTAGATTGTTCAGGGAXXXXXXXXXXTAAAACTTGGAAACAGGAGTTGGACTGCCTATGGTAATAT | nnxnp |
| Marker751 | AGACGTTATTACCCCTAATGACTTTTAGATTGTTCAGGGAXXXXXXXXXXAAAACTTGGAAACATGAGTTGGACCGCCTATGGTAATATT | nnxnp |
| Marker751 | AGACGTTATTACCCCTAATGACTTTTAGATTGTTCAGGGAXXXXXXXXXXTAAAACTTGGAAACATGAGTTGGACCGCCTATGGTAATAT | nnxnp |
| Marker762 | GCGGCGGATTATCTTTTTCTAAAGACATCAATTTTATAACXXXXXXXXXXTGAGGTCCCATTCTGAGTCAAAGGAACCTTCAACTCACAT | efxeg |
| Marker762 | GCGGCGGATTATCTTTTTCTAAAGCCATCAATTTTATAACXXXXXXXXXXTGAGGTCCCATTCTGAGTCAAAGGAACCTTCAACCCACAT | efxeg |
| Marker762 | GCGGCGGATTATCTTTTTCTAAAGACATCAATTTTATAACXXXXXXXXXXTGAGGTCCCATTCTGAGTCAAAGGAACCTTCAACCCACAT | efxeg |
| Marker791 | GAAGAATACATAACAAGTAACTGGGCAGTCATCTGGAATGXXXXXXXXXXCTAACACGATAAAAATTCGAGTCAATTTATCGTGACCCAT | lmxll |
| Marker791 | GAAGAATACATAACAAGTAACTGAGCAGTCATCTGGAATGXXXXXXXXXXCTAACACGATAAAAATTTGAGTCAATTTATCGTGACCCAT | lmxll |
| Marker791 | GAAGAATACATAACAAGTAACTGAGCAGTCATCTGGAATGXXXXXXXXXXCTAACACGGTAAAAATTTGAGTCAATTTATCGTGACCCAT | lmxll |
| Marker793 | GGTACTATTCATCAATTCATGAAAACGATTTTTGTTCATGXXXXXXXXXXTTTATTTTGTTTATACTACTTGAGCTAAACCTTTTTCATT | nnxnp |
| Marker793 | GGTACTATTCATCAATTCATGAATACGATTTTTGTTCATGXXXXXXXXXXTTTATTTTGTTTATACTACTTGAGCTAAACCTTTTTCATT | nnxnp |
| Marker793 | GGTACTATTCATCAATTCATGAATACGATTTTTGTTCATGXXXXXXXXXXTTTATTTTGTTTATACTACTTGAGCTAAACCTTTTTTATT | nnxnp |
| Marker843 | TCATGACTTATATTTCTGTTCTATATAGAGCAGCTTGATGXXXXXXXXXXTTTGGTTGCTCAAAACTGCAACACCAACAACATTCCCCGT | efxeg |
| Marker843 | TCATGACTTATATTTCTGTTCTATATAGAGCAGCTTGACGXXXXXXXXXXTTTGGTTGCTCAAAACGGCAACACCAACAACATTCCCCGT | efxeg |
| Marker843 | TCATGACTTATATTTCTGTTCTATATAGAGCAGCTTGACGXXXXXXXXXXTTTGGTTGCTCAAAACAGCAACACCAACAACATTCCCCGT | efxeg |
| Marker859 | TCAGAGAGTGGAGGGGCATGTTGCCATCCAGAGAGCCTTGXXXXXXXXXXGTGAGAGGGGTAGTTGATGGCGGGAAAGTAAGGCGTTGGG | efxeg |
| Marker859 | TCAGAGAGTGGAGGGGCATGTTACCATCCAGAGAGCCTTGXXXXXXXXXXGTGAGAGGGGTGGTTGATGGCGGGAAAGTAAGGCGTTGGG | efxeg |
| Marker859 | TCAGAGAGTGGAGGGGCATGTTGCCATCCAGAGAGCCTTGXXXXXXXXXXGTGAGAGGGGTGGTTGATGGCGGGAAAGTAAGGCGTTGGG | efxeg |
| Marker865 | ACCCAATATAAAAATTTTGGCAATACAATTCCCTATATTTXXXXXXXXXXTTCAGATGGTGAGGATAATATGTTAGGAAATTGGAGGTTT | efxeg |
| Marker865 | ACCCAATATAAAATTTTTGGCAATACAATTCCCTATATTTXXXXXXXXXXTTCAGATGGTGAAGATAATATGTTGGGAAATTGGAGGTTT | efxeg |
| Marker865 | ACCCAATATAAAATTTTTGGCAATACAATTCCCTATATTTXXXXXXXXXXTTCAGATGGTGAGGATAATATGTTAGGAAATTGGAGGTTT | efxeg |
| Marker913 | GCTTGTAGCGTGGTGTGTTGCTTATTTGCTTGGCGTGGCAXXXXXXXXXXTGCACCAACTTGAGGAGGAGTGTTGAAGTGAGTCTCCTAA | efxeg |
| Marker913 | GCTTGTAGCGTGGCGTGTTGCTTATTTGCTTGGCGTGGCAXXXXXXXXXXTGCACCAACTTGAGGAGGAGTGTTGAAGTGAGTCTCCTAA | efxeg |
| Marker913 | GCTTGTAGCGTGGCGTGTTACTTATTTGCTTGGCGTGGCAXXXXXXXXXXTGCACCAACTTGAGGAGGAGTGTTGAAGTGAGTCTCCTAA | efxeg |
| Marker929 | GGTACAATGAAACTGAAGAAAAACAAATCAATAGGTAAGTXXXXXXXXXXGCATTTCATAGGCATCCAAGAACATGTAACTTTCACCACA | efxeg |
| Marker929 | GGTACAATGAAACTGAAGAAAAACAAATCAATAGGTAAGTXXXXXXXXXXGCATTTCATAGGCATCCAAGAACATGTAACCTTCACCACA | efxeg |
| Marker929 | GGTACAATGAAACTGAAGAAAAACAAATCAATAGGTAAGTXXXXXXXXXXGCCTTTCATAGGCATCCAAGAACATGTAACCTTCACCATA | efxeg |
| Marker965 | AATAAAACAAATTCGCAAAAACAAAGGAGGGGCTCTTCATXXXXXXXXXXTAGAAGTACACTGTTCATACTATGTCATAGAAGTACACTG | lmxll |
| Marker965 | AATAAAACAAATTCGCAAAAACAAAGGAGGGGCTCTTCATXXXXXXXXXXTAGAAGTACACTGTTCATACCATGTCATAGAAGTACACTG | lmxll |
| Marker965 | AATAAAACAAATTCGCGAAAAAAAAGGAGGGGCTCTTCATXXXXXXXXXXTAGAAGTACACTGTTCATACTATGTCATAGAAGTACACTG | lmxll |
| Marker977 | AGATGCTCTTGATATCTGCAAAATTACAGAATAAGACATTXXXXXXXXXXAATTTAGAATTGAATACCCCTCTTCATTTTTGTACCTCAT | efxeg |
| Marker977 | AGATGCTCTTGATATCTGCAAAATTACAGAATAAGACATTXXXXXXXXXXCAATTTAGAATTGAATCCCCTCCACATTTTTGTACCTCAT | efxeg |
| Marker977 | AGATGCTCTTGATATCTGCAAAATTACAGAATAAGACATTXXXXXXXXXXCAATTTAGAATTGAATCCCCTCCACATTTGTGTACCTCAT | efxeg |
| Marker980 | CATTGGGAATTTCAGGTGCACGTGGGTATGGTATATGTAAXXXXXXXXXXAAATGTTGGTGGATCCCTATTTTTTGTTGTATTTATGAGT | nnxnp |
| Marker980 | CATTGGGAATTTCAGGTGCACGTGGGTATGGTATATGTAAXXXXXXXXXXAAATGTTGGTGGATCTCTATTTTTTGTTGTATTTATGGGT | nnxnp |
| Marker980 | CATTGGGAATTTTAGGTGCACGTGGGTATGGTATATGTAAXXXXXXXXXXAAATGTTGGTGGATCCCTATTTTTTGTTGTATTTATGAGT | nnxnp |
